# 1-Deoxynojirimycin promotes cardiac function and rescues mitochondrial cristae in mitochondrial hypertrophic cardiomyopathy

**DOI:** 10.1172/JCI164660

**Published:** 2023-07-17

**Authors:** Qianqian Zhuang, Fengfeng Guo, Lei Fu, Yufei Dong, Shaofang Xie, Xue Ding, Shuangyi Hu, Xuanhao D. Zhou, Yangwei Jiang, Hui Zhou, Yue Qiu, Zhaoying Lei, Mengyao Li, Huajian Cai, Mingjie Fan, Lingjie Sang, Yong Fu, Dong Zhang, Aifu Lin, Xu Li, Tilo Kunath, Ruhong Zhou, Ping Liang, Zhong Liu, Qingfeng Yan

**Affiliations:** 1 College of Life Science, Zhejiang University, Hangzhou, Zhejiang, China.; 2 The First Affiliated Hospital, Zhejiang University School of Medicine, Hangzhou, Zhejiang, China.; 3 Institute of Translational Medicine, Zhejiang University, Hangzhou, Zhejiang, China.; 4 Westlake Laboratory of Life Sciences and Biomedicine, School of Life Sciences, Westlake University, Hangzhou, Zhejiang, China.; 5 The Children’s Hospital of Zhejiang University School of Medicine, Hangzhou, Zhejiang, China.; 6 Institute for Stem Cell Research, School of Biological Sciences, The University of Edinburgh, Edinburgh, United Kingdom.; 7 Key Laboratory for Cell and Gene Engineering of Zhejiang Province, Hangzhou, Zhejiang, China.

**Keywords:** Cardiology, Stem cells, Cardiovascular disease, Mitochondria, iPS cells

## Abstract

Hypertrophic cardiomyopathy (HCM) is the most prominent cause of sudden cardiac death in young people. Due to heterogeneity in clinical manifestations, conventional HCM drugs have limitations for mitochondrial hypertrophic cardiomyopathy. Discovering more effective compounds would be of substantial benefit for further elucidating the pathogenic mechanisms of HCM and treating patients with this condition. We previously reported the *MT-RNR2* variant associated with HCM that results in mitochondrial dysfunction. Here, we screened a mitochondria-associated compound library by quantifying the mitochondrial membrane potential of HCM cybrids and the survival rate of HCM-induced pluripotent stem cell–derived cardiomyocytes (iPSC-CMs) in galactose media. 1-Deoxynojirimycin (DNJ) was identified to rescue mitochondrial function by targeting optic atrophy protein 1 (OPA1) to promote its oligomerization, leading to reconstruction of the mitochondrial cristae. DNJ treatment further recovered the physiological properties of HCM iPSC-CMs by improving Ca^2+^ homeostasis and electrophysiological properties. An angiotensin II-induced cardiac hypertrophy mouse model further verified the efficacy of DNJ in promoting cardiac mitochondrial function and alleviating cardiac hypertrophy in vivo. These results demonstrated that DNJ could be a potential mitochondrial rescue agent for mitochondrial hypertrophic cardiomyopathy. Our findings will help elucidate the mechanism of HCM and provide a potential therapeutic strategy.

## Introduction

Hypertrophic cardiomyopathy (HCM) is a common cause of sudden cardiac death in young people. It manifests as typical asymmetric septal hypertrophy of the left ventricle (1–3). Most HCM is inherited as an autosomal-dominant trait and is attributed to mutations in sarcomeric genes (4, 5). HCM-relevant sarcomeric gene mutations can cause disorganization of sarcomeres, further leading to decreased myofilament Ca^2+^ sensitivity and inefficient cellular ATP utilization. In contrast, some familial HCM is caused by mitochondrial genomic mutations, termed mitochondrial hypertrophic cardiomyopathy, and is transmitted maternally (6–8). Mitochondrial HCM exhibits the common manifestations of HCM but has a distinct and complex underlying pathophysiology. Mutations in genes in mitochondrial DNA (mtDNA) contribute to mitochondrial defects, deficient ATP synthesis efficiency in particular, and unbalanced calcium homeostasis as the fundamental cause of mitochondrial HCM. Current drug therapeutics for patients with HCM are focused on general symptomatic management. However, due to heterogeneity in the clinical manifestations, conventional drugs exhibit limitations when facing some types of mitochondrial HCM. Given the efficacy barrier of the current treatments, the identification of more effective therapeutics that target the underlying pathogenic mechanisms would be of clear benefit for patients with these intractable cases of HCM.

Mitochondrial hypertrophic cardiomyopathy is mainly characterized by mutations in mitochondrial genes (6, 9). Since the first HCM-associated *MT-TL1* mutation was demonstrated in 1991, multiple HCM clinical cases have been attributed to mtDNA mutations (10). The mutation or deletion of mtDNA can induce constitutive damage to mitochondrial integrity and lead to serious mitochondrial defects and pathological phenotypes (11, 12). Mitochondrial HCM has now become a recognized class of mitochondrial disease. Ongoing pharmacological interventions for HCM, such as β-blockers, diltiazem, and verapamil, mainly inhibit adrenergic signaling to reduce heart rate or target L-type calcium channels, ryanodine receptors, and sodium/calcium exchange pumps to decrease the intracellular calcium caused by arrhythmogenic HCM (13, 14). These therapeutic medicines can be effective in most cases caused by sarcomere-related gene mutations (15); however, their efficacy for some mitochondrial HCM remains limited and less studied (7, 16–20). Considering that functional impairment of mitochondria plays a leading role in many of these mitochondrial diseases, restoration of mitochondrial fitness could be a potent therapeutic strategy (21–23).

Given the difficulty in specifically editing the mitochondrial genome in mouse models, constructing patient-derived cell lines that carry patient-specific mitochondrial mutations is often considered a key strategy for mitochondrial drug screening (24). These typically include the construction of patient-specific cytoplasmic hybrid cells (cybrids) and/or induced pluripotent stem cell-derived cardiomyocytes (iPSC-CMs) as the ideal approach (24–26). Compared with the parent cells, cybrids have the same nuclear genomic background but carry the donor’s specific mitochondrial genome, allowing cybrids to mimic the pathological effects of the specific mtDNA mutation. iPSCs are generated from the patient’s own somatic cells that have been genetically reprogrammed (27). These can then be differentiated into iPSC-CMs using robust protocols (28). iPSC-CMs display complex cardiac phenotypes, including electrophysiological responsiveness and calcium handling, which provide an ideal platform for preclinical testing (29–31). Cybrids provide an easy-to-culture cell model for large-scale screening, while iPSC-CMs represent a powerful tool for investigating more physiological outcomes for advanced drug evaluation. Establishing a strategy combining the benefits of cybrids and iPSC-CM methods may advance the progress of mitochondria-targeted drug discovery for mitochondrial diseases.

Mitochondria play an indispensable role in cellular energy management and calcium handling. The changes in mitochondrial cristae are tightly associated with mitochondrial function (32). An optimal cristae shape is a determinant of efficient oxidative phosphorylation (OXPHOS) (33). The respiratory chain supercomplexes (RCSs) of OXPHOS are integrated into the inner mitochondrial membrane (IMM) along the mitochondrial cristae (34). Nuclear gene-encoded dynamin-related large GTPase Optic atrophy 1 (OPA1) governs cristae biogenesis and remodeling as a master regulator of cristae shape (35). Oligomerized OPA1 safeguards the cristae junction (CJ) number and stability, thereby promoting the stability of RCSs and respiratory efficiency (32, 36, 37). Depletion of OPA1 leads to cristae disorganization and related mitochondrial dysfunction (32, 36). Conversely, the transgenic overexpression of OPA1 in mice improves mitochondrial activity (33, 38, 39). This critical physiological capability suggests the potential for OPA1 modulation in mitochondria-targeted clinical therapies.

Previously, we identified the *MT-RNR2* mutation as a molecular basis for HCM (40–42). Here, we established a 2-step drug screening process using cybrids and iPSC-CMs from patients with HCM to identify an effective mitochondrial rescue agent, and we further explored the corresponding mechanism. Collectively, our study verifies the pathogenic mechanism of mitochondrial hypertrophic cardiomyopathy via mitochondrial rescue and provides an accessible preclinical platform for personalized drug screening.

## Results

### Compound screens based on a cybrid-coupled iPSC-CM model for mitochondrial hypertrophic cardiomyopathy.

**Figure 1:**
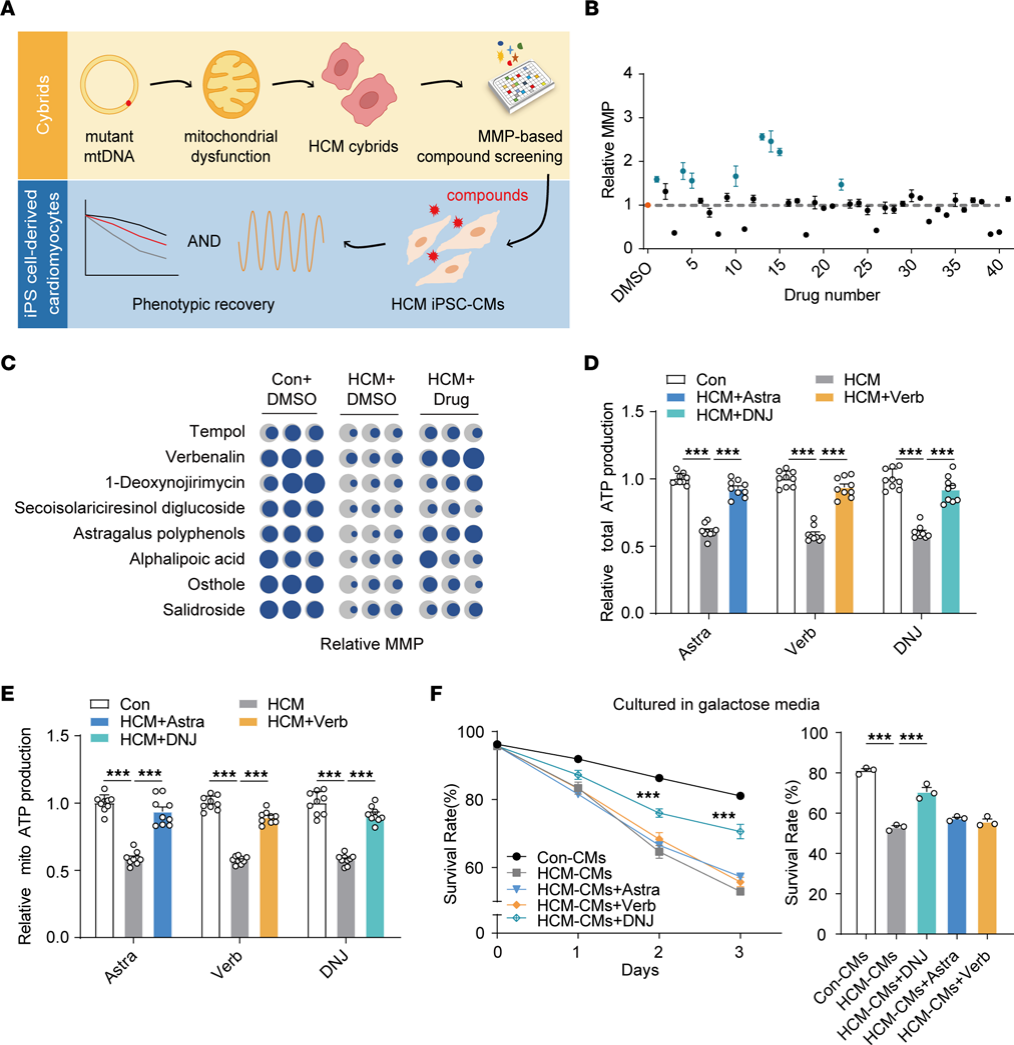
Compound screening method in HCM cybrids and HCM iPSC-CMs with mutant mtDNA. (**A**) Schematic of the 2-step drug screening in HCM cybrids and HCM iPSC-CMs with mutant mtDNA. (**B**) Screening of 41 chemical compounds from the mitochondrial drug bank (all 30 μM in 0.1% DMSO) for MMP analysis in 1 HCM cybrid cell clone with the *MT-RNR2* mutation. HCM cybrids treated with 0.1% DMSO (orange) were used as baseline. Values represent the mean ± SEM. *n* = 3 biological replicates. 2-tailed *t* test. Top candidates (*P* < 0.05) were highlighted in green. (**C**) Analysis of MMP (dark blue) from selected compounds. The MMP of 143B is shown in grey. Con, control. (**D**) Examination of total ATP production in response to DNJ, astragalus polyphenols (Astra), or verbenalin (Verb) administration. Values represent the mean ± SEM. *n* = 3 biologically independent experiments in 3 lines. 1-way ANOVA followed by Tukey’s test. ***P < 0.001. (**E**) Relative mitochondrial ATP production was measured using recording buffer (containing 5 mM 2-DG and 5mM pyruvate). Values represent the mean ± SEM. *n* = 3 biologically independent experiments in 3 lines. 1-way ANOVA followed by Tukey’s test. ***P < 0.001. (**F**) Galactose-induced cell death assay in HCM iPSC-CMs. Time course (left) and survival rate quantification (right) are shown as the mean ± SEM, *n* = 3 biologically independent experiments. 2-way ANOVA analysis. ***P < 0.001. Data are representative of 3 independent experiments.

We previously reported a 4-generation HCM family with the *MT-RNR2* mutation (Supplemental Figure 1, A and B; supplemental material available online with this article; https://doi.org/10.1172/JCI164660DS1) (40). To advance a pharmacological rescue strategy for the patients from this pedigree, we decided to apply chemical genetic screens. We first generated suitable patient-derived cell models, including cybrids, iPSCs, and iPSC-CMs carrying the same pathological mtDNA mutation (Supplemental Figure 1, C and D) (41, 42). The mitochondrial membrane potential (MMP) is a key indicator of cell status. It provides a representation of the main proton electrochemical gradient that accounts for mitochondrial respiratory energy. Here, it was used to validate the ability of pathological mimicry in both HCM cybrids and iPSC-CMs (Supplemental Figure 1, E and F). These results verified their modeling utility, which was consistent with our previous findings (41, 42). The HCM iPSC-CMs retained the patient’s integrated genomic information and could successfully recapitulate the disease phenotype in vitro. HCM cybrids specifically carried the ectopic mitochondrial genome along with a standard nuclear background. We considered that combining HCM cybrids and iPSC-CMs could be a feasible strategy for drug screening for this specific mutation and potentially for other mitochondrial mutations (Figure 1A). We designed patient-derived cybrids for use first in primary compound screening, where the MMP index was used to identify any mitochondrial benefit from the candidates, and then, second, in validating the physiological efficacy of these identified candidates in HCM iPSC-CMs.

The easy-to-culture patient-specific cybrids all carry the same nuclear background, making them an ideal model for primary mitochondrial drug screening (Supplemental Figure 1G). We narrowed down the original mitochondrial targeting compound library (L5300) from TargetMol to 41 chemical compounds by checking their potential to improve mitochondrial fitness from previous references (Supplemental Table 1). The results were displayed using a fluorescence monitor for the MMP, where 8 compounds were initially found to efficiently rescue the MMP in 1 HCM cybrid cell clone (Figure 1B). We subsequently and separately verified the efficacy of these 8 candidates in 3 cell clones. The results showed that only 3 candidates, 1-deoxynojirimycin (DNJ), astragalus polyphenols, and verbenalin, could robustly increase the level of the MMP compared with their basal counterparts (Figure 1C). To confirm their capacity to benefit mitochondrial health, we examined cellular ATP production and mitochondrial ATP production separately with these 3 chemicals (Figure 1, D and E). The results confirmed that DNJ, astragalus polyphenols, and verbenalin all had the potential to rescue the mitochondrial dysfunction caused by the *MT-RNR2* mutation.

To further validate the efficacy of the 3 aforementioned candidates at the cellular level, we applied patient-specific iPSC-derived cells in subsequent tests. HCM iPSC-CMs were considered more suitable for preclinical tests than HCM cybrids due to their ability to recapitulate more HCM phenotypes. The pathological mimicry CMs were differentiated using a monolayer differentiation protocol as described previously (28), with minor changes (Supplemental Figure 2A). The generated iPSC-CMs exhibited positive staining of the cardiac-specific markers α-actinin; myosin regulatory light chain 2, ventricular/cardiac muscle isoform (MLC2v); and myosin regulatory light chain 2, atrial isoform (MLC2a) (Supplemental Figure 2B), and no obvious changes in cardiac differentiation efficiency were observed.

We then applied the galactose-induced cell death assay to identify changes in the mitochondrial state under different candidate treatments. Galactose-cultured iPSC-CMs lost the ability to produce ATP by glycolysis and were forced to acquire the majority of the required energy for survival directly from mitochondria. We considered that any chemical drug that passed this demanding test would be highly competitive as a candidate for the indicated patients with HCM. Intriguingly, only DNJ exerted a sufficiently marked ability to rescue the survival rate of the HCM iPSC-CMs, while the other chemical molecules did not markedly reduce cell death (Figure 1F). These results suggest that DNJ could benefit mitochondrial function to augment cell survival under galactose culture conditions and could be a potent therapeutic chemical for mitochondrial hypertrophic cardiomyopathy.

### DNJ rescues abnormal electrophysiological properties and calcium handling in HCM iPSC-CMs.

**Figure 2:**
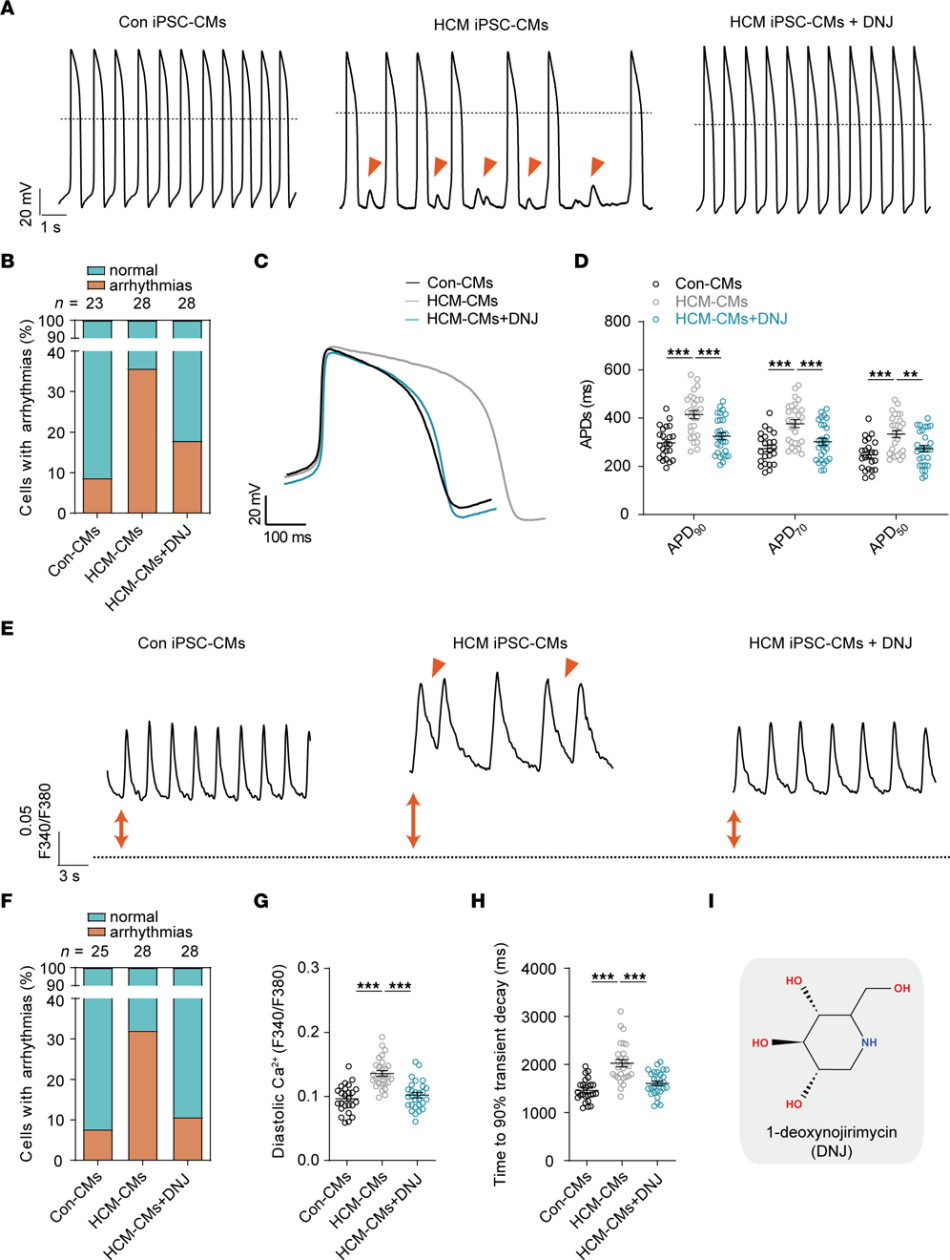
DNJ mitigates abnormal electrophysiological properties and calcium handling properties in HCM iPSC-CMs. (**A**) Representative action potential tracings of ventricular-like myocytes derived from Control (Con) iPSC-CMs, HCM iPSC-CMs and HCM iPSC-CMs + DNJ, respectively. Red arrow indicates irregular rhythm. (**B**) Quantification of cells with arrhythmias (Con: *n* = 23 in 2 cell lines; HCM: *n* = 28 in 2 cell lines; HCM+DNJ: *n* = 28 in 2 cell lines). (**C**) Representative action potential tracings from Con iPSC-CMs, HCM iPSC-CMs and HCM iPSC-CMs + DNJ. (**D**) Scatter dot plot to compare APD_50_, APD_70_ and APD_90_ (Con: *n* = 23 in 2 cell lines; HCM: *n* = 28 in 2 cell lines; HCM+DNJ: *n* = 28 in 2 cell lines). Values represent the mean ± SEM. 2-way ANOVA analysis. **P < 0.01, ***P < 0.001. (**E**) Representative raw traces of Fura-2 ratio-metric calcium signaling. Red arrow indicates abnormal Ca^2+^ handling events. (**F**) Quantification of cells with arrhythmias (Con: *n* = 25 in 2 cell lines; HCM: *n* = 28 in 2 cell lines; HCM+DNJ: *n* = 28 in 2 cell lines). (**G** and **H**) Scatter dot plot to compare diastolic Ca^2+^ (**G**) and decay times (**H**) (Con: *n* = 25 in 2 cell lines; HCM: *n* = 28 in 2 cell lines; HCM+DNJ: *n* = 28 in 2 cell lines), respectively. Values represent the mean ± SEM. 1-way ANOVA followed by Tukey’s test. ***P < 0.001. (**I**) Chemical structure of DNJ. For each group, data were collected from 2 different iPSC lines and at least 3 batches of differentiation.

We then sought to further investigate the potential of the 3 chemicals to improve the electrophysiological properties of patient-specific iPSC-CMs. The action potentials were recorded by single-cell patch clamp from control and HCM iPSC-CMs. The key parameters of the action potentials were quantified and compared, including maximal diastolic potential (MDP); overshoot; action potential amplitude (APA); action potential duration at 50%, 70%, and 90% (APD_50_, APD_70_ and APD_90_); maximal upstroke velocity (V_max_); SD of peak-peak intervals; and beating rate (Figure 2, A–D, Supplemental Figure 2, C–H, and Supplemental Table 2). Control iPSC-CMs showed a normal action potential profile. In contrast, HCM patient-specific iPSC-CMs exhibited an arrhythmic phenotype (Figure 2, A and B). Interestingly, we found that treatment with DNJ effectively rescued the arrhythmic phenotype observed in HCM iPSC-CMs (Figure 2B). Moreover, HCM iPSC-CMs showed significantly prolonged APDs compared with controls, which was markedly normalized by DNJ treatment (Figure 2, C and D). However, treatment with astragalus polyphenols or verbenalin had minimal effects on rescuing the abnormal action potential phenotypes in HCM iPSC-CMs (Supplemental Figure 3, A–H and Supplemental Table 3). Taken together, these results suggest that DNJ can restore the abnormal electrophysiological properties of HCM iPSC-CMs by rescuing mitochondrial function.

Calcium (Ca^2+^) directs cardiac excitation-contraction coupling together with the tightly regulated dynamics of intracellular Ca^2+^ and is strongly associated with the rhythmic beating of the heart (43). Elevated intracellular Ca^2+^ and dysfunctional Ca^2+^ cycling contribute to the pathogenesis of HCM (44, 45). Therefore, reestablishing Ca^2+^ homeostasis could, in turn, benefit the health of the heart (46). Detecting the alteration of Ca^2+^ cycling in HCM iPSC-CMs by Fura-2 imaging, we observed elevated abnormal arrhythmia-like Ca^2+^ transients (Figure 2, E and F). After treatment with DNJ, the abnormal Ca^2+^ handling events were decreased. Diastolic Ca^2+^ of the HCM groups was significantly higher than that of the controls, while the normalized decay time of HCM iPSC-CMs was markedly prolonged compared with that of the control group (Figure 2, G and H). Again, treatment with DNJ efficiently rescued the ectopic calcium flux of the HCM iPSC-CMs and decreased the diastolic calcium and decay times. Other parameters, including time to peak, Ca^2+^ amplitude, maximal rising rate, and maximal decay rate, were not different among the 3 groups (Supplemental Figure 4, A–D). All these results confirmed the potential therapeutic role of DNJ in our patients with HCM (Figure 2I).

### DNJ alleviates physiological defects in HCM iPSC-CMs.

**Figure 3:**
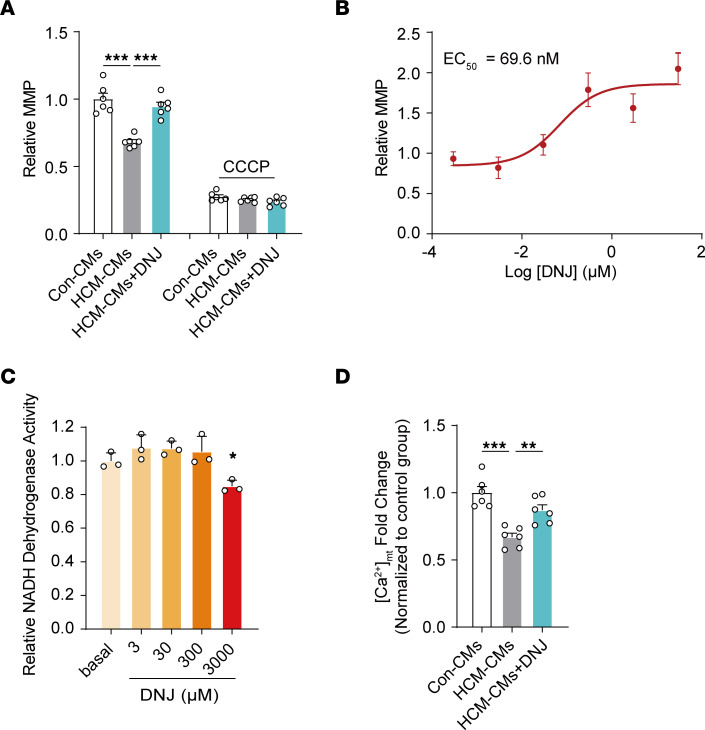
DNJ acts as a potential agent for HCM treatment. (**A**) Measurement of MMP analysis. *n* = 3 biologically independent experiments in 2 cell lines. Values represent the mean ± SEM, 1-way ANOVA followed by Tukey’s test. ***P < 0.001. (**B**) Representative concentration-response curves are shown with MMP as an indicator. *n* = 3 biologically independent experiments. Values represent the mean ± SEM. Data are representative of 3 independent experiments. (**C**) Cardiomyocytes were incubated with indicated concentrations of DNJ for the indicated time periods. Cell growth was determined using a CCK8 assay. *n* = 3 biologically independent experiments. Values represent the mean ± SEM. 1-way ANOVA followed by Tukey’s test. *P < 0.05. (**D**) Analysis of mitochondrial calcium by RHOD-2 indicators in iPSC-CMs. *n* = 3 biologically independent experiments in 2 cell lines. Values represent the mean ± SEM. 1-way ANOVA followed by Tukey’s test. **P < 0.01, ***P < 0.001.

To further evaluate the efficacy of DNJ in cardiomyocytes, we tested the other substantial physiological performances upon DNJ treatment. Consistent with the findings in the cybrids, DNJ restored the MMP of HCM iPSC-CMs (Figure 3A). DNJ had a half-maximal effective concentration of approximately 69.6 nM (Figure 3B) and showed low toxicity up to a concentration of 3 mM (Figure 3C). For HCM cybrids, the half-maximal effective concentration for MMP rescue was approximately 4.5 nM and showed low toxicity, even up to 30 mM (Supplemental Figure 5, A and B). Taken together, these results suggest that DNJ can be a potential medicine for HCM treatment.

Mitochondria have also been revealed to serve as active buffers during cellular calcium handling, especially in cardiomyocytes. Researchers have also claimed that mitochondrial calcium uptake depends on MMP, due to the electrochemical proton gradient creating a huge driving force. Considering the benefit of MMP in response to DNJ treatment (Figure 3A), we reasoned that DNJ could improve the calcium homeostasis of HCM iPSC-CMs by enhancing the mitochondrial viability of calcium buffering. To test this notion, we therefore monitored the calcium concentration in mitochondria and observed that [Ca^2+^]_mito_ in HCM iPSC-CMs was approximately 67% of Con iPSC-CMs and [Ca^2+^]_mito_ in the DNJ group had increased to 87% that of the control group (Figure 3D). Similar results of mitochondrial calcium were also consistently replicated in the model of HCM cybrids (Supplemental Figure 5, C and D). We found that a rise in cytosolic calcium consistently occurred in HCM iPSC-CMs but was attenuated upon DNJ treatment by enhancing the mitochondrial calcium uptake ability (Supplemental Figure 5E). This further confirmed the physiological efficacy of DNJ. Combining these series of results, we show that DNJ enhanced the mitochondrial capacity of calcium buffering, which helped to maintain the cellular calcium homeostasis of HCM iPSC-CMs, thereby facilitating increased potency in the performances of cardiomyocytes.

**Figure 4:**
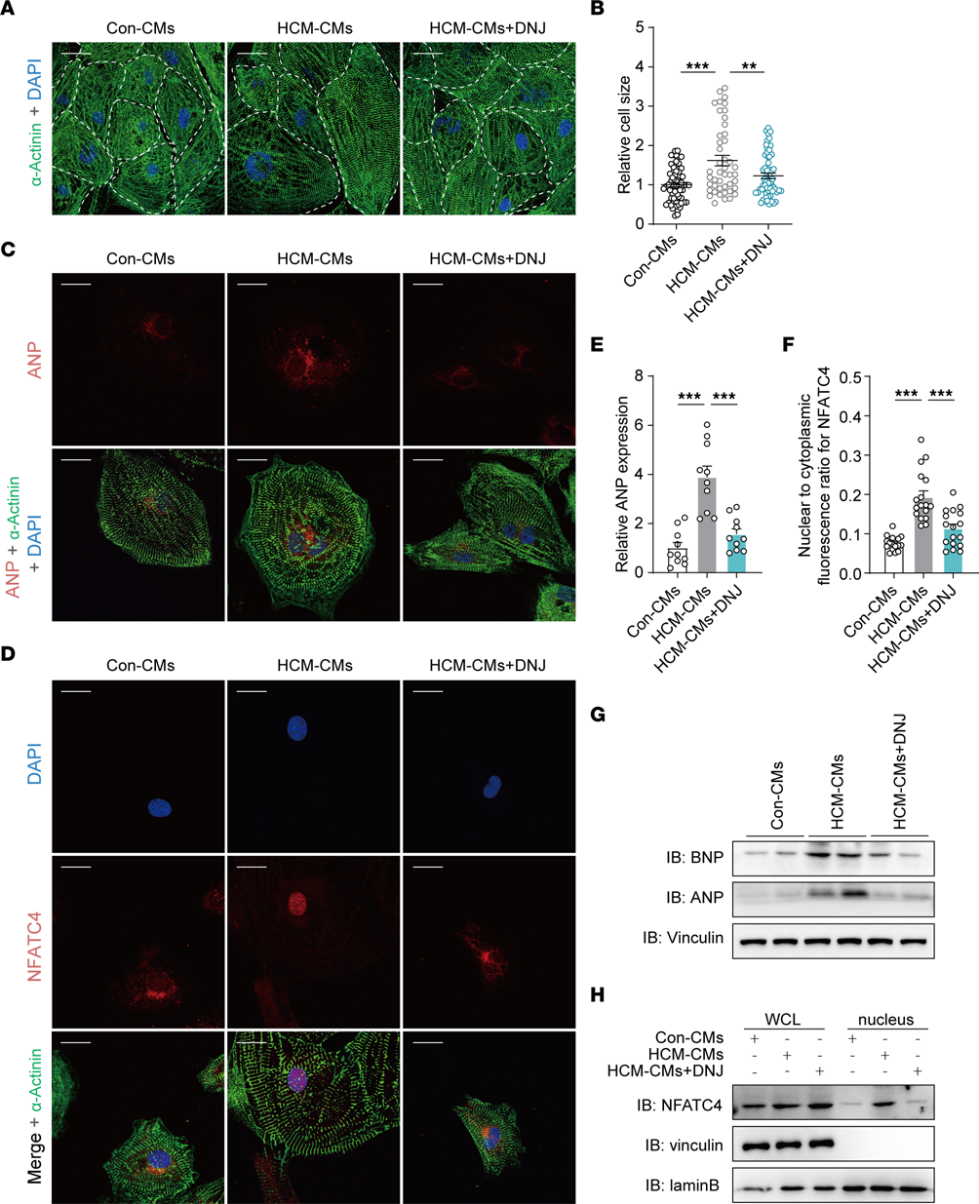
DNJ reverses HCM phenotype. (**A** and **B**) Representative images of iPSC-CMs stained for α-actinin immunofluorescence and quantification of cell size. Con: *n* = 64, HCM: *n* = 45, HCM + DNJ: *n* = 60 in 2 cell lines. Values represent the mean ± SEM. 1-way ANOVA followed by Tukey’s test. **P < 0.01, ***P < 0.001. Scale bar, 40 μm. (**C**–**F**) Representative immunofluorescence staining revealed changed ANP expression (**C**) and NFATC4 location (**D**) in the α-actinin–positive iPSC-CMs. Quantification of ANP expression (*n* = 10 in 2 cell lines) (**E**) and analyzation of the colocation between DAPI and NFATC4 (*n* = 17 in 2 cell lines) (**F**). Values represent the mean ± SEM. 1-way ANOVA followed by Tukey’s test. ***P < 0.001. Scale bar, 40 μm. (**G**) Western blotting of ANP and BNP. Vinculin is shown as a loading control. (**H**) Western blot detection of NFATC4 in the purified nucleus with the indicated protein markers (laminB for nucleus).

Physiologically, cardiomyocyte enlargement is a key pathological hallmark of HCM. This is regarded as a compensation effect. Our results showed that the average size of HCM iPSC-CMs was approximately 62% larger than that of controls based on α-actinin immunostaining (Figure 4, A and B). However, upon supplementation with DNJ, the cell size of HCM iPSC-CMs showed a 39% reduction. In addition to cell hypertrophy, increased nuclear translocation of NFATC4 and ectopic expression of ANP and BNP are considered molecular biomarkers of HCM (Figure 4, C–H). As expected, DNJ attenuated the expression of ANP and BNP in HCM iPSC-CMs (Figure 4, C, E and G) and suppressed the nuclear translocation of NFATC4 (Figure 4, D, F and H). These findings provide insight into the potential mechanisms by which DNJ can effectively alleviate the symptoms of HCM and thereby become a potent candidate for personalized clinical application for the indicated patients with mitochondrial (*MT-RNR2* mutation) HCM and, potentially, for other related conditions.

We also performed functional assays to confirm the effect of DNJ on the control groups. The results showed that DNJ did not have a significant effect on MMP (Supplemental Figure 6A) or ATP production (Supplemental Figure 6, B and C) in the control cybrid group. In addition, there were no marked changes in mitochondrial function (Supplemental Figure 6, D and E), electrophysiological properties (Supplemental Figure 7, A–H and Supplemental Table 4), or calcium handling (Supplemental Figure 8, A–H) upon DNJ treatment in the control iPSC-CMs. These results suggest that DNJ can restore the HCM pathological phenotype by improving impaired mitochondrial function and will not over-tune healthy individuals, which further emphasizes the potential of DNJ in clinical transformation.

### OPA1 is identified as the molecular target of DNJ.

**Figure 5:**
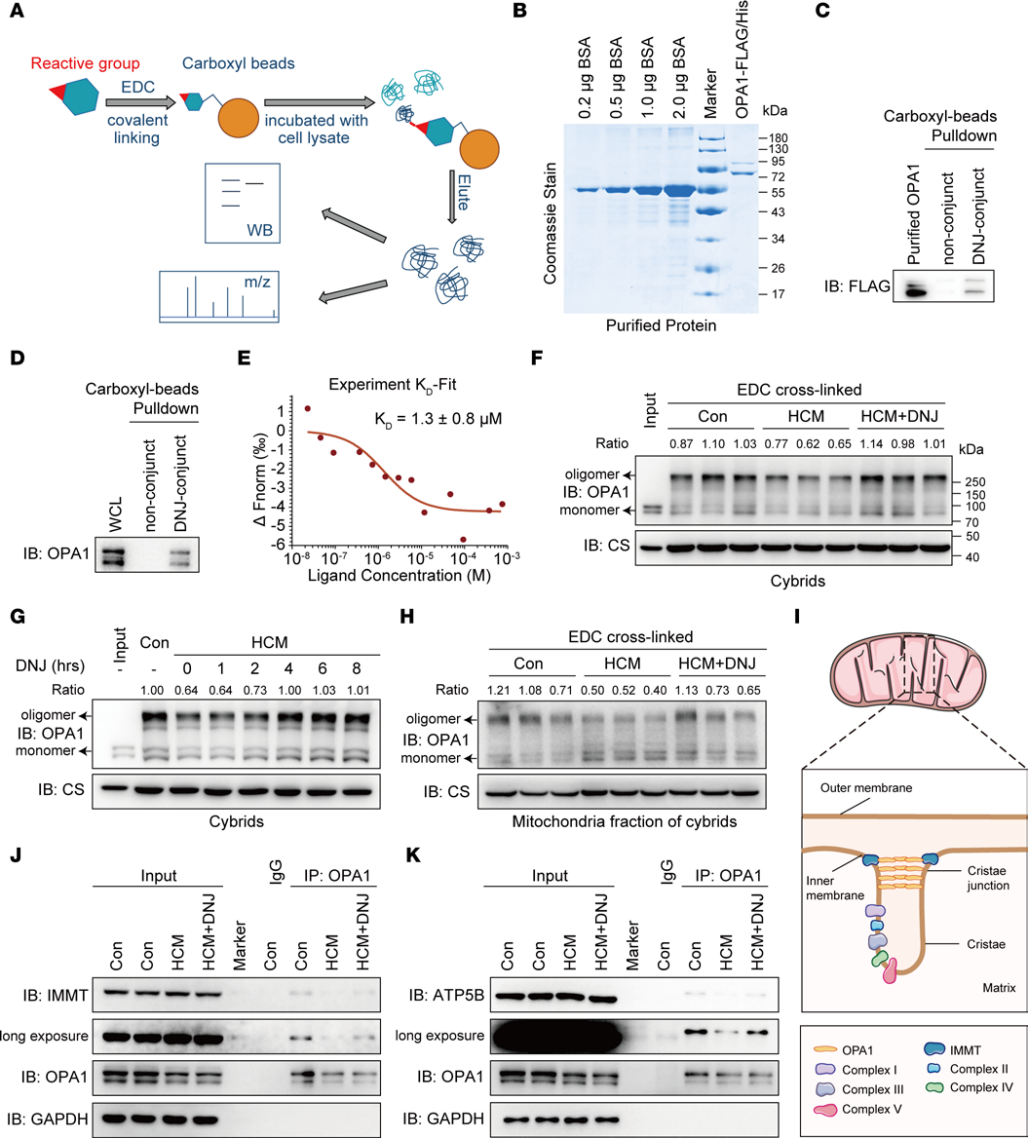
DNJ augments the level of OPA1 oligomers and improves its biomolecular function. (**A**) Schematic identifying the molecular target of DNJ combining pulldown and mass spectrum-proteomics analysis. (**B**) The Coomassie staining gel of eukaryotic purified Flag-His-OPA1 is shown. (**C**) Immunoblot confirmation of the DNJ-binding protein with purified Flag-His-OPA1. (**D**) Immunoblot confirmation of the endogenous DNJ-binding protein in cell lysis using OPA1 antibody. (**E**) MST assay for the affinity between DNJ and purified EGFP-OPA1 protein. (**F**) HCM cybrids were treated with DNJ for 8 hours and collected to purify the mitochondria. Mitochondria were incubated with 10mM EDC for 30 minutes. Proteins were separated by SDS-PAGE and immunoblotted using anti-OPA1 antibodies. (**G**) HCM cybrids treated with DNJ for different time periods (1, 2, 4, 6, and 8 hours). The above mitochondria were then treated as in (**F**). Proteins were separated by SDS-PAGE and immunoblotted using anti-OPA1 antibodies. (**H**) Mitochondria were isolated from cybrids and treated with DNJ or DMSO for 30 minutes. The above mitochondria were then treated as in (**F**). (**I**) Graphical illustration of OPA1 function in mitochondrial cristae remodeling. (**J** and **K**) Endogenous co-IP assay using IgG and OPA1 antibodies was performed to detect OPA1-IMMT (**J**) and OPA1-ATP5B (**K**) interactions.

To determine the molecular mechanism of DNJ in mitochondrial modulation, we performed a DNJ-conjugated bead pulldown assay. DNJ was immobilized by covalent conjugation to magnetic carboxyl beads and was subjected to a protein pulldown assay (Figure 5A). We first validated the availability of these DNJ-conjugated beads by immunoblotting of the ectopically expressed lysosomal α-glucosidase (GAA) protein, a previously reported binder of DNJ (Supplemental Figure 9A) (47). Through this approach, we incubated the DNJ-conjugated beads with the cybrid lysate and detected the captured prey proteins using a mass spectrometry–proteomics analysis (Supplemental Figure 9B and Supplemental Table 5). Compared with control beads, the DNJ beads were substantially enriched for a series of mitochondria-associated proteins (Supplemental Figure 9C). These candidates underwent pulldown-immunoblot verification, and OPA1 was identified as a potential target for DNJ (Supplemental Figure 9D). OPA1 is a master regulator of mitochondrial cristae formation. We further confirmed the physical interaction between DNJ and OPA1 using a DNJ-bead pulldown assay with purified recombinant OPA1 (Figure 5, B and C) and cell lysates (Figure 5D). Furthermore, we examined their interaction capacities using a microscale thermophoresis (MST) measurement with consistent results (K_D_ = 1.3 ± 0.8 μM) (Figure 5E), with EGFP as a negative control (Supplemental Figure 9E). All of these aspects suggest that DNJ could target OPA1 and potentially be involved in OPA1-related mitochondrial regulation.

### DNJ augments the level of OPA1 oligomers and improves their biomolecular function.

OPA1 can determine the mitochondrial cristae shape to regulate the assembly and stability of RCSs. Recent studies have revealed that the disassembly of OPA1 oligomers is linked to cristae remodeling (33, 35). To investigate whether DNJ benefited mitochondrial viability through the targeting of OPA1, we explored the molecular and functional alteration of OPA1 under DNJ treatment. While the oligomerized mitochondrial OPA1 was consistently decreased in the HCM cybrids, we found that DNJ treatment greatly augmented the level of mitochondrial OPA1 oligomers in the HCM cybrids (Figure 5F), while the basal level of OPA1 was also mildly affected (Supplemental Figure 9F). We next tested the time-dependent sensitivity of oligomeric mitochondrial OPA1 to DNJ. We found that the oligomerized OPA1 began to increase under the 2-hour treatment of DNJ and that the proenhanced oligomerization of OPA1 gradually terminated at approximately the 6-hour time point (Figure 5G). Furthermore, we confirmed the effect of DNJ on mitochondrial OPA1 in vitro. We applied DNJ to the isolated mitochondria of cybrids and observed that mitochondrial oligomeric OPA1 was upregulated as expected (Figure 5H). In addition to the homopolymer, the heteropolymer of OPA1 also contributes to the maintenance of mitochondrial cristae morphology. This is especially true of the polymer with the core mitochondrial contact site and cristae organizing system (MICOS) component IMMT and mitochondrial F_1_F_o_-ATP synthase (37, 48). The OPA1-MICOS interaction directed the CJ number and stability, and the OPA1-ATP synthase interaction could reverse respiratory chain inhibition (Figure 5I). The coimmunoprecipitation of endogenous OPA1 in cybrids showed that the interaction of OPA1 with IMMT (Figure 5J) or ATP5B (Figure 5K) was partially perturbed in the HCM cybrids, while treatment with DNJ robustly restored this pathologic dysregulation. Collectively, these results demonstrate that DNJ can target mitochondrial OPA1 to augment its oligomerization and improve its biomolecular function, also suggesting the potential positive function of DNJ in cristae shaping.

### DNJ protects the mitochondrial cristae morphology in HCM cybrids.

**Figure 6:**
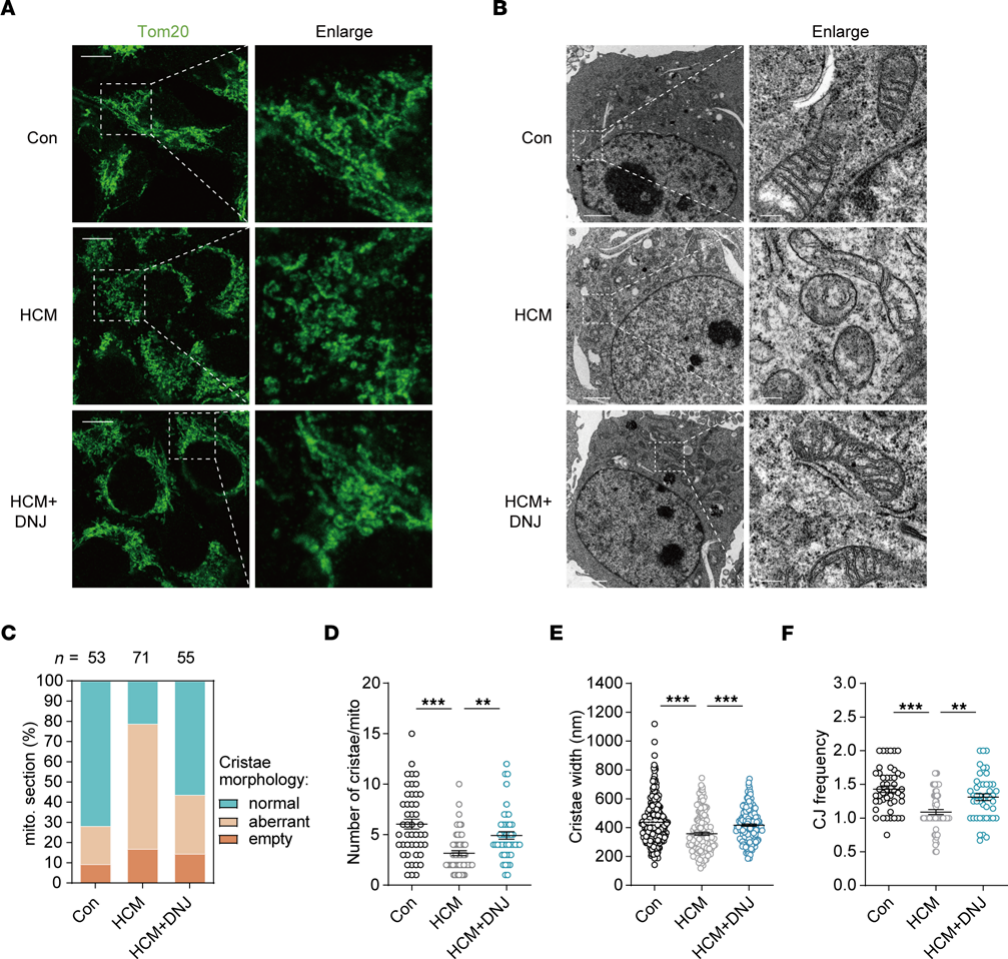
DNJ protects mitochondrial cristae morphology in HCM cybrids. (**A**) Mitochondrial networks of control cybrids, HCM cybrids and HCM cybrids with DNJ. Cybrids were immunolabeled for the mitochondrial marker TOM20. Scale bar, 20 μm. (**B**) Representative TEM recordings of cybrids. Scale bar for left images, 2 μm. Scale bar for enlarged images, 200 nm. (**C**) Quantification of the overall cristae morphology on TEM recordings. (**D**) Quantification of mitochondrial cristae number on TEM recordings. Con: *n* = 47, HCM: *n* = 61, HCM + DNJ: *n* = 45 biologically independent mitochondrion. Values represent the mean ± SEM. 1-way ANOVA followed by Tukey’s test. **P < 0.01, ***P < 0.001. (**E**) Diameter of cristae. Con: *n* = 241, HCM: *n* = 160, HCM + DNJ: *n* = 195 biologically independent mitochondrion. Values represent the mean ± SEM. 1-way ANOVA followed by Tukey’s test. ***P < 0.001. (**F**) CJ frequency on TEM recordings. The number of CJs was manually determined and normalized to the cristae number. Con: *n* = 47, HCM: *n* = 61, HCM + DNJ: *n* = 45 biologically independent mitochondrion. Values represent the mean ± SEM. 1-way ANOVA followed by Tukey’s test. **P < 0.01, ***P < 0.001.

To further examine the physiological manifestation of the molecular interaction of DNJ-OPA1, we sought to verify whether the DNJ-enhanced molecular benefits of OPA1 were linked to mitochondrial morphological and physiological improvement. Fluorescence and electron microscopy imaging of HCM cybrids showed that the mitochondria were fragmented with defects in the cristae structure (Figure 6, A–C). However, upon treatment with DNJ, the proportion of stable cristae and healthy tubular mitochondria increased. We further evaluated an additional 3 parameters: cristae number, cristae width, and the ratio between CJ and cristae number. Treatment with DNJ significantly increased the cristae number and CJ ratio of HCM cybrids, and the width of cristae was also markedly elongated in the DNJ-treated groups (Figure 6, D–F). These results indicate that the DNJ-enhanced OPA1 oligomers benefit mitochondrial cristae formation and shaping in HCM cybrids.

**Figure 7:**
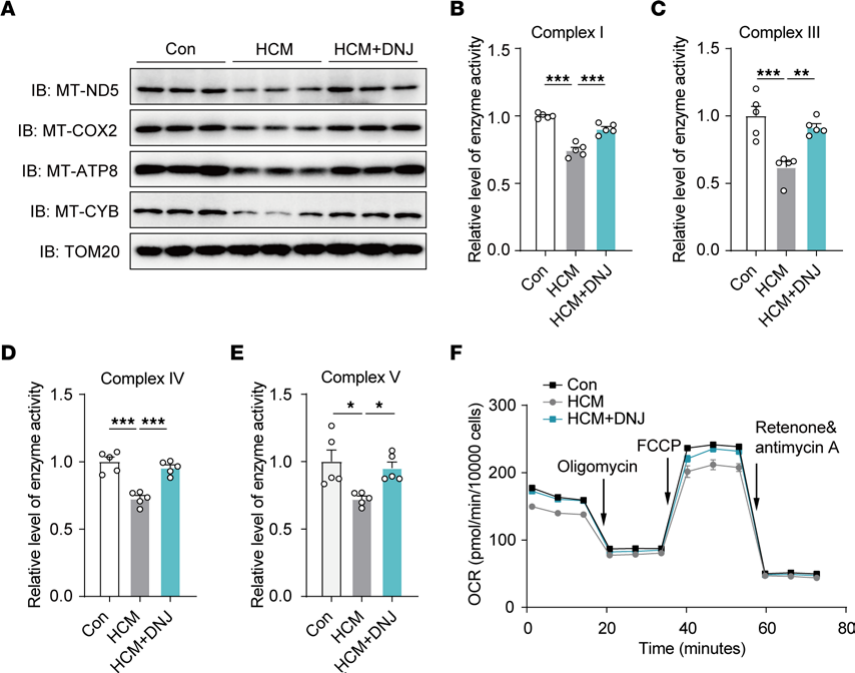
DNJ benefits mitochondrial function in HCM cybrids. (**A**) Western blotting and quantification analysis of respiratory electron transport chain complex subunits (MT-ND5, MT-COX2, MT-ATP8, and MT-CYB). TOM20 is shown as a loading control. (**B**–**E**) The activities of OXPHOS complexes were investigated using enzymatic assays on complex I, III, IV, and V. *n* = 5 biologically independent experiments. Values represent the mean ± SEM. 1-way ANOVA followed by Tukey’s test. *P < 0.05, **P < 0.01, ***P < 0.001. (**F**) Oxygen consumption rate (OCR) was measured using a seahorse analyzer. *n* = 3 biologically independent experiments. Data are representative of 3 independent experiments. Values represent the mean ± SEM.

Well-shaped mitochondrial cristae are indispensable for the stability and activity of RSCs (33). Considering that the patients from whom cybrids were created carried a mitochondrial ribosomal RNA mutation, we mainly focused on the biological performance of the mitochondria-encoded processes of RSCs. We found that the expression levels of mtDNA-encoded ETC complex subunits were aberrantly downregulated, presenting translational imperfections and partially impeding the assembly and stability of RSCs along with cristae in HCM pathological cybrids (Figure 7A). Conversely, applying DNJ effectively restored RSC stability, suggesting that the DNJ-induced improvement in cristae formation and shaping could provide a better-organized microdomain along the cristae for RSC assembly and stability. We then tested the physiological effect of DNJ on the activity of respiration complexes. The relative reaction activity of complexes I, III, IV, and V in the HCM cybrids represented a considerable collapse of these values compared with those of controls (Figure 7, B–E). However, applying DNJ markedly rescued the activity owing to the improved cristae and RSC stability. To further investigate the effect of DNJ on mitochondrial OXPHOS activity, the oxygen consumption rates (OCR) of cybrids were analyzed (Figure 7F). The basal OCR increased relative to that of the controls. These kinetic results showed that ATP-linked OCR and maximal OCR were increased by DNJ treatment, while spare capacity and proton leak were not significantly altered (Supplemental Figure 10B). Combining these results, we suggest that OPA1-targeting DNJ can benefit mitochondrial cristae modeling and thereby improve RSC stability and activity in a coordinated manner to compensate for mitochondrial rRNA mutation-induced mitochondrial disorders in HCM cybrids.

### DNJ sustains cristae structure and rescues mitochondrial dysfunction by increasing OPA1 oligomers in HCM iPSC-CMs.

**Figure 8:**
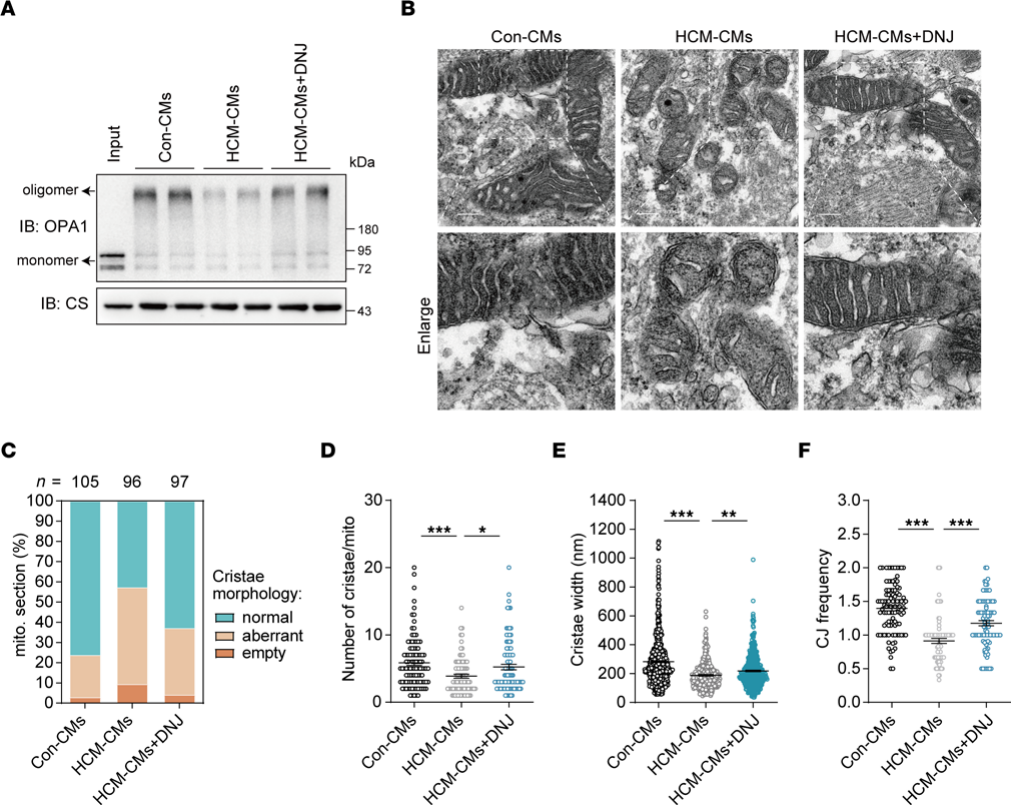
DNJ sustains cristae structure by increasing the OPA1 oligomers in HCM iPSC-CMs. (**A**) The mitochondria isolated from cardiomyocytes were incubated with crosslinker EDC as above. (**B**) Representative TEM recordings. Scale bar, 200 nm. (**C**) Quantification of the overall cristae morphology. (**D**–**F**) Quantification of mitochondrial cristae number (**D**), Diameter of cristae (**E**) and CJ frequency (**F**). Con: *n* = 101, HCM: *n* = 85, HCM + DNJ: *n* = 92 biologically independent mitochondrion for cristae number and CJ frequency. Con: *n* = 620, HCM: *n* = 332, HCM + DNJ: *n* = 467 biologically independent mitochondrial cristae for cristae diameter. Values represent the mean ± SEM. 1-way ANOVA followed by Tukey’s test. *P < 0.05, **P < 0.01, ***P < 0.001.

**Figure 9:**
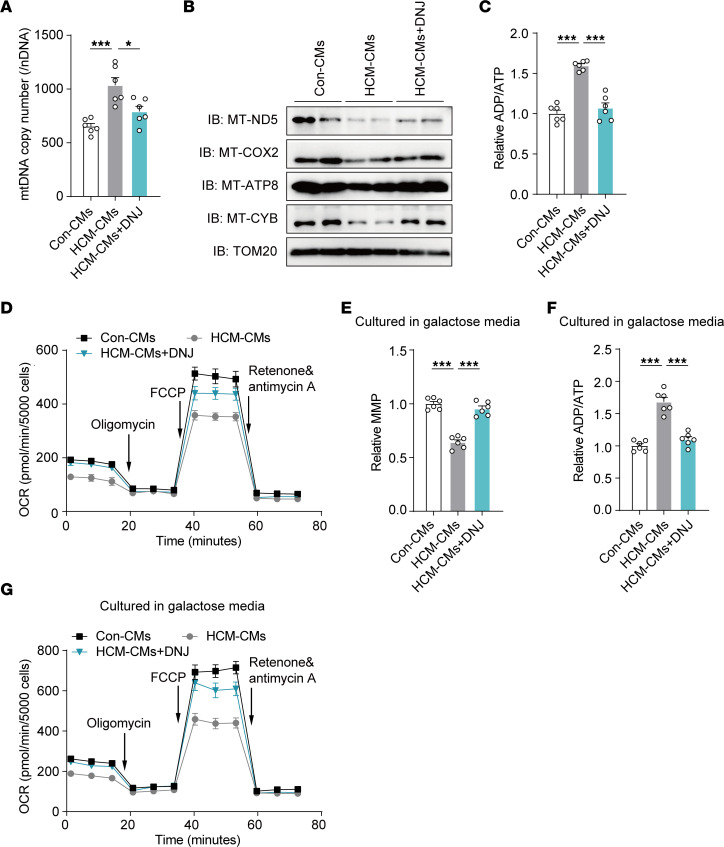
DNJ rescues the mitochondrial dysfunction in HCM iPSC-CMs. (**A**) Measurement of mtDNA copy number. *n* = 3 biologically independent experiments in 2 cell lines. Values represent the mean ± SEM. 1-way ANOVA followed by Tukey’s test. *P < 0.05, ***P < 0.001. (**B**) Western blotting of respiratory electron transport chain complex subunits. TOM20 is shown as a loading control. (**C**) Measurement of ADP/ATP. *n* = 3 biologically independent experiments in 2 cell lines. Values represent the mean ± SEM. 1-way ANOVA followed by Tukey’s test. ***P < 0.001. (**D**) Measurement of OCR. *n* = 3 biologically independent experiments. Data are representative of 3 independent experiments. Values represent the mean ± SEM. (**E** and **F**) MMP (**E**) and ADP/ATP (**F**) were measured in galactose medium. *n* = 3 biologically independent experiments in 2 cell lines. Values represent the mean ± SEM. 1-way ANOVA followed by Tukey’s test. ***P < 0.001. (**G**) OCR were measured in galactose media. *n* = 3 biologically independent experiments. Data are representative of 3 independent experiments. Values represent the mean ± SEM.

Patient-specific HCM iPSC-CMs retain the integrated genomic and pathological information of the patient, as demonstrated above. Compared with cybrids, cardiomyocytes have a higher demand for energy, which is mainly powered by mitochondria, and offer a system that is more closed to the physiological heart. Thus, we applied HCM iPSC-CMs to rigorously validate the physiological efficacy of DNJ. Mechanistically, we found that the decreased oligomeric OPA1 of HCM iPSC-CMs was greatly reversed by DNJ treatment (Figure 8A), which was consistent with the observation in cybrids (Figure 5F). In addition, mitochondrial cristae destruction was also observed in HCM iPSC-CMs by transmission electron microscopy imaging and morphometric analysis (Figure 8, B–F). HCM iPSC-CMs displayed more aberrant and empty mitochondria in comparison with control iPSC-CMs, and DNJ supplied remarkable rescue (Figure 8, B and C). The disruption of mitochondrial structure in indexes of cristae number, cristae width, and the ratio between CJ and cristae number showed an obvious amelioration in the DNJ-treated HCM iPSC-CMs, whereas no obvious morphological differences were noted by immunofluorescence (Figure 8, D–F, and Supplemental Figure 10A). The aberrant increase in mtDNA copy number in HCM iPSC-CMs was also alleviated by DNJ treatment (Figure 9A). Correspondingly, the mitochondrial RSC stability and kinetic activity of HCM iPSC-CMs were rescued upon DNJ treatment (Figure 9, B–G). DNJ reversed the collapse of ATP production in HCM iPSC-CMs (Figure 9C). This was also consistent with the findings in cybrids (Figure 1, D and E). The integral mitochondrial activity of HCM iPSC-CMs was robustly improved by DNJ, as evaluated by the metabolic analysis of OCR (Figure 9D and Supplemental Figure 10C). Consistently, we found that DNJ treatment significantly augmented the MMP (Figure 9E), ATP production (Figure 9F), and mitochondrial OXPHOS (Figure 9G and Supplemental Figure 10D) activity in HCM iPSC-CMs in galactose media, with mitochondria being the major source of cellular ATP. Collectively, these results strongly suggest that DNJ is effective in increasing oligomer levels of OPA1, rescuing mitochondrial function in HCM iPSC-CMs, and presents the potential for extended and broader clinical use of DNJ in other mitochondrial disease models.

### DNJ benefits mitochondrial function through OPA1.

**Figure 10:**
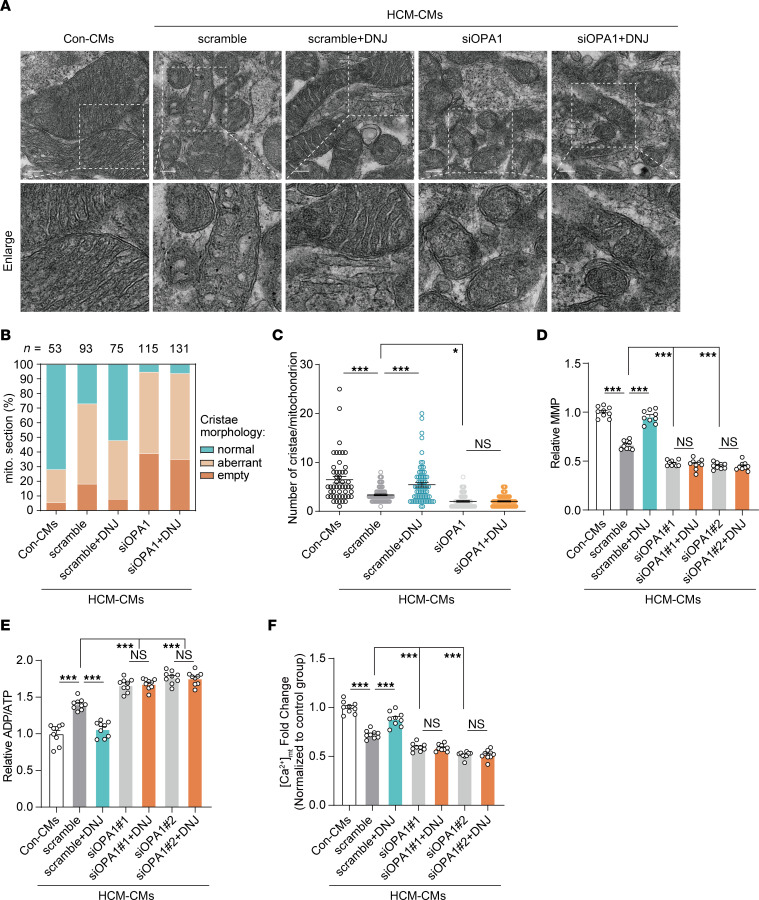
DNJ benefits mitochondrial function relying on OPA1 in HCM iPSC-CMs. (**A**) Representative TEM recordings of Con-CMs, HCM-CMs scramble, HCM-CMs scramble + DNJ, siOPA1 and siOPA1 + DNJ. Scale bar, 200 nm. (**B**) Quantification of the overall cristae morphology on TEM recordings. (**C**) Quantification of mitochondrial cristae number. Con: *n* = 50, HCM scramble: *n* = 76, HCM scramble + DNJ: *n* = 69, HCM siOPA1: *n* = 70, HCM siOPA1 + DNJ: *n* = 85 biologically independent mitochondrion. Values represent the mean ± SEM. 1-way ANOVA followed by Tukey’s test. *P < 0.05, ***P < 0.001. (**D**) Mitochondrial membrane potential was measured. *n* = 3 biologically independent experiments in 2 cell lines. Values represent the mean ± SEM. 1-way ANOVA followed by Tukey’s test. ***P < 0.001. (**E**) ADP/ATP ratio was measured using a bioluminescent assay system. *n* = 3 biologically independent experiments in 2 cell lines. Values represent the mean ± SEM. 1-way ANOVA followed by Tukey’s test. ***P < 0.001. (**F**) Analysis of mitochondrial calcium by RHOD-2 indicators in 7 groups. *n* = 3 biologically independent experiments in 2 cell lines. Values represent the mean ± SEM. 1-way ANOVA followed by Tukey’s test. ***P < 0.001.

To clarify whether the mitochondrial protective effect of DNJ relies on targeting OPA1, we tested physiological parameters upon DNJ treatment in OPA1-silenced cybrids and iPSC-CMs. We constructed OPA1-knockdown HCM cybrids (Supplemental Figure 11A) and iPSC-CMs (Supplemental Figure 12A) by specific small interfering RNA targeting OPA1 (siOPA1), and the knockdown efficiency was validated. Electron microscopy imaging and mitochondrial function assays showed that DNJ rescued the morphology and function of mitochondria in cybrids (Supplemental Figure 11, B–F) and HCM iPSC-CMs (Figure 10, A–E) but not in cells where siOPA1 had been applied. Moreover, we found that the benefits of DNJ in mitochondrial calcium buffering (Figure 10F) and cell calcium homeostasis (Supplemental Figure 13, A-G) were also impeded by loss of OPA1 in HCM iPSC-CMs.

GAA is the other binder of DNJ, as previously reported, and is involved in Pompe disease treatment (47). Therefore, we tested whether GAA was dependent on the potency of DNJ in mitochondrial HCM. We found that loss of GAA in cybrids (Supplemental Figure 11, A-C and G-I) and HCM iPSC-CMs (Supplemental Figure 12, B–G) did not have a marked impact on the ability of DNJ to rescue mitochondrial morphology, membrane potential and ATP production. Furthermore, we constructed HCM iPSC-CMs and cybrids with gradient levels of GAA to test the effect of DNJ. The results further confirmed that GAA had a mild effect on DNJ in improving mitochondrial viability (Supplemental Figure 11, J–L). Consistently, decreased GAA did not reverse the capability of DNJ in calcium regulation (Supplemental Figure 12H and Supplemental Figure 13, A–G). Collectively, these results suggest that DNJ protects mitochondrial HCM by targeting OPA1.

### DNJ improves mitochondrial bioenergetics in cardiac tissue and attenuates AngII-induced cardiac hypertrophy in mice.

**Figure 11:**
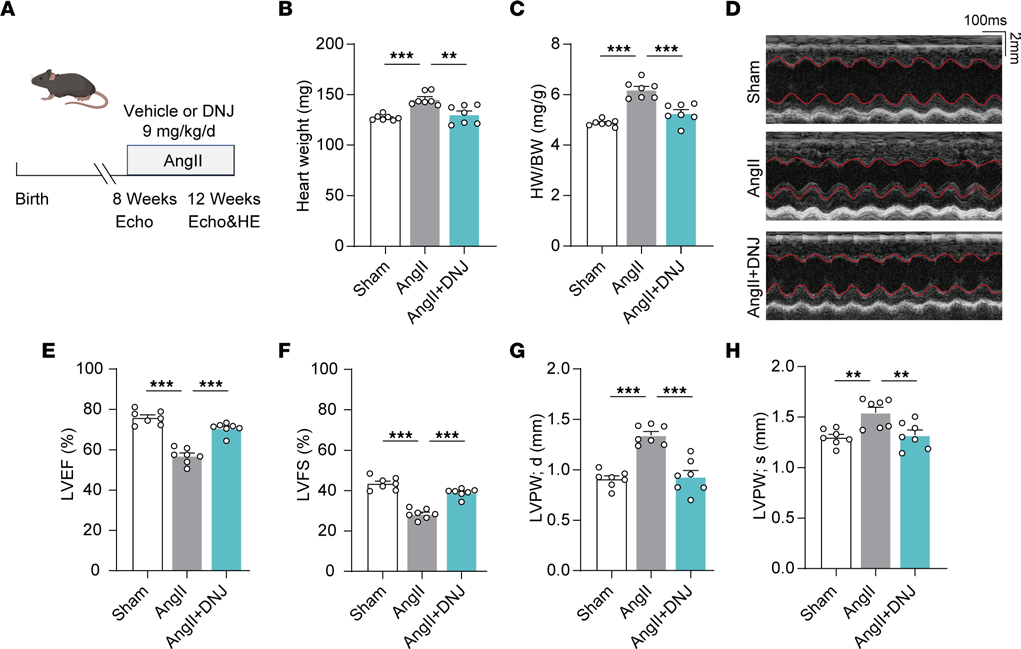
DNJ alleviates AngII-induced myocardial disfunction. (**A**) Schematic diagram depicting the experimental strategy for DNJ treatment. Echo: echocardiographic assessments, HE: hematoxylin-eosin staining. (**B** and **C**) Heart weight (HW) and heart weight normalized to body weight (BW). *n* = 7 mice. Values represent the mean ± SEM. 1-way ANOVA followed by Tukey’s test. **P < 0.01, ***P < 0.001. (**D**) Echocardiograms of sham, AngII, and DNJ group. (**E**–**H**) Echocardiography parameters (EF, FS, and LVPW). *n* = 7 mice. Values represent the mean ± SEM. 1-way ANOVA followed by Tukey’s test. **P < 0.01, ***P < 0.001.

**Figure 12:**
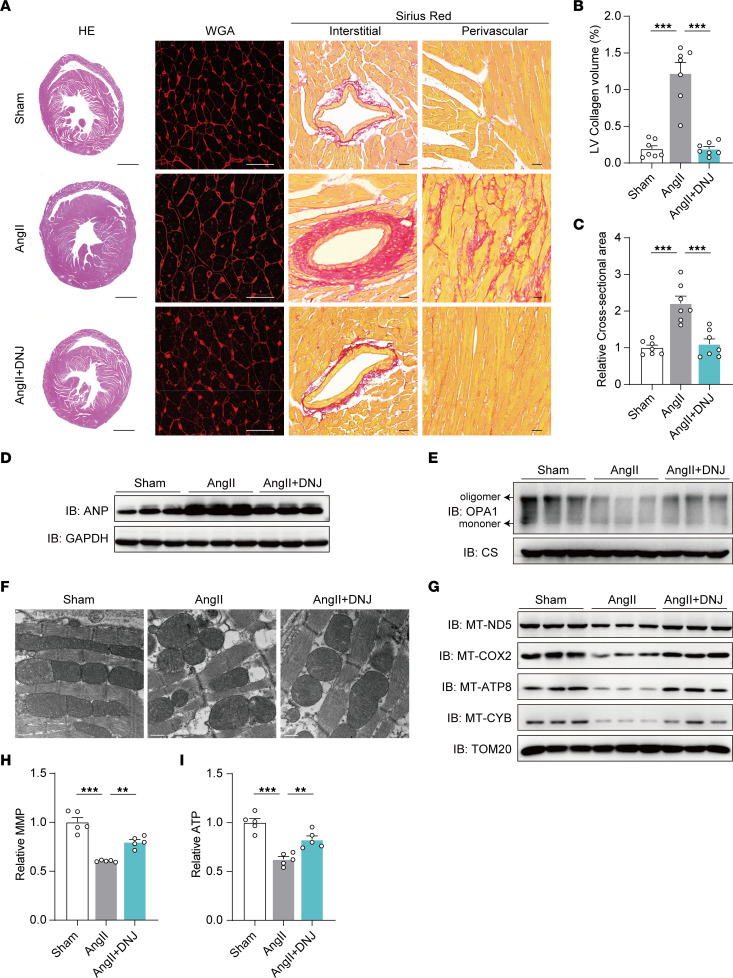
DNJ reverses AngII-induced mitochondrial dysfunction and cardiac hypertrophy. (**A**) Representative images of H&E staining, wheat germ agglutinin (WGA) staining (cardiac hypertrophy), and Picrosirius red staining (fibrosis). Scale bar, 1,000 μm (HE); 30 μm (WGA); 20 μm (Sirus Red). (**B**) LV collagen volume was assessed and quantified. *n* = 7 biologically independent samples of cardiac mouse tissues. Values represent the mean ± SEM. 1-way ANOVA followed by Tukey’s test. ***P < 0.001. (**C**) Quantitative results of average cross-sectional areas. *n* = 7 biologically independent samples of cardiac mouse tissues. Values represent the mean ± SEM. 1-way ANOVA followed by Tukey’s test. ***P < 0.001. (**D**) Immunoblotting ANP protein levels in cardiac tissues. (**E**) Western blotting of mitochondrial OPA1 with EDC treatment in cardiac tissues of Sham, AngII, and AngII + DNJ groups. (**F**) Representative TEM recordings. Scale bar, 500 μm. (**G**) Immunoblotting mitochondrial electron transport chain complex subunits expression. (**H** and **I**) Cardiac cells were isolated from the cardiac tissues of each mouse, separately. Relative MMP and ATP levels were then measured. *n* = 5 biologically independent samples of cardiac mouse tissues. 1-way ANOVA followed by Tukey’s test. **P < 0.01, ***P < 0.001.

We wanted to further validate the efficacy of DNJ against pathological myocardial hypertrophy in vivo. Given that each mammalian cell contains hundreds or even thousands of mitochondria and mitochondrial genome copies, there is still a technical limitation to edit the mitochondrial genes homogenetically at a single nucleotide pair level in mice. In this case, an angiotensin II (AngII)-induced cardiac hypertrophic mouse model can be used to clarify cardiovascular disease pathogenesis and evaluate relevant therapeutic strategies (49–51). Recent studies have indicated that AngII-mediated cardiac hypertrophy is closely correlated with mitochondrial dysfunction and that mitochondrial protection can attenuate AngII-induced heart failure (52–54). Therefore, we applied an AngII-induced cardiac hypertrophic mouse model to mimic cardiac mitochondrial dysfunction and cardiac hypertrophy (Figure 11A). After 4 weeks of treatment, the heart weight and ratio of heart weight to body weight of the AngII group were substantially higher than those of the control group, showing the effectiveness of this mouse model (Figure 11, B and C). In the cardiomyopathy mice treated with DNJ (9 mg/kg), the indexes of heart weight and heart weight/body weight were effectively rescued and decreased. We further performed echocardiographic assessments of left ventricular function and architecture, including left ventricular ejection fraction (LVEF), fraction shortening (LVFS), left ventricular end-diastolic diameter (LVPW; d), and left ventricular end-systolic diameter (LVPW; s). All the mice showed normal cardiac function at baseline (Supplemental Table 6). After injecting AngII, mice exhibited reduced myocardial function, which was markedly improved by DNJ treatment (Figure 11, D–H and Supplemental Table 7). Furthermore, morphological analysis revealed that AngII markedly increased heart size, while DNJ treatment rescued it (Figure 12A). At the histological level, we observed enlarged cardiomyocyte cross and increased collagen-positive area in the AngII group, while DNJ treatment protected the mice from AngII-induced cardiac hypertrophy and fibrosis (Figure 12, A–C). Consistent with the cardiac dysfunction in the AngII group, the expression of ANP and BNP, markers of hypertrophic cardiomyopathy, was upregulated, which was also alleviated by DNJ treatment (Figure 12D and Supplemental Figure 14, A and B). These results demonstrate the efficacy of DNJ in alleviating the manifestations of AngII-induced cardiac hypertrophy in mice without major side effects.

To investigate whether DNJ improved cardiac dysfunction by rescuing mitochondrial function, we examined the mitochondrial ultrastructure and bioenergetics of cardiac tissues in these 3 groups. We found that AngII-treated mice presented mitochondrial dysfunction and abnormal mitochondrial morphology compared with control mice, while DNJ treatment markedly decreased AngII-induced cristae disorganization (Figure 12F) and restored RSC stability (Figure 12G), MMP levels (Figure 12H), and ATP production (Figure 12I) with augmented OPA1 oligomer levels (Figure 12E). In contrast, the Sham+DNJ mice group exhibited few changes (Supplemental Figure 15, A–N and Supplemental Tables 8 and 9). Furthermore, abnormalities in the other organs — liver, stomach, colon, kidney, and spleen — were not observed in the DNJ-treated groups (Supplemental Figure 16). Taken together, these results further suggest that DNJ can attenuate AngII-induced cardiac hypertrophy by improving mitochondrial function in mice, highlighting the therapeutic potential of DNJ for our patients with HCM and, potentially, other types of hypertrophic cardiomyopathy.

## Discussion

**Figure 13:**
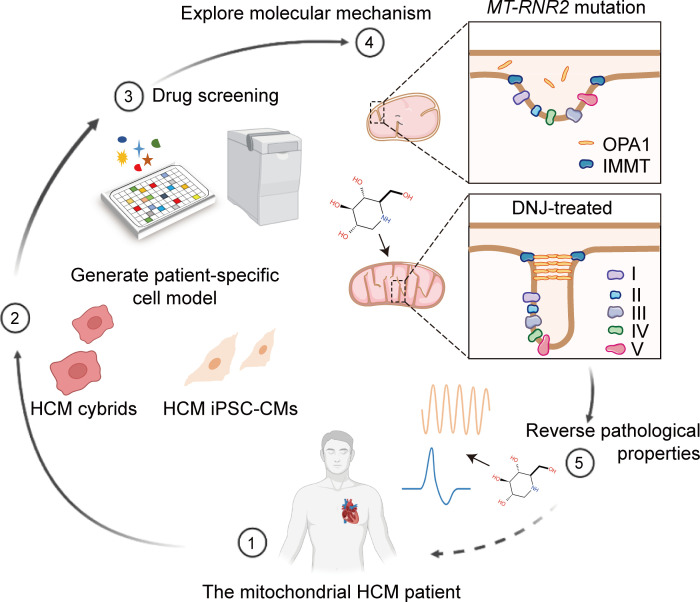
Schematic of the mechanism underlying mitochondria-targeted DNJ rescuing HCM.

In this study, we developed a 2-step drug screening platform by utilizing 2 cell models, HCM cybrids and HCM iPSC-CMs. Our study identified DNJ from the library of mitochondria-associated compounds and found that it could be a potent agent for the rescue of mitochondrial function in our mitochondrial HCM. The angiotensin II–induced cardiac hypertrophy mouse model further verified the efficacy of DNJ in promoting cardiac mitochondrial function and alleviating cardiac hypertrophy in vivo. These findings highlight the potential medicinal role of drug candidates in the treatment of mitochondrial diseases (Figure 13).

Given that each mammalian cell contains hundreds or even thousands of mitochondria, manipulating the mitochondrial genes at a single nucleotide pair level in mouse or cell models is a technique that still faces considerable challenges. The efforts of pathological and pharmacological mimicry of mitochondrial diseases such as HCM in mice have encountered hurdles. In this case, the easy-to-culture patient-specific cybrids provide a convenient approach for primary drug screening, and the patient-specific iPSC-CMs provide advanced accurate mimicry of the patient’s pathology for further drug validation. Ongoing cardiotoxicity assessments using iPSC-CMs have already become an essential process of the comprehensive in vitro proarrhythmia Assay (CiPA) (55). The current chemical screens for mitochondrial diseases use one model and show low efficiency and/or high cost. Combining patient-specific-cybrids and iPSC-CM models could be a potent drug-screening strategy for mitochondrial diseases. Our identification of DNJ via the 2-step drug screening process detailed here could provide a paradigm for personalized drug discovery in other mitochondrial diseases.

Angiotensin II is a key member of the renin-angiotensin system and plays a vital role in hypertension and left ventricular hypertrophy. Applying angiotensin II–induced pathological cardiac hypertrophic mouse model is a common strategy for exploring HCM pathogenesis (49–51). Previous studies reported that the AngII-mediated cardiac hypertrophic model was accompanied by mtDNA deletions, impaired mitochondrial ultrastructure, and defective mitochondrial biogenesis (52–54). Here, we observed abnormal mitochondrial energy metabolism in AngII-treated mice. Treatment with DNJ, a mitochondrial rescue agent, improved mitochondrial function to an obvious degree and further alleviated the manifestations of AngII-induced cardiac hypertrophy in mice. Combining these results, we verified the efficacy of DNJ in mitochondrial protection. In addition, DNJ prevented AngII damage in heart failure, providing a potential therapeutic strategy for cardiac hypertrophy.

DNJ is a type of polyhydroxy alkaloid and is one of the major active components of mulberry leaves. DNJ has been previously reported as an α-glucosidase inhibitor (56), and some of its derivatives have also been found to alleviate heart failure in other indirect ways (57–59). Although this GAA-associated side effect of DNJ is, for the most part, excluded from this study, considering the complicated regulatory network of energy metabolism in vivo, these reports suggest that DNJ may counteract HCM through other indirect pathways in addition to its main effect of targeting mitochondria. DNJ has also been reported to have some effects on obesity-induced lipid abnormalities and mitochondrial dysfunction (60). These results may suggest the potential application of DNJ in other mitochondria-associated diseases. Interestingly, DNJ has incidentally been found to exhibit cardioprotective properties in patients, although thorough experimental evidence was undefined in this study (61). Here, we identified the role of DNJ in mitochondrial hypertrophic cardiomyopathy and provided insight into DNJ-rescued cardiomyopathy. Notably, a phase III clinical trial of DNJ for Pompe disease has been conducted (NCT03729362). DNJ showed clinical safety, and this could be a valuable reference for drug use in possible clinical trials in the future. In addition, DNJ can be used as a blueprint for the development of more effective derivatives is reliable and promising.

OPA1 is a master regulator of mitochondrial cristae. It is active in facilitating the assembly of respiratory chain proteins and in their maintenance. Oligomerized OPA1 governs cristae morphology and is independent of OPA1’s role in mitochondrial fusion (32, 36, 37). The balance between OPA1 monomers and oligomers is crucial for cristae remodeling and influences disease progression (35, 62). OPA1 has been considered a promising target for the treatment of mitochondrial diseases (63). However, the process of discovering chemical activators for OPA1 has been patchy. Our investigation reveals that DNJ physically interacts with OPA1 and physiologically reverses the mitochondrial dysfunction caused by *MT-RNR2* mutation. This mutation impairs translation capacity and thereby decreases respiratory chain proteins, leading to downregulated MMP and ATP production (40–42). The DNJ-increased OPA1 oligomer sustained the cristae shape and promoted both RCS assembly and respiratory capacity in HCM cell lines. Therefore, regulating OPA1-mastered cristae remodeling provides insight into counteracting mitochondrial dysfunction.

Although mitochondria have their own genome, the vast majority of mitochondria-related proteins are nuclear encoded. As nuclear-modifier genes could functionally influence the clinical manifestation caused by mtDNA mutations, the search for small molecules to target such nuclear-encoded proteins is an essential component in mitochondrial disease treatment (7, 64–66). Consequently, bezafibrate, an agonist of peroxisome proliferator–activated receptor, was found to remarkably delay mtDNA deletion accumulation in a mouse model with a mtDNA helicase mutation (67). In addition, high-throughput chemical and CRISPR screens have been used to identify I-BET525762A, an inhibitor of bromodomain-containing protein 4, in resolving complex I defect (mt.3796A>G) cybrids with Leigh’s syndrome (68). Our findings presented medical insight for treatment of mitochondrial disease**.** We found that DNJ targeted nuclear-encoded OPA1 to function as a mitochondrial rescue agent, showing the therapeutic potential of our mitochondrial hypertrophic cardiomyopathy and even other mitochondrial diseases, such as hearing loss, mitochondrial myopathy, or Leigh syndrome.

The limitation of this study should also be mentioned. Although iPSC-CMs can substantially phenocopy somatic CMs and were regarded as a potent cell model for the study of mechanisms and drug discovery, they are not as mature as human adult cardiomyocytes. Developing models like organoids, 3D-engineered tissues, and mitochondrial gene-edited mouse models may be advantageous in future studies (69, 70).

In summary, we identified DNJ for its potential to promote the mitochondrial health and reverse the pathological phenotype of mitochondrial hypertrophic cardiomyopathy via a 2-step drug screening, which presents a convenient preclinical platform for personalized treatment in mitochondrial diseases.

## Methods

### Cell culture and treatment of cybrids and iPSCs.

Cybrids were grown in high-glucose DMEM (GIBCO) supplemented with 10% FBS (Gibco), 1% penicillin-streptomycin (100 U/mL) (Gibco) at 37°C and 5% CO2 (v/v). iPSCs were cultured in mTESR1 (STEMCELL Technologies) media on Matrigel-coated plates (Corning). For compound treatment, cybrids were allowed to adhere for 16 hours before treatment with 30 μM DNJ for 24 hours.

### Differentiation of iPSCs into cardiomyocytes.

Cardiomyocyte differentiation was induced using monolayer myocardial differentiation protocols as previously described (28), with minor modifications. Cells were treated with 12 μM CHIR99021 (SELLECK) for 1 day in RPMI (GIBCO) and B27 supplement minus insulin (GIBCO) (RPMI + B27-Insulin) until cells were expanded to 90% cell confluence and then replaced by RPMI+B27-Insulin. After 2 days, cells were treated with 5 μM IWP2 (TOCRIS). IWP2 was removed on day 6. From day 8, cells were cultured in RPMI + B27. Spontaneously contracting cells could be observed from day 10 to 14. Cells were replated for purification on day 15.

### Antibodies.

Specific antibodies were purchased from the following commercial sources for the indicated experiments: anti-CS (ab129095, 1:2,000 for immunoblotting (IB)), anti-IMMT (ab137057, 1:1,000 or IB), anti-ANP (ab191398, 1:1,000 for IB, 1:50 for immunofluorescence), anti-BNP (ab92500, 1:10,00 for IB), anti-NFATC4 (ab62613, 1:1,000 for IB, 1:50 for IF), anti-MLC2v (ab92721, 1:50 for IF), goat anti-rabbit IgG H&L (Alexa Fluor 594) (150080, 1:250 for IF), goat anti-mouse IgG H&L (Alexa Fluor 488) (150113, 1:250 for IF) from Abcam. Additionally, anti-Tom20 (42406, 1:2,000 for IB, 1:50 for IF), anti-Vinculin (13901, 1:2,000 for IB), anti-OPA1 (67589S, 1:1,000 for IB, 1:50 for IP) from Cell Signaling Technology; anti-α-actinin (A7811, 1:200 for IF) were purchased from Sigma. From Abclonal, we purchased anti-ATP5B (A5769, 1:1,000 for IB), and anti-MLC2a (17283-1-AP, 1:50 for IF) from Proteintech. Anti-DYKDDDDK-tag (M20008, 1:10,000 for IB), anti-GAPDH (M20050, 1:5,000 for IB) were purchased from Abmart, and HRP goat anti-mouse IgG (H+L) (BK-R050, 1:5,000 for IB) and HRP goat anti-rabbit IgG (H+L) (BK-M050, 1:5,000 for IB) were purchased from Bioker.

### Immunofluorescence.

Cells were seeded in glass slides for cybrids and iPSC-CMs. The slides were fixed with 4% formaldehyde for 15 minutes, then permeabilization was conducted with 0.2% Triton X-100 for 15 minutes and blocked by 3% BSA for 1 hour at room temperature (RT). The cells were incubated with primary antibody overnight at 4°C. Secondary antibody was incubated for 1 hour, and DAPI was incubated for 10 minutes at RT.

### Cloning procedures and cell transfection.

The full-length OPA1 was cloned from HEK293T cDNA by PCR. A GAA full-length template was gifted from the Jia-huai Han lab. The above genes were cloned into pcDNA3.1-Flag/His empty vectors.

Plasmids were transfected by Lipofectamine 3000 (Life Technologies) in HEK293T or cybrids. The culture was changed to DMEM and 10% FBS after 8 hours. And the cells were harvested after 36 hours.

### Protein recombination and purification.

Recombinant proteins for Flag/His-OPA1 were purified from overexpression vectors transduced HEK293T cells. FLAG (M2) magnetic beads (Sigma) were used to enrich proteins and 3 × FLAG peptide (Sigma) was applied to elute. The purity of recombinant protein was measured with the standard BSA control by SDS-PAGE and Coomassie staining.

### Mitochondrial purification.

Cells (at least 3 10 cm^2^ dishes) were suspended by KPBS (136 mM KCl, 10 mM KH2PO4, pH 7.25) containing protease inhibitor (Roche) and then transferred to dounce homogenization. (71). After 50 strokes, the cell extraction was centrifuged at 4°C at 1,000*g* for 10 minutes. The supernatant was further centrifuged at 13,500*g* for 5 minutes. After 3 washes, the resulting pellet (mitochondria) was collected by centrifugation at 4°C at 13,500*g* for 5 minutes.

The mitochondria from heart tissues were extracted following manufacturers’ instruction by Tissue Mitochondria Isolation Kit (Beyotime).

### Protein crosslinking.

For protein crosslinking, mitochondria were treated with 10 mM EDC (Sangon Biotech) for 30 minutes at 37°C. We added 15 mM DTT (Sangon Biotech) to the sample buffer for quenching the crosslinking reaction. The mitochondrial pellets were harvested by centrifugation for 5 minutes at 12,000*g* at 4°C.

### Cell Lysis, immunoprecipitation and immunoblotting.

Cells were resuspended in lysis buffer (Fude Biological Technology) with a complete protease inhibitor cocktail. Supernatants were obtained by centrifugation at 13,000*g* for 15 minutes at 4°C and further applied for IB or immunoprecipitation (IP) with the indicated antibodies. For IP, control IgGs and the corresponding primary antibodies were added to the prepared lysates for 2 hours at 4°C, and then protein A/G agarose beads (Santa Cruz) were added to the lysates. After 2 hours, the beads were washed with NETN buffer (25 mM Tis-HCl pH 8.0, 100 mM NaCl, 1 mM EDTA, 0.5 mM DTT) 3 times for 3 minutes at 4°C. The protein eluted from the beads with 50 μL 2 × SDS loading buffer could be detected by IB.

### Pull down and mass spectrometry analysis.

Whole cell lysates were prepared by lysis buffer. DNJ-conjugated carboxyl beads were prepared according to manufacturer’s instructions (PuriMag Biotech). Cell lysates were then incubated with beads at 4°C for 4 hours. After washing 3 times each for 3 minutes with lysis buffer, the beads were boiled in 50 μL 2 × SDS loading buffer for 10 minutes. The supernatant was analyzed via SDS-PAGE or LC/MS.

### Galactose induced cell death assay.

For the galactose assay, iPSC-CMs were seeded in 12-well plates grown in high-glucose RPMI + B27. After 16 hours, cells were washed twice and the media was changed into RPMI without glucose, but supplemented with 10 mM galactose, 4 mM glutamine and B27. Cells were incubated in galactose media with DMSO or DNJ and then trypsinized and quantified each day.

### Measurement of cellular respiration.

Oxygen consumption of mitochondria was assessed using a Seahorse XFe96 Analyzer. After adhering for 16 hours, cells were incubated in the media with DMSO or DNJ for another 24 hours. After baseline records, cells were then injected with 1 μM oligomycin, 1 μM FCCP, and 0.5 μM of rotenone with 0.5 μM of antimycin A, in that order.

### MMP and ATP measurement.

The MMP was measured by fluorescence detection (JC-10 Assay Kit, Abcam) following manufacturer’s instruction. The ATP production ability and the results of the ADP/ATP ratio were measured using an ATP Assay Kit (Beyotime) and ADP/ATP Ratio Assay Kit (Sigma) separately, following manufacturers’ instruction.

### Measurement of activities of respiratory complexes.

The enzymatic activities of complexes I, III, IV, and V were assayed by changed absorbance (72). The activities of complexes were measured using the following parameters: complex I through the oxidation of NADH; complex III through the reduction of cytochrome c; complex IV through the oxidation of cytochrome c; and complex V through NADH oxidation.

### Transmission electron microscopy.

Mitochondria were fixed at 4°C using 2.5% glutaraldehyde overnight. Thin sections were imaged on Tecnai G2 Spirit 120kV in the Center of Cryo-Electron Microscopy (CCEM), Zhejiang University.

### Patch clamping.

iPSC-CMs were enzymatically dissociated into single cells and seeded to matrigel-coated glass coverslips. Spontaneous cells were selected for further recording using an EPC-10 patch clamp amplifier (HEKA) (73). The PatchMaster software (HEKA) was used for data acquisition. The IgorPro (Wavemetrics) and Prism (Graphpad) were applied for data analysis.

### Detection of calcium signals.

For cytosolic calcium detection, cells were collected and stained with Frua-2 AM (5 μM; Invitrogen). And cytosolic calcium signals were detected using Ultra High Speed Wavelength Switcher ((Lambda DG-4, Sutter instruments) with a CCD camera (Zyla, Andor) mounted on an inverted microscope (Eclipse Ti, Nikon Instruments Inc.). Data were acquired using NIS-Elements software (Nikon Instruments Inc.).

For mitochondrial calcium detection, mito-GECO1 were transfected into cells or RHOD-2 (2.5 μM) in Hank’s balanced salt solution (Gibco) were stained for cells and the data acquired by Microplate reader or Fluorescence microscope.

### Quantitative real-time PCR (qPCR).

For the qPCR assay, total mRNA was extracted from mouse heart tissue using M5 Universal RNA Mini Kit (Mei5bio). RNA was reverse-transcribed into cDNA using PrimeScript RT reagent Kit with gDNA Eraser (Takara). Bestar SybrGreen qPCR Mastermix (DBI) was used to quantify the expression of BNP and ANP. The primers for qPCR were: *Bnp* (F: GTGACGTTGACATCCGTAAAGA; R: GCCGGACTCATCGTACTCC); *Anp* (F: TACCCGCCATCCATGATCG; R: AGGCAGTCCACTTCAGTGC); and *Gapdh* (F: ATGTGTCCGTCGTGGATCTG; R: AGTTGGGATAGGGCCTCTCTT).

### OPA1 and GAA silencing.

Cells were transfected by Lipofectamine RNAiMAX regent (Thermo Fisher Scientific) following the manufacturer’s instructions. All siRNA sequences were designed according to the siRNA Selection Program (http://sirna.wi.mit.edu/home.php/) (74) and commercially produced (GenePharma).

The sequence (5′–3′) of siRNA against OPA1 was: si#1: CCAUGUGGCCCUAUUUAAA; si#2: CCAAGUGACUACAAGAAAU. The sequence (5′–3′) of siRNA against GAA was si#1: GGACUUGGGAGAUUCUAAA; si#2: CAGAAAUCCUGCAGUUUAA.

### Animals and treatment.

The mice used in this research were 8-week-old male mice on a C57BL/6J background. The experimental groups (*n* = 7 per group) contained (a) PBS; (b) PBS + DNJ (9 mg/kg/day); (c) AngII (4.5 mg/kg/day); and (d) AngII + DNJ (54). The above regents were given by i.p. injection twice a day for 28 days. The mice were killed using CO_2_, and their hearts and other organs were immediately removed.

### Echocardiography measurements.

Mice were anesthetized, and 2-dimensional (2D) parasternal short axis M-mode echocardiograms were performed using the vinno 6LAB. M-mode tracings at mid-papillary muscle level were recorded to assess left ventricular function.

### Histological analysis.

For histological assay, mice hearts were collected and fixed with 4 % paraformaldehyde. The images were obtained from sections stained with H&E staining, wheat germ agglutinin (WGA) staining for cardiac hypertrophy, and Picrosirius red staining for fibrosis, and the cross-sectional areas and fibrotic areas of cardiomyocytes were evaluated.

### Statistics.

Statistical analyses are displayed using Student’s unpaired, 2-tailed *t* test in Prism (Graphpad) to compare 2 groups. 1-way ANOVA or 2-way ANOVA were used to compare more than 2 groups. All data represent 3 mutant (2 patients) and 3 control (2 control individuals) in cybrid cell lines. In iPSCs, 2 mutant clones are derived from the same patient; 2 control clones are from the proband’s son and a genetically unrelated individual in the same region, separately. *n* is the total replicates for each group. Values represent the mean ± SEM of at least 3 independent experiments. *P* < 0.05 was considered significant.

### Study approval.

All animal experiments were performed with approval from and according to the protocols of the Committee of Animal Ethics of Zhejiang University.

## Author contributions

QY initiated and supervised the project. QY and QZ designed the research. QZ, FG, and LF performed most of the biochemical and molecular experiments with assistance from YD, SX, XD, SH, XDZ, YJ, HZ, YQ, ZL, ML, and HC. MF, LS, YF, DZ, AL, XL, TK, RZ, PL, and ZL contributed to discussion and data interpretation. TK, RZ, PL and ZL edited the manuscript. QY and QZ wrote the manuscript.

## Supplementary Material

Supplemental table 5

Supplemental data

## Version 1 05/18/2023

In-Press Preview

## Version 2 07/17/2023

Electronic publication
