# Supplementary material for: 1-Deoxynojirimycin promotes cardiac function and rescues mitochondrial cristae in mitochondrial hypertrophic cardiomyopathy
Source: J Clin Invest. 2023 Jul 17;133(14):e164660. doi: 10.1172/JCI164660 (PMC10348775; doi:10.1172/JCI164660)
Supplement: Supplemental table 5 [file jci-133-164660-s047.pdf]

Table S5. Protein identification results for DNJ pulldown assay

PSMs (DNJ) / PSMs (control) ≥ 2.0

Uniprot ID	Description	DNJ PSMs	DNJ unique peptides	Con PSMs	Con unique peptides
Q5T9A4	ATPase family AAA domain-containing protein 3B OS=Homo sapiens OX=9606 GN=ATAD3B PE=1 SV=1	346	13	170	13
Q5T2N8	ATPase family AAA domain-containing protein 3C OS=Homo sapiens OX=9606 GN=ATAD3C PE=2 SV=2	190	3	0	0
P11940	Polyadenylate-binding protein 1 OS=Homo sapiens OX=9606 GN=PABPC1 PE=1 SV=2	123	25	17	12
Q9BVA1	Tubulin beta-2B chain OS=Homo sapiens OX=9606 GN=TUBB2B PE=1 SV=1	118	3	0	0
O60313	Dynamin-like 120 kDa protein, mitochondrial OS=Homo sapiens OX=9606 GN=OPA1 PE=1 SV=3	108	47	24	16
Q16891	MICOS complex subunit MIC60 OS=Homo sapiens OX=9606 GN=IMMT PE=1 SV=1	105	44	34	24
Q13310	Polyadenylate-binding protein 4 OS=Homo sapiens OX=9606 GN=PABPC4 PE=1 SV=1	101	23	17	11
Q13509	Tubulin beta-3 chain OS=Homo sapiens OX=9606 GN=TUBB3 PE=1 SV=2	101	8	47	2
Q6S8J3	POTE ankyrin domain family member E OS=Homo sapiens OX=9606 GN=POTEE PE=2 SV=3	98	3	0	0
P10809	60 kDa heat shock protein, mitochondrial OS=Homo sapiens OX=9606 GN=HSPD1 PE=1 SV=2	94	34	46	22
P42704	Leucine-rich PPR motif-containing protein, mitochondrial OS=Homo sapiens OX=9606 GN=LRPPRC PE=1 SV=3	94	53	41	33
Q92900	Regulator of nonsense transcripts 1 OS=Homo sapiens OX=9606 GN=UPF1 PE=1 SV=2	91	43	9	9
P68363	Tubulin alpha-1B chain OS=Homo sapiens OX=9606 GN=TUBA1B PE=1 SV=1	89	9	41	12
Q14157	Ubiquitin-associated protein 2-like OS=Homo sapiens OX=9606 GN=UBAP2L PE=1 SV=2	87	29	2	2
Q6NUK1	Calcium-binding mitochondrial carrier protein SCaMC-1 OS=Homo sapiens OX=9606 GN=SLC25A24 PE=1 SV=2	85	27	4	4
P35908	Keratin, type II cytoskeletal 2 epidermal OS=Homo sapiens OX=9606 GN=KRT2 PE=1 SV=2	82	28	41	15
P13645	Keratin, type I cytoskeletal 10 OS=Homo sapiens OX=9606 GN=KRT10 PE=1 SV=6	74	25	32	14
Q7L2E3	Putative ATP-dependent RNA helicase DHX30 OS=Homo sapiens OX=9606 GN=DHX30 PE=1 SV=1	72	43	13	13
P07814	Bifunctional glutamate/proline--tRNA ligase OS=Homo sapiens OX=9606 GN=EPRS PE=1 SV=5	70	48	7	7
P51114	Fragile X mental retardation syndrome-related protein 1 OS=Homo sapiens OX=9606 GN=FXR1 PE=1 SV=3	67	23	8	6
Q92945	Far upstream element-binding protein 2 OS=Homo sapiens OX=9606 GN=KHSRP PE=1 SV=4	66	27	3	3
Q9H5Q4	Dimethyladenosine transferase 2, mitochondrial OS=Homo sapiens OX=9606 GN=TFB2M PE=1 SV=1	63	22	26	16
P68366	Tubulin alpha-4A chain OS=Homo sapiens OX=9606 GN=TUBA4A PE=1 SV=1	59	1	0	0
P06576	ATP synthase subunit beta, mitochondrial OS=Homo sapiens OX=9606 GN=ATP5F1B PE=1 SV=3	58	22	7	7
Q9BQG0	Myb-binding protein 1A OS=Homo sapiens OX=9606 GN=MYBBP1A PE=1 SV=2	58	38	24	17
O95573	Long-chain-fatty-acid--CoA ligase 3 OS=Homo sapiens OX=9606 GN=ACSL3 PE=1 SV=3	55	22	24	10
Q9NZ01	Very-long-chain enoyl-CoA reductase OS=Homo sapiens OX=9606 GN=TECR PE=1 SV=1	55	16	19	11
Q3ZCM7	Tubulin beta-8 chain OS=Homo sapiens OX=9606 GN=TUBB8 PE=1 SV=2	54	1	0	0
P25705	ATP synthase subunit alpha, mitochondrial OS=Homo sapiens OX=9606 GN=ATP5F1A PE=1 SV=1	52	27	23	16
P49368	T-complex protein 1 subunit gamma OS=Homo sapiens OX=9606 GN=CCT3 PE=1 SV=4	51	25	17	13
Q06787	Synaptic functional regulator FMR1 OS=Homo sapiens OX=9606 GN=FMR1 PE=1 SV=1	45	22	4	4
Q14671	Pumilio homolog 1 OS=Homo sapiens OX=9606 GN=PUM1 PE=1 SV=3	45	23	8	8
Q6Y7W6	GRB10-interacting GYF protein 2 OS=Homo sapiens OX=9606 GN=GIGYF2 PE=1 SV=1	44	34	15	9
Q15070	Mitochondrial inner membrane protein OXA1L OS=Homo sapiens OX=9606 GN=OXA1L PE=1 SV=3	43	14	14	9
Q96I24	Far upstream element-binding protein 3 OS=Homo sapiens OX=9606 GN=FUBP3 PE=1 SV=2	43	24	0	0
Q9BUJ2	Heterogeneous nuclear ribonucleoprotein U-like protein 1 OS=Homo sapiens OX=9606 GN=HNRNPUL1 PE=1 SV=2	42	20	4	4
Q8NBP0	Tetratricopeptide repeat protein 13 OS=Homo sapiens OX=9606 GN=TTC13 PE=2 SV=3	42	25	0	0
P30837	Aldehyde dehydrogenase X, mitochondrial OS=Homo sapiens OX=9606 GN=ALDH1B1 PE=1 SV=3	41	17	2	2
Q9NY65	Tubulin alpha-8 chain OS=Homo sapiens OX=9606 GN=TUBA8 PE=1 SV=1	41	1	0	0
O14980	Exportin-1 OS=Homo sapiens OX=9606 GN=XPO1 PE=1 SV=1	41	25	7	7
O95470	Sphingosine-1-phosphate lyase 1 OS=Homo sapiens OX=9606 GN=SGPL1 PE=1 SV=3	41	23	14	12
P51116	Fragile X mental retardation syndrome-related protein 2 OS=Homo sapiens OX=9606 GN=FXR2 PE=1 SV=2	40	13	0	0
P54136	Arginine--tRNA ligase, cytoplasmic OS=Homo sapiens OX=9606 GN=RARS PE=1 SV=2	40	25	8	8
Q96AE7	Tetratricopeptide repeat protein 17 OS=Homo sapiens OX=9606 GN=TTC17 PE=1 SV=1	40	25	2	2
P06493	Cyclin-dependent kinase 1 OS=Homo sapiens OX=9606 GN=CDK1 PE=1 SV=3	39	18	8	5
P42694	Probable helicase with zinc finger domain OS=Homo sapiens OX=9606 GN=HELZ PE=1 SV=2	39	26	0	0
O95299	NADH dehydrogenase [ubiquinone] 1 alpha subcomplex subunit 10, mitochondrial OS=Homo sapiens OX=9606 GN=NDUFA10 PE=1 SV=1	39	15	15	9
P48735	Isocitrate dehydrogenase [NADP], mitochondrial OS=Homo sapiens OX=9606 GN=IDH2 PE=1 SV=2	39	18	3	3
Q9NZI8	Insulin-like growth factor 2 mRNA-binding protein 1 OS=Homo sapiens OX=9606 GN=IGF2BP1 PE=1 SV=2	39	20	5	3
P57088	Transmembrane protein 33 OS=Homo sapiens OX=9606 GN=TMEM33 PE=1 SV=2	38	9	11	6
P52948	Nuclear pore complex protein Nup98-Nup96 OS=Homo sapiens OX=9606 GN=NUP98 PE=1 SV=4	37	22	1	1

P22695	Cytochrome b-c1 complex subunit 2, mitochondrial OS=Homo sapiens OX=9606 GN=UQCRC2 PE=1 SV=3	37	18	11	7
P47897	Glutamine--tRNA ligase OS=Homo sapiens OX=9606 GN=QARS PE=1 SV=1	36	25	3	3
O94874	E3 UFM1-protein ligase 1 OS=Homo sapiens OX=9606 GN=UFL1 PE=1 SV=2	34	26	7	7
Q14694	Ubiquitin carboxyl-terminal hydrolase 10 OS=Homo sapiens OX=9606 GN=USP10 PE=1 SV=2	34	21	1	1
Q14739	Lamin-B receptor OS=Homo sapiens OX=9606 GN=LBR PE=1 SV=2	34	12	2	2
Q08211	ATP-dependent RNA helicase A OS=Homo sapiens OX=9606 GN=DHX9 PE=1 SV=4	34	26	17	16
Q5JPH6	Probable glutamate--tRNA ligase, mitochondrial OS=Homo sapiens OX=9606 GN=EARS2 PE=1 SV=2	34	18	5	4
Q8N1F7	Nuclear pore complex protein Nup93 OS=Homo sapiens OX=9606 GN=NUP93 PE=1 SV=2	34	28	4	3
Q9BWF3	RNA-binding protein 4 OS=Homo sapiens OX=9606 GN=RBM4 PE=1 SV=1	33	4	4	4
P14868	Aspartate--tRNA ligase, cytoplasmic OS=Homo sapiens OX=9606 GN=DARS PE=1 SV=2	31	20	6	5
Q9BYJ9	YTH domain-containing family protein 1 OS=Homo sapiens OX=9606 GN=YTHDF1 PE=1 SV=1	31	11	0	0
P31943	Heterogeneous nuclear ribonucleoprotein H OS=Homo sapiens OX=9606 GN=HNRNPH1 PE=1 SV=4	31	6	7	3
Q9BUN8	Derlin-1 OS=Homo sapiens OX=9606 GN=DERL1 PE=1 SV=1	31	7	5	5
Q9Y285	Phenylalanine--tRNA ligase alpha subunit OS=Homo sapiens OX=9606 GN=FARSA PE=1 SV=3	31	15	12	10
P02533	Keratin, type I cytoskeletal 14 OS=Homo sapiens OX=9606 GN=KRT14 PE=1 SV=4	30	6	13	2
O00425	Insulin-like growth factor 2 mRNA-binding protein 3 OS=Homo sapiens OX=9606 GN=IGF2BP3 PE=1 SV=2	30	13	4	3
P41252	Isoleucine--tRNA ligase, cytoplasmic OS=Homo sapiens OX=9606 GN=IARS PE=1 SV=2	30	25	2	2
Q969V3	Nicalin OS=Homo sapiens OX=9606 GN=NCLN PE=1 SV=2	29	16	7	7
Q9BQ04	RNA-binding protein 4B OS=Homo sapiens OX=9606 GN=RBM4B PE=1 SV=1	29	2	0	0
Q6PKG0	La-related protein 1 OS=Homo sapiens OX=9606 GN=LARP1 PE=1 SV=2	29	23	4	4
P16615	Sarcoplasmic/endoplasmic reticulum calcium ATPase 2 OS=Homo sapiens OX=9606 GN=ATP2A2 PE=1 SV=1	28	17	11	8
Q92841	Probable ATP-dependent RNA helicase DDX17 OS=Homo sapiens OX=9606 GN=DDX17 PE=1 SV=2	28	17	14	6
P48634	Protein PRRC2A OS=Homo sapiens OX=9606 GN=PRRC2A PE=1 SV=3	28	22	5	5
P04181	Ornithine aminotransferase, mitochondrial OS=Homo sapiens OX=9606 GN=OAT PE=1 SV=1	28	17	1	1
Q15717	ELAV-like protein 1 OS=Homo sapiens OX=9606 GN=ELAVL1 PE=1 SV=2	28	13	5	5
P02786	Transferrin receptor protein 1 OS=Homo sapiens OX=9606 GN=TFRC PE=1 SV=2	28	19	8	8
Q9NXE4	Sphingomyelin phosphodiesterase 4 OS=Homo sapiens OX=9606 GN=SMPD4 PE=1 SV=3	27	19	3	3
Q99567	Nuclear pore complex protein Nup88 OS=Homo sapiens OX=9606 GN=NUP88 PE=1 SV=2	27	15	1	1
Q92667	A-kinase anchor protein 1, mitochondrial OS=Homo sapiens OX=9606 GN=AKAP1 PE=1 SV=1	27	14	0	0
P23258	Tubulin gamma-1 chain OS=Homo sapiens OX=9606 GN=TUBG1 PE=1 SV=2	27	12	3	3
P04259	Keratin, type II cytoskeletal 6B OS=Homo sapiens OX=9606 GN=KRT6B PE=1 SV=5	27	2	0	0
O60427	Fatty acid desaturase 1 OS=Homo sapiens OX=9606 GN=FADS1 PE=1 SV=3	26	15	5	5
P02768	Serum albumin OS=Homo sapiens OX=9606 GN=ALB PE=1 SV=2	26	7	8	5
P26599	Polypyrimidine tract-binding protein 1 OS=Homo sapiens OX=9606 GN=PTBP1 PE=1 SV=1	26	13	7	5
P43490	Nicotinamide phosphoribosyltransferase OS=Homo sapiens OX=9606 GN=NAMPT PE=1 SV=1	26	15	2	2
Q92545	Transmembrane protein 131 OS=Homo sapiens OX=9606 GN=TMEM131 PE=1 SV=3	26	20	0	0
P67809	Nuclease-sensitive element-binding protein 1 OS=Homo sapiens OX=9606 GN=YBX1 PE=1 SV=3	26	9	3	2
P55060	Exportin-2 OS=Homo sapiens OX=9606 GN=CSE1L PE=1 SV=3	25	19	8	8
P52597	Heterogeneous nuclear ribonucleoprotein F OS=Homo sapiens OX=9606 GN=HNRNPF PE=1 SV=3	25	10	7	4
Q14444	Caprin-1 OS=Homo sapiens OX=9606 GN=CAPRIN1 PE=1 SV=2	25	15	1	1
Q92947	Glutaryl-CoA dehydrogenase, mitochondrial OS=Homo sapiens OX=9606 GN=GCDH PE=1 SV=1	25	10	3	3
O95373	Importin-7 OS=Homo sapiens OX=9606 GN=IPO7 PE=1 SV=1	24	15	3	3
Q8WWM7	Ataxin-2-like protein OS=Homo sapiens OX=9606 GN=ATXN2L PE=1 SV=2	24	17	0	0
P11310	Medium-chain specific acyl-CoA dehydrogenase, mitochondrial OS=Homo sapiens OX=9606 GN=ACADM PE=1 SV=1	24	18	0	0
P56192	Methionine--tRNA ligase, cytoplasmic OS=Homo sapiens OX=9606 GN=MARS PE=1 SV=2	24	18	6	5
Q9H9B4	Sideroflexin-1 OS=Homo sapiens OX=9606 GN=SFXN1 PE=1 SV=4	23	14	7	6
Q03112	MDS1 and EVI1 complex locus protein OS=Homo sapiens OX=9606 GN=MECOM PE=1 SV=3	22	11	3	3
Q9H2U1	ATP-dependent RNA helicase DHX36 OS=Homo sapiens OX=9606 GN=DHX36 PE=1 SV=2	22	15	1	1
O43823	A-kinase anchor protein 8 OS=Homo sapiens OX=9606 GN=AKAP8 PE=1 SV=1	21	14	5	4
Q9HCE1	Putative helicase MOV-10 OS=Homo sapiens OX=9606 GN=MOV10 PE=1 SV=2	21	19	0	0
Q7Z417	Nuclear fragile X mental retardation-interacting protein 2 OS=Homo sapiens OX=9606 GN=NUFIP2 PE=1 SV=1	21	15	8	8
Q9NUL3	Double-stranded RNA-binding protein Staufen homolog 2 OS=Homo sapiens OX=9606 GN=STAU2 PE=1 SV=2	21	13	0	0
Q17RY0	Cytoplasmic polyadenylation element-binding protein 4 OS=Homo sapiens OX=9606 GN=CPEB4 PE=1 SV=1	21	16	1	1
O14949	Cytochrome b-c1 complex subunit 8 OS=Homo sapiens OX=9606 GN=UQCRQ PE=1 SV=4	21	10	7	3
P39748	Flap endonuclease 1 OS=Homo sapiens OX=9606 GN=FEN1 PE=1 SV=1	21	11	0	0
P78406	mRNA export factor OS=Homo sapiens OX=9606 GN=RAE1 PE=1 SV=1	21	11	3	3

O95793	Double-stranded RNA-binding protein Staufen homolog 1 OS=Homo sapiens OX=9606 GN=STAU1 PE=1 SV=2	20	13	5	5
Q9BYC5	Alpha-(1,6)-fucosyltransferase OS=Homo sapiens OX=9606 GN=FUT8 PE=1 SV=2	20	16	6	6
Q9Y5A9	YTH domain-containing family protein 2 OS=Homo sapiens OX=9606 GN=YTHDF2 PE=1 SV=2	20	7	0	0
Q53GQ0	Very-long-chain 3-oxoacyl-CoA reductase OS=Homo sapiens OX=9606 GN=HSD17B12 PE=1 SV=2	20	10	10	8
Q7Z739	YTH domain-containing family protein 3 OS=Homo sapiens OX=9606 GN=YTHDF3 PE=1 SV=1	20	6	0	0
O60573	Eukaryotic translation initiation factor 4E type 2 OS=Homo sapiens OX=9606 GN=EIF4E2 PE=1 SV=1	20	9	9	6
A1L0T0	Acetolactate synthase-like protein OS=Homo sapiens OX=9606 GN=ILVBL PE=1 SV=2	20	13	1	1
P03956	Interstitial collagenase OS=Homo sapiens OX=9606 GN=MMP1 PE=1 SV=3	20	17	8	7
Q9UN86	Ras GTPase-activating protein-binding protein 2 OS=Homo sapiens OX=9606 GN=G3BP2 PE=1 SV=2	20	11	6	4
Q9NX63	MICOS complex subunit MIC19 OS=Homo sapiens OX=9606 GN=CHCHD3 PE=1 SV=1	19	11	6	4
Q8NDV7	Trinucleotide repeat-containing gene 6A protein OS=Homo sapiens OX=9606 GN=TNRC6A PE=1 SV=2	19	13	1	1
O15063	Uncharacterized protein KIAA0355 OS=Homo sapiens OX=9606 GN=KIAA0355 PE=1 SV=2	19	12	0	0
Q3ZCQ8	Mitochondrial import inner membrane translocase subunit TIM50 OS=Homo sapiens OX=9606 GN=TIMM50 PE=1 SV=2	19	10	9	6
Q8TB72	Pumilio homolog 2 OS=Homo sapiens OX=9606 GN=PUM2 PE=1 SV=2	18	11	0	0
O43175	D-3-phosphoglycerate dehydrogenase OS=Homo sapiens OX=9606 GN=PHGDH PE=1 SV=4	18	10	3	3
Q63HK5	Teashirt homolog 3 OS=Homo sapiens OX=9606 GN=TSHZ3 PE=1 SV=2	18	14	2	2
P17812	CTP synthase 1 OS=Homo sapiens OX=9606 GN=CTPS1 PE=1 SV=2	18	13	3	3
P55795	Heterogeneous nuclear ribonucleoprotein H2 OS=Homo sapiens OX=9606 GN=HNRNPH2 PE=1 SV=1	18	3	0	0
Q71RC2	La-related protein 4 OS=Homo sapiens OX=9606 GN=LARP4 PE=1 SV=3	18	9	4	3
P48643	T-complex protein 1 subunit epsilon OS=Homo sapiens OX=9606 GN=CCT5 PE=1 SV=1	18	14	3	3
P33121	Long-chain-fatty-acid--CoA ligase 1 OS=Homo sapiens OX=9606 GN=ACSL1 PE=1 SV=1	17	12	4	4
Q13505	Metaxin-1 OS=Homo sapiens OX=9606 GN=MTX1 PE=1 SV=3	17	11	2	2
Q86UP2	Kinectin OS=Homo sapiens OX=9606 GN=KTN1 PE=1 SV=1	17	16	3	3
Q8IWZ3	Ankyrin repeat and KH domain-containing protein 1 OS=Homo sapiens OX=9606 GN=ANKHD1 PE=1 SV=1	17	10	0	0
Q9Y5V3	Melanoma-associated antigen D1 OS=Homo sapiens OX=9606 GN=MAGED1 PE=1 SV=3	17	15	0	0
P45880	Voltage-dependent anion-selective channel protein 2 OS=Homo sapiens OX=9606 GN=VDAC2 PE=1 SV=2	17	8	4	3
O00116	Alkyldihydroxyacetonephosphate synthase, peroxisomal OS=Homo sapiens OX=9606 GN=AGPS PE=1 SV=1	17	11	2	2
O14925	Mitochondrial import inner membrane translocase subunit Tim23 OS=Homo sapiens OX=9606 GN=TIMM23 PE=1 SV=1	17	8	0	0
O00767	Acyl-CoA desaturase OS=Homo sapiens OX=9606 GN=SCD PE=1 SV=2	17	6	1	1
O00165	HCLS1-associated protein X-1 OS=Homo sapiens OX=9606 GN=HAX1 PE=1 SV=2	16	11	7	5
P17987	T-complex protein 1 subunit alpha OS=Homo sapiens OX=9606 GN=TCP1 PE=1 SV=1	16	11	6	6
Q9UKV8	Protein argonaute-2 OS=Homo sapiens OX=9606 GN=AGO2 PE=1 SV=3	16	9	0	0
O00410	Importin-5 OS=Homo sapiens OX=9606 GN=IPO5 PE=1 SV=4	16	11	0	0
Q9Y520	Protein PRRC2C OS=Homo sapiens OX=9606 GN=PRRC2C PE=1 SV=4	16	12	5	4
O43251	RNA binding protein fox-1 homolog 2 OS=Homo sapiens OX=9606 GN=RBFOX2 PE=1 SV=3	15	9	3	2
O94808	Glutamine--fructose-6-phosphate aminotransferase [isomerizing] 2 OS=Homo sapiens OX=9606 GN=GFPT2 PE=1 SV=3	15	9	4	2
Q92499	ATP-dependent RNA helicase DDX1 OS=Homo sapiens OX=9606 GN=DDX1 PE=1 SV=2	15	10	2	2
Q96EY1	DnaJ homolog subfamily A member 3, mitochondrial OS=Homo sapiens OX=9606 GN=DNAJA3 PE=1 SV=2	15	8	7	5
Q5HYI7	Metaxin-3 OS=Homo sapiens OX=9606 GN=MTX3 PE=1 SV=2	15	12	0	0
P51153	Ras-related protein Rab-13 OS=Homo sapiens OX=9606 GN=RAB13 PE=1 SV=1	15	8	4	2
Q9UPQ9	Trinucleotide repeat-containing gene 6B protein OS=Homo sapiens OX=9606 GN=TNRC6B PE=1 SV=4	15	13	0	0
Q9BW92	Threonine--tRNA ligase, mitochondrial OS=Homo sapiens OX=9606 GN=TARS2 PE=1 SV=1	14	13	0	0
P61978	Heterogeneous nuclear ribonucleoprotein K OS=Homo sapiens OX=9606 GN=HNRNPK PE=1 SV=1	14	12	7	7
Q15046	Lysine--tRNA ligase OS=Homo sapiens OX=9606 GN=KARS PE=1 SV=3	14	12	3	3
Q8NE86	Calcium uniporter protein, mitochondrial OS=Homo sapiens OX=9606 GN=MCU PE=1 SV=1	14	9	2	1
Q29RF7	Sister chromatid cohesion protein PDS5 homolog A OS=Homo sapiens OX=9606 GN=PDS5A PE=1 SV=1	14	11	6	5
P61353	60S ribosomal protein L27 OS=Homo sapiens OX=9606 GN=RPL27 PE=1 SV=2	14	6	7	5
Q9H9G7	Protein argonaute-3 OS=Homo sapiens OX=9606 GN=AGO3 PE=1 SV=2	14	7	0	0
P16989	Y-box-binding protein 3 OS=Homo sapiens OX=9606 GN=YBX3 PE=1 SV=4	14	3	2	1
O75947	ATP synthase subunit d, mitochondrial OS=Homo sapiens OX=9606 GN=ATP5H PE=1 SV=3	14	9	5	5
Q12904	Aminoacyl tRNA synthase complex-interacting multifunctional protein 1 OS=Homo sapiens OX=9606 GN=AIMP1 PE=1 SV=2	14	8	0	0
Q9UG63	ATP-binding cassette sub-family F member 2 OS=Homo sapiens OX=9606 GN=ABCF2 PE=1 SV=2	14	11	4	3
Q9NZB2	Constitutive coactivator of PPAR-gamma-like protein 1 OS=Homo sapiens OX=9606 GN=FAM120A PE=1 SV=2	14	12	0	0
Q6P444	Mitochondrial fission regulator 2 OS=Homo sapiens OX=9606 GN=MTFR2 PE=1 SV=2	13	12	4	4
P63010	AP-2 complex subunit beta OS=Homo sapiens OX=9606 GN=AP2B1 PE=1 SV=1	13	12	5	5
Q8NI60	Atypical kinase COQ8A, mitochondrial OS=Homo sapiens OX=9606 GN=COQ8A PE=1 SV=1	13	7	4	4

P28288	ATP-binding cassette sub-family D member 3 OS=Homo sapiens OX=9606 GN=ABCD3 PE=1 SV=1	13	10	4	4
P34931	Heat shock 70 kDa protein 1-like OS=Homo sapiens OX=9606 GN=HSPA1L PE=1 SV=2	13	2	0	0
P38606	V-type proton ATPase catalytic subunit A OS=Homo sapiens OX=9606 GN=ATP6V1A PE=1 SV=2	13	10	0	0
O75323	Protein NipSnap homolog 2 OS=Homo sapiens OX=9606 GN=NIPSNAP2 PE=1 SV=1	13	9	4	2
Q8NBM4	Ubiquitin-associated domain-containing protein 2 OS=Homo sapiens OX=9606 GN=UBAC2 PE=1 SV=1	13	10	6	5
Q7Z2W4	Zinc finger CCCH-type antiviral protein 1 OS=Homo sapiens OX=9606 GN=ZC3HAV1 PE=1 SV=3	13	13	1	1
Q96AE4	Far upstream element-binding protein 1 OS=Homo sapiens OX=9606 GN=FUBP1 PE=1 SV=3	13	3	0	0
Q96PV6	Leukocyte receptor cluster member 8 OS=Homo sapiens OX=9606 GN=LENG8 PE=1 SV=3	13	10	0	0
Q8TEQ6	Gem-associated protein 5 OS=Homo sapiens OX=9606 GN=GEMIN5 PE=1 SV=3	12	10	0	0
Q96CW1	AP-2 complex subunit mu OS=Homo sapiens OX=9606 GN=AP2M1 PE=1 SV=2	12	9	2	2
O95782	AP-2 complex subunit alpha-1 OS=Homo sapiens OX=9606 GN=AP2A1 PE=1 SV=3	12	8	2	2
Q5JTZ9	Alanine--tRNA ligase, mitochondrial OS=Homo sapiens OX=9606 GN=AARS2 PE=1 SV=1	12	10	1	1
O75911	Short-chain dehydrogenase/reductase 3 OS=Homo sapiens OX=9606 GN=DHRS3 PE=1 SV=2	12	8	3	3
P24941	Cyclin-dependent kinase 2 OS=Homo sapiens OX=9606 GN=CDK2 PE=1 SV=2	12	6	0	0
O95628	CCR4-NOT transcription complex subunit 4 OS=Homo sapiens OX=9606 GN=CNOT4 PE=1 SV=3	12	12	0	0
Q9Y6M1	Insulin-like growth factor 2 mRNA-binding protein 2 OS=Homo sapiens OX=9606 GN=IGF2BP2 PE=1 SV=2	12	7	0	0
Q96AQ6	Pre-B-cell leukemia transcription factor-interacting protein 1 OS=Homo sapiens OX=9606 GN=PBXIP1 PE=1 SV=1	12	10	4	3
Q9UBM7	7-dehydrocholesterol reductase OS=Homo sapiens OX=9606 GN=DHCR7 PE=1 SV=1	12	7	3	3
Q04695	Keratin, type I cytoskeletal 17 OS=Homo sapiens OX=9606 GN=KRT17 PE=1 SV=2	12	1	0	0
P62979	Ubiquitin-40S ribosomal protein S27a OS=Homo sapiens OX=9606 GN=RPS27A PE=1 SV=2	12	8	6	1
Q9Y3I0	tRNA-splicing ligase RtcB homolog OS=Homo sapiens OX=9606 GN=RTCB PE=1 SV=1	12	10	0	0
Q16822	Phosphoenolpyruvate carboxykinase [GTP], mitochondrial OS=Homo sapiens OX=9606 GN=PCK2 PE=1 SV=4	12	10	0	0
Q5SRE5	Nucleoporin NUP188 homolog OS=Homo sapiens OX=9606 GN=NUP188 PE=1 SV=1	12	12	0	0
P39656	Dolichyl-diphosphooligosaccharide--protein glycosyltransferase 48 kDa subunit OS=Homo sapiens OX=9606 GN=DDOST PE=1 SV=4	12	8	5	5
P06733	Alpha-enolase OS=Homo sapiens OX=9606 GN=ENO1 PE=1 SV=2	11	9	3	2
O75420	GRB10-interacting GYF protein 1 OS=Homo sapiens OX=9606 GN=GIGYF1 PE=1 SV=2	11	10	0	0
Q14697	Neutral alpha-glucosidase AB OS=Homo sapiens OX=9606 GN=GANAB PE=1 SV=3	11	10	0	0
P00367	Glutamate dehydrogenase 1, mitochondrial OS=Homo sapiens OX=9606 GN=GLUD1 PE=1 SV=2	11	8	4	4
Q96K37	Solute carrier family 35 member E1 OS=Homo sapiens OX=9606 GN=SLC35E1 PE=1 SV=2	11	8	2	2
Q12906	Interleukin enhancer-binding factor 3 OS=Homo sapiens OX=9606 GN=ILF3 PE=1 SV=3	11	7	4	4
Q96G23	Ceramide synthase 2 OS=Homo sapiens OX=9606 GN=CERS2 PE=1 SV=1	11	6	2	2
Q06210	Glutamine--fructose-6-phosphate aminotransferase [isomerizing] 1 OS=Homo sapiens OX=9606 GN=GFPT1 PE=1 SV=3	11	6	5	3
Q86VP6	Cullin-associated NEDD8-dissociated protein 1 OS=Homo sapiens OX=9606 GN=CAND1 PE=1 SV=2	11	11	0	0
Q9NPL8	Complex I assembly factor TIMMDC1, mitochondrial OS=Homo sapiens OX=9606 GN=TIMMDC1 PE=1 SV=2	11	10	5	5
O15258	Protein RER1 OS=Homo sapiens OX=9606 GN=RER1 PE=1 SV=1	11	5	3	1
Q9H0A0	RNA cytidine acetyltransferase OS=Homo sapiens OX=9606 GN=NAT10 PE=1 SV=2	10	8	5	5
P04792	Heat shock protein beta-1 OS=Homo sapiens OX=9606 GN=HSPB1 PE=1 SV=2	10	7	2	2
Q6ZRY4	RNA-binding protein with multiple splicing 2 OS=Homo sapiens OX=9606 GN=RBPMS2 PE=1 SV=1	10	4	0	0
Q8IWZ8	SURP and G-patch domain-containing protein 1 OS=Homo sapiens OX=9606 GN=SUGP1 PE=1 SV=2	10	9	4	4
P84095	Rho-related GTP-binding protein RhoG OS=Homo sapiens OX=9606 GN=RHOG PE=1 SV=1	10	7	0	0
Q5HYK3	2-methoxy-6-polyprenyl-1,4-benzoquinol methylase, mitochondrial OS=Homo sapiens OX=9606 GN=COQ5 PE=1 SV=2	10	9	1	1
P12268	Inosine-5'-monophosphate dehydrogenase 2 OS=Homo sapiens OX=9606 GN=IMPDH2 PE=1 SV=2	10	9	1	1
O96011	Peroxisomal membrane protein 11B OS=Homo sapiens OX=9606 GN=PEX11B PE=1 SV=1	10	8	3	3
Q9Y3T9	Nucleolar complex protein 2 homolog OS=Homo sapiens OX=9606 GN=NOC2L PE=1 SV=4	9	9	4	4
O43913	Origin recognition complex subunit 5 OS=Homo sapiens OX=9606 GN=ORC5 PE=1 SV=1	9	8	1	1
Q16635	Tafazzin OS=Homo sapiens OX=9606 GN=TAZ PE=1 SV=1	9	5	0	0
Q9Y2G8	DnaJ homolog subfamily C member 16 OS=Homo sapiens OX=9606 GN=DNAJC16 PE=2 SV=3	9	8	2	2
P21796	Voltage-dependent anion-selective channel protein 1 OS=Homo sapiens OX=9606 GN=VDAC1 PE=1 SV=2	9	5	3	3
Q5C9Z4	Nucleolar MIF4G domain-containing protein 1 OS=Homo sapiens OX=9606 GN=NOM1 PE=1 SV=1	9	8	4	3
Q96KA5	Cleft lip and palate transmembrane protein 1-like protein OS=Homo sapiens OX=9606 GN=CLPTM1L PE=1 SV=1	9	6	0	0
Q68CQ7	Glycosyltransferase 8 domain-containing protein 1 OS=Homo sapiens OX=9606 GN=GLT8D1 PE=1 SV=2	9	9	1	1
P0C0S8	Histone H2A type 1 OS=Homo sapiens OX=9606 GN=HIST1H2AG PE=1 SV=2	9	2	0	0
P18085	ADP-ribosylation factor 4 OS=Homo sapiens OX=9606 GN=ARF4 PE=1 SV=3	9	2	3	2
P21127	Cyclin-dependent kinase 11B OS=Homo sapiens OX=9606 GN=CDK11B PE=1 SV=4	9	7	4	4
P62826	GTP-binding nuclear protein Ran OS=Homo sapiens OX=9606 GN=RAN PE=1 SV=3	9	5	4	4
Q96DH6	RNA-binding protein Musashi homolog 2 OS=Homo sapiens OX=9606 GN=MSI2 PE=1 SV=1	9	3	0	0

Q99570	Phosphoinositide 3-kinase regulatory subunit 4 OS=Homo sapiens OX=9606 GN=PIK3R4 PE=1 SV=3	9	9	3	2
Q9BWS9	Chitinase domain-containing protein 1 OS=Homo sapiens OX=9606 GN=CHID1 PE=1 SV=1	9	7	2	2
Q9ULX6	A-kinase anchor protein 8-like OS=Homo sapiens OX=9606 GN=AKAP8L PE=1 SV=3	9	7	3	3
Q6JQN1	Acyl-CoA dehydrogenase family member 10 OS=Homo sapiens OX=9606 GN=ACAD10 PE=1 SV=1	9	8	0	0
P29558	RNA-binding motif, single-stranded-interacting protein 1 OS=Homo sapiens OX=9606 GN=RBMS1 PE=1 SV=3	9	3	0	0
Q9BPW8	Protein NipSnap homolog 1 OS=Homo sapiens OX=9606 GN=NIPSNAP1 PE=1 SV=1	9	4	3	2
Q8TB61	Adenosine 3'-phospho 5'-phosphosulfate transporter 1 OS=Homo sapiens OX=9606 GN=SLC35B2 PE=1 SV=1	9	6	3	2
Q15434	RNA-binding motif, single-stranded-interacting protein 2 OS=Homo sapiens OX=9606 GN=RBMS2 PE=1 SV=1	9	3	0	0
Q9Y6E2	Basic leucine zipper and W2 domain-containing protein 2 OS=Homo sapiens OX=9606 GN=BZW2 PE=1 SV=1	9	6	3	2
Q02338	D-beta-hydroxybutyrate dehydrogenase, mitochondrial OS=Homo sapiens OX=9606 GN=BDH1 PE=1 SV=3	9	7	3	2
Q53EU6	Glycerol-3-phosphate acyltransferase 3 OS=Homo sapiens OX=9606 GN=GPAT3 PE=1 SV=2	9	8	1	1
P35658	Nuclear pore complex protein Nup214 OS=Homo sapiens OX=9606 GN=NUP214 PE=1 SV=2	9	8	0	0
Q96I25	Splicing factor 45 OS=Homo sapiens OX=9606 GN=RBM17 PE=1 SV=1	8	7	2	2
Q92621	Nuclear pore complex protein Nup205 OS=Homo sapiens OX=9606 GN=NUP205 PE=1 SV=3	8	8	2	2
Q96A46	Mitoferrin-2 OS=Homo sapiens OX=9606 GN=SLC25A28 PE=2 SV=1	8	6	0	0
O95870	Protein ABHD16A OS=Homo sapiens OX=9606 GN=ABHD16A PE=1 SV=3	8	6	1	1
Q09028	Histone-binding protein RBBP4 OS=Homo sapiens OX=9606 GN=RBBP4 PE=1 SV=3	8	5	4	3
Q9P0J1	[Pyruvate dehydrogenase [acetyl-transferring]]-phosphatase 1, mitochondrial OS=Homo sapiens OX=9606 GN=PDP1 PE=1 SV=3	8	8	0	0
Q9HAZ2	PR domain zinc finger protein 16 OS=Homo sapiens OX=9606 GN=PRDM16 PE=1 SV=3	8	3	0	0
Q9BT22	Chitobiosyldiphosphodolichol beta-mannosyltransferase OS=Homo sapiens OX=9606 GN=ALG1 PE=1 SV=2	8	6	4	3
Q5J8M3	ER membrane protein complex subunit 4 OS=Homo sapiens OX=9606 GN=EMC4 PE=1 SV=2	8	4	3	1
Q13501	Sequestosome-1 OS=Homo sapiens OX=9606 GN=SQSTM1 PE=1 SV=1	8	5	4	1
Q9H845	Acyl-CoA dehydrogenase family member 9, mitochondrial OS=Homo sapiens OX=9606 GN=ACAD9 PE=1 SV=1	8	7	0	0
Q5T8D3	Acyl-CoA-binding domain-containing protein 5 OS=Homo sapiens OX=9606 GN=ACBD5 PE=1 SV=1	8	6	0	0
Q5BJH7	Protein YIF1B OS=Homo sapiens OX=9606 GN=YIF1B PE=1 SV=1	8	5	2	2
Q9Y2Q3	Glutathione S-transferase kappa 1 OS=Homo sapiens OX=9606 GN=GSTK1 PE=1 SV=3	8	5	0	0
Q9Y512	Sorting and assembly machinery component 50 homolog OS=Homo sapiens OX=9606 GN=SAMM50 PE=1 SV=3	8	7	4	4
O43264	Centromere/kinetochore protein zw10 homolog OS=Homo sapiens OX=9606 GN=ZW10 PE=1 SV=3	8	7	0	0
Q6DD88	Atlastin-3 OS=Homo sapiens OX=9606 GN=ATL3 PE=1 SV=1	8	7	0	0
Q9P0J0	NADH dehydrogenase [ubiquinone] 1 alpha subcomplex subunit 13 OS=Homo sapiens OX=9606 GN=NDUFA13 PE=1 SV=3	8	5	4	4
Q6P9B9	Integrator complex subunit 5 OS=Homo sapiens OX=9606 GN=INTS5 PE=1 SV=1	8	6	3	2
O95985	DNA topoisomerase 3-beta-1 OS=Homo sapiens OX=9606 GN=TOP3B PE=1 SV=1	8	7	0	0
Q9H2D1	Mitochondrial folate transporter/carrier OS=Homo sapiens OX=9606 GN=SLC25A32 PE=1 SV=2	8	6	1	1
O60264	SWI/SNF-related matrix-associated actin-dependent regulator of chromatin subfamily A member 5 OS=Homo sapiens OX=9606 GN=SMARCA5 PE=1 SV=1	8	8	4	4
Q9NVM9	Integrator complex subunit 13 OS=Homo sapiens OX=9606 GN=INTS13 PE=1 SV=2	8	6	0	0
O75179	Ankyrin repeat domain-containing protein 17 OS=Homo sapiens OX=9606 GN=ANKRD17 PE=1 SV=3	8	3	0	0
Q7LGA3	Heparan sulfate 2-O-sulfotransferase 1 OS=Homo sapiens OX=9606 GN=HS2ST1 PE=1 SV=1	7	6	3	3
Q6KCM7	Calcium-binding mitochondrial carrier protein SCaMC-2 OS=Homo sapiens OX=9606 GN=SLC25A25 PE=1 SV=1	7	3	0	0
O43324	Eukaryotic translation elongation factor 1 epsilon-1 OS=Homo sapiens OX=9606 GN=EEF1E1 PE=1 SV=1	7	4	0	0
Q9UJZ1	Stomatin-like protein 2, mitochondrial OS=Homo sapiens OX=9606 GN=STOML2 PE=1 SV=1	7	6	2	2
Q5ST30	Valine--tRNA ligase, mitochondrial OS=Homo sapiens OX=9606 GN=VARS2 PE=1 SV=2	7	5	0	0
O95070	Protein YIF1A OS=Homo sapiens OX=9606 GN=YIF1A PE=1 SV=2	7	4	0	0
Q9NVH1	DnaJ homolog subfamily C member 11 OS=Homo sapiens OX=9606 GN=DNAJC11 PE=1 SV=2	7	5	3	3
Q9BRK5	45 kDa calcium-binding protein OS=Homo sapiens OX=9606 GN=SDF4 PE=1 SV=1	7	4	2	2
O00411	DNA-directed RNA polymerase, mitochondrial OS=Homo sapiens OX=9606 GN=POLRMT PE=1 SV=2	7	6	0	0
P37268	Squalene synthase OS=Homo sapiens OX=9606 GN=FDFT1 PE=1 SV=1	7	5	0	0
O15014	Zinc finger protein 609 OS=Homo sapiens OX=9606 GN=ZNF609 PE=1 SV=2	7	5	0	0
Q92604	Acyl-CoA:lysophosphatidylglycerol acyltransferase 1 OS=Homo sapiens OX=9606 GN=LPGAT1 PE=1 SV=1	7	7	3	3
Q9P2J5	Leucine--tRNA ligase, cytoplasmic OS=Homo sapiens OX=9606 GN=LARS PE=1 SV=2	7	7	0	0
Q53EP0	Fibronectin type III domain-containing protein 3B OS=Homo sapiens OX=9606 GN=FNDC3B PE=1 SV=2	7	5	0	0
Q9H857	5'-nucleotidase domain-containing protein 2 OS=Homo sapiens OX=9606 GN=NT5DC2 PE=1 SV=1	7	6	1	1
Q9UJX3	Anaphase-promoting complex subunit 7 OS=Homo sapiens OX=9606 GN=ANAPC7 PE=1 SV=4	7	7	0	0
P01116	GTPase KRas OS=Homo sapiens OX=9606 GN=KRAS PE=1 SV=1	7	3	1	1
Q13636	Ras-related protein Rab-31 OS=Homo sapiens OX=9606 GN=RAB31 PE=1 SV=1	7	6	0	0
Q86Y07	Serine/threonine-protein kinase VRK2 OS=Homo sapiens OX=9606 GN=VRK2 PE=1 SV=3	7	5	3	3
Q9H078	Caseinolytic peptidase B protein homolog OS=Homo sapiens OX=9606 GN=CLPB PE=1 SV=1	7	7	0	0

P61204	ADP-ribosylation factor 3 OS=Homo sapiens OX=9606 GN=ARF3 PE=1 SV=2	7	1	3	2
O75477	Erlin-1 OS=Homo sapiens OX=9606 GN=ERLIN1 PE=1 SV=1	7	3	1	1
Q00577	Transcriptional activator protein Pur-alpha OS=Homo sapiens OX=9606 GN=PURA PE=1 SV=2	7	5	0	0
Q92540	Protein SMG7 OS=Homo sapiens OX=9606 GN=SMG7 PE=1 SV=2	7	7	0	0
Q99653	Calcineurin B homologous protein 1 OS=Homo sapiens OX=9606 GN=CHP1 PE=1 SV=3	6	6	3	3
P57740	Nuclear pore complex protein Nup107 OS=Homo sapiens OX=9606 GN=NUP107 PE=1 SV=1	6	6	0	0
Q9C037	E3 ubiquitin-protein ligase TRIM4 OS=Homo sapiens OX=9606 GN=TRIM4 PE=1 SV=2	6	5	0	0
P27144	Adenylate kinase 4, mitochondrial OS=Homo sapiens OX=9606 GN=AK4 PE=1 SV=1	6	4	0	0
Q9H4M9	EH domain-containing protein 1 OS=Homo sapiens OX=9606 GN=EHD1 PE=1 SV=2	6	1	0	0
P18850	Cyclic AMP-dependent transcription factor ATF-6 alpha OS=Homo sapiens OX=9606 GN=ATF6 PE=1 SV=3	6	5	1	1
O75431	Metaxin-2 OS=Homo sapiens OX=9606 GN=MTX2 PE=1 SV=1	6	3	0	0
Q5VV42	Threonylcarbamoyladenosine tRNA methylthiotransferase OS=Homo sapiens OX=9606 GN=CDKAL1 PE=1 SV=1	6	6	1	1
Q9H9P8	L-2-hydroxyglutarate dehydrogenase, mitochondrial OS=Homo sapiens OX=9606 GN=L2HGDH PE=1 SV=3	6	5	0	0
P11586	C-1-tetrahydrofolate synthase, cytoplasmic OS=Homo sapiens OX=9606 GN=MTHFD1 PE=1 SV=3	6	6	0	0
O15119	T-box transcription factor TBX3 OS=Homo sapiens OX=9606 GN=TBX3 PE=1 SV=4	6	3	0	0
Q96T76	MMS19 nucleotide excision repair protein homolog OS=Homo sapiens OX=9606 GN=MMS19 PE=1 SV=2	6	6	0	0
Q9NP72	Ras-related protein Rab-18 OS=Homo sapiens OX=9606 GN=RAB18 PE=1 SV=1	6	5	3	3
Q9NZJ7	Mitochondrial carrier homolog 1 OS=Homo sapiens OX=9606 GN=MTCH1 PE=1 SV=1	6	4	0	0
Q8IXI1	Mitochondrial Rho GTPase 2 OS=Homo sapiens OX=9606 GN=RHOT2 PE=1 SV=2	6	4	2	2
Q9UL18	Protein argonaute-1 OS=Homo sapiens OX=9606 GN=AGO1 PE=1 SV=3	6	2	0	0
O43390	Heterogeneous nuclear ribonucleoprotein R OS=Homo sapiens OX=9606 GN=HNRNPR PE=1 SV=1	6	4	1	1
P55786	Puromycin-sensitive aminopeptidase OS=Homo sapiens OX=9606 GN=NPEPPS PE=1 SV=2	6	6	0	0
Q01085	Nucleolysin TIAR OS=Homo sapiens OX=9606 GN=TIAL1 PE=1 SV=1	6	3	0	0
P11177	Pyruvate dehydrogenase E1 component subunit beta, mitochondrial OS=Homo sapiens OX=9606 GN=PDHB PE=1 SV=3	6	5	0	0
Q9UBI6	Guanine nucleotide-binding protein G(I)/G(S)/G(O) subunit gamma-12 OS=Homo sapiens OX=9606 GN=GNG12 PE=1 SV=3	6	4	3	2
P00403	Cytochrome c oxidase subunit 2 OS=Homo sapiens OX=9606 GN=MT-CO2 PE=1 SV=1	6	3	1	1
Q9BW27	Nuclear pore complex protein Nup85 OS=Homo sapiens OX=9606 GN=NUP85 PE=1 SV=1	6	5	0	0
Q92928	Putative Ras-related protein Rab-1C OS=Homo sapiens OX=9606 GN=RAB1C PE=5 SV=2	6	4	0	0
Q9Y261	Hepatocyte nuclear factor 3-beta OS=Homo sapiens OX=9606 GN=FOXA2 PE=1 SV=1	6	3	0	0
Q9NZN3	EH domain-containing protein 3 OS=Homo sapiens OX=9606 GN=EHD3 PE=1 SV=2	6	1	0	0
Q53S58	Transmembrane protein 177 OS=Homo sapiens OX=9606 GN=TMEM177 PE=1 SV=1	6	4	2	2
Q9BQ39	ATP-dependent RNA helicase DDX50 OS=Homo sapiens OX=9606 GN=DDX50 PE=1 SV=1	6	5	3	2
Q9NUD5	Zinc finger CCHC domain-containing protein 3 OS=Homo sapiens OX=9606 GN=ZCCHC3 PE=1 SV=1	6	5	0	0
Q9NTX5	Ethylmalonyl-CoA decarboxylase OS=Homo sapiens OX=9606 GN=ECHDC1 PE=1 SV=2	6	5	1	1
Q9NTI5	Sister chromatid cohesion protein PDS5 homolog B OS=Homo sapiens OX=9606 GN=PDS5B PE=1 SV=1	6	5	1	1
O00506	Serine/threonine-protein kinase 25 OS=Homo sapiens OX=9606 GN=STK25 PE=1 SV=1	6	3	3	3
Q92611	ER degradation-enhancing alpha-mannosidase-like protein 1 OS=Homo sapiens OX=9606 GN=EDEM1 PE=1 SV=1	6	4	1	1
Q9NVR2	Integrator complex subunit 10 OS=Homo sapiens OX=9606 GN=INTS10 PE=1 SV=2	6	6	0	0
O00160	Unconventional myosin-If OS=Homo sapiens OX=9606 GN=MYO1F PE=1 SV=3	6	1	0	0
P05121	Plasminogen activator inhibitor 1 OS=Homo sapiens OX=9606 GN=SERPINE1 PE=1 SV=1	6	6	2	2
Q9BV35	Calcium-binding mitochondrial carrier protein SCaMC-3 OS=Homo sapiens OX=9606 GN=SLC25A23 PE=1 SV=2	6	3	0	0
Q8WUM0	Nuclear pore complex protein Nup133 OS=Homo sapiens OX=9606 GN=NUP133 PE=1 SV=2	6	6	1	1
Q13148	TAR DNA-binding protein 43 OS=Homo sapiens OX=9606 GN=TARDBP PE=1 SV=1	6	5	1	1
P48651	Phosphatidylserine synthase 1 OS=Homo sapiens OX=9606 GN=PTDSS1 PE=1 SV=1	6	5	3	3
Q96HY6	DDRGK domain-containing protein 1 OS=Homo sapiens OX=9606 GN=DDRGK1 PE=1 SV=2	6	5	2	2
P49207	60S ribosomal protein L34 OS=Homo sapiens OX=9606 GN=RPL34 PE=1 SV=3	6	6	3	3
Q93063	Exostosin-2 OS=Homo sapiens OX=9606 GN=EXT2 PE=1 SV=1	6	5	1	1
O95347	Structural maintenance of chromosomes protein 2 OS=Homo sapiens OX=9606 GN=SMC2 PE=1 SV=2	6	6	3	3
Q9BSJ2	Gamma-tubulin complex component 2 OS=Homo sapiens OX=9606 GN=TUBGCP2 PE=1 SV=2	6	6	1	1
Q9UGJ1	Gamma-tubulin complex component 4 OS=Homo sapiens OX=9606 GN=TUBGCP4 PE=1 SV=1	5	5	0	0
Q8N138	ORM1-like protein 3 OS=Homo sapiens OX=9606 GN=ORMDL3 PE=1 SV=1	5	1	0	0
Q9P0S3	ORM1-like protein 1 OS=Homo sapiens OX=9606 GN=ORMDL1 PE=1 SV=1	5	1	0	0
Q92609	TBC1 domain family member 5 OS=Homo sapiens OX=9606 GN=TBC1D5 PE=1 SV=1	5	5	0	0
Q96D53	Atypical kinase COQ8B, mitochondrial OS=Homo sapiens OX=9606 GN=COQ8B PE=1 SV=2	5	4	1	1
Q15007	Pre-mRNA-splicing regulator WTAP OS=Homo sapiens OX=9606 GN=WTAP PE=1 SV=2	5	4	2	2
Q9C0D9	Ethanolaminephosphotransferase 1 OS=Homo sapiens OX=9606 GN=SELENOI PE=1 SV=3	5	3	0	0

P46977	Dolichyl-diphosphooligosaccharide--protein glycosyltransferase subunit STT3A OS=Homo sapiens OX=9606 GN=STT3A PE=1 SV=2	5	5	2	2
Q9Y6M9	NADH dehydrogenase [ubiquinone] 1 beta subcomplex subunit 9 OS=Homo sapiens OX=9606 GN=NDUFB9 PE=1 SV=3	5	4	2	2
O75534	Cold shock domain-containing protein E1 OS=Homo sapiens OX=9606 GN=CSDE1 PE=1 SV=2	5	5	0	0
Q9UNL2	Translocon-associated protein subunit gamma OS=Homo sapiens OX=9606 GN=SSR3 PE=1 SV=1	5	2	1	1
Q02413	Desmoglein-1 OS=Homo sapiens OX=9606 GN=DSG1 PE=1 SV=2	5	5	1	1
O15121	Sphingolipid delta(4)-desaturase DES1 OS=Homo sapiens OX=9606 GN=DEGS1 PE=1 SV=1	5	3	0	0
Q7L8L6	FAST kinase domain-containing protein 5, mitochondrial OS=Homo sapiens OX=9606 GN=FASTKD5 PE=1 SV=1	5	5	2	2
B5ME19	Eukaryotic translation initiation factor 3 subunit C-like protein OS=Homo sapiens OX=9606 GN=EIF3CL PE=3 SV=1	5	5	2	2
Q9NPA8	Transcription and mRNA export factor ENY2 OS=Homo sapiens OX=9606 GN=ENY2 PE=1 SV=1	5	1	0	0
O14773	Tripeptidyl-peptidase 1 OS=Homo sapiens OX=9606 GN=TPP1 PE=1 SV=2	5	4	0	0
P28331	NADH-ubiquinone oxidoreductase 75 kDa subunit, mitochondrial OS=Homo sapiens OX=9606 GN=NDUFS1 PE=1 SV=3	5	5	1	1
Q96HV5	Transmembrane protein 41A OS=Homo sapiens OX=9606 GN=TMEM41A PE=1 SV=1	5	5	0	0
Q9BQD7	Protein N-lysine methyltransferase FAM173A OS=Homo sapiens OX=9606 GN=FAM173A PE=2 SV=1	5	4	2	2
P11498	Pyruvate carboxylase, mitochondrial OS=Homo sapiens OX=9606 GN=PC PE=1 SV=2	5	4	0	0
Q9BXW7	Haloacid dehalogenase-like hydrolase domain-containing 5 OS=Homo sapiens OX=9606 GN=HDHD5 PE=1 SV=1	5	4	0	0
Q8NHH9	Atlastin-2 OS=Homo sapiens OX=9606 GN=ATL2 PE=1 SV=2	5	4	0	0
Q8IZ52	Chondroitin sulfate synthase 2 OS=Homo sapiens OX=9606 GN=CHPF PE=1 SV=2	5	5	0	0
O15260	Surfeit locus protein 4 OS=Homo sapiens OX=9606 GN=SURF4 PE=1 SV=3	5	2	0	0
P00533	Epidermal growth factor receptor OS=Homo sapiens OX=9606 GN=EGFR PE=1 SV=2	5	4	2	2
Q86UE4	Protein LYRIC OS=Homo sapiens OX=9606 GN=MTDH PE=1 SV=2	5	5	0	0
Q13207	T-box transcription factor TBX2 OS=Homo sapiens OX=9606 GN=TBX2 PE=1 SV=3	5	2	0	0
Q8IWB1	Inositol 1,4,5-trisphosphate receptor-interacting protein OS=Homo sapiens OX=9606 GN=ITPRIP PE=1 SV=1	5	5	0	0
O94973	AP-2 complex subunit alpha-2 OS=Homo sapiens OX=9606 GN=AP2A2 PE=1 SV=2	5	1	0	0
Q14103	Heterogeneous nuclear ribonucleoprotein D0 OS=Homo sapiens OX=9606 GN=HNRNPD PE=1 SV=1	5	3	2	1
Q8IUH3	RNA-binding protein 45 OS=Homo sapiens OX=9606 GN=RBM45 PE=1 SV=1	5	5	0	0
P06702	Protein S100-A9 OS=Homo sapiens OX=9606 GN=S100A9 PE=1 SV=1	5	3	0	0
Q9H3U1	Protein unc-45 homolog A OS=Homo sapiens OX=9606 GN=UNC45A PE=1 SV=1	5	5	2	2
P03928	ATP synthase protein 8 OS=Homo sapiens OX=9606 GN=MT-ATP8 PE=1 SV=1	5	2	1	1
O94905	Erlin-2 OS=Homo sapiens OX=9606 GN=ERLIN2 PE=1 SV=1	5	1	0	0
Q9Y224	RNA transcription, translation and transport factor protein OS=Homo sapiens OX=9606 GN=RTRAF PE=1 SV=1	5	4	2	2
Q96GQ5	RUS1 family protein C16orf58 OS=Homo sapiens OX=9606 GN=C16orf58 PE=1 SV=2	5	5	0	0
Q07021	Complement component 1 Q subcomponent-binding protein, mitochondrial OS=Homo sapiens OX=9606 GN=C1QBP PE=1 SV=1	5	4	1	1
P15924	Desmoplakin OS=Homo sapiens OX=9606 GN=DSP PE=1 SV=3	5	5	0	0
O75694	Nuclear pore complex protein Nup155 OS=Homo sapiens OX=9606 GN=NUP155 PE=1 SV=1	5	4	1	1
O75340	Programmed cell death protein 6 OS=Homo sapiens OX=9606 GN=PDCD6 PE=1 SV=1	5	5	0	0
Q92503	SEC14-like protein 1 OS=Homo sapiens OX=9606 GN=SEC14L1 PE=1 SV=2	5	5	0	0
Q9Y4P3	Transducin beta-like protein 2 OS=Homo sapiens OX=9606 GN=TBL2 PE=1 SV=1	5	5	2	2
Q6PJG6	BRCA1-associated ATM activator 1 OS=Homo sapiens OX=9606 GN=BRAT1 PE=1 SV=2	5	5	0	0
P31151	Protein S100-A7 OS=Homo sapiens OX=9606 GN=S100A7 PE=1 SV=4	4	1	0	0
O60725	Protein-S-isoprenylcysteine O-methyltransferase OS=Homo sapiens OX=9606 GN=ICMT PE=1 SV=1	4	2	1	1
O95219	Sorting nexin-4 OS=Homo sapiens OX=9606 GN=SNX4 PE=1 SV=1	4	3	0	0
P63244	Receptor of activated protein C kinase 1 OS=Homo sapiens OX=9606 GN=RACK1 PE=1 SV=3	4	4	1	1
Q5JPE7	Nodal modulator 2 OS=Homo sapiens OX=9606 GN=NOMO2 PE=1 SV=1	4	4	1	1
Q12769	Nuclear pore complex protein Nup160 OS=Homo sapiens OX=9606 GN=NUP160 PE=1 SV=3	4	4	0	0
O00471	Exocyst complex component 5 OS=Homo sapiens OX=9606 GN=EXOC5 PE=1 SV=1	4	4	2	2
O95140	Mitofusin-2 OS=Homo sapiens OX=9606 GN=MFN2 PE=1 SV=3	4	3	0	0
O00139	Kinesin-like protein KIF2A OS=Homo sapiens OX=9606 GN=KIF2A PE=1 SV=3	4	4	1	1
Q7RTR8	Taste receptor type 2 member 42 OS=Homo sapiens OX=9606 GN=TAS2R42 PE=2 SV=1	4	1	0	0
Q6P996	Pyridoxal-dependent decarboxylase domain-containing protein 1 OS=Homo sapiens OX=9606 GN=PDXDC1 PE=1 SV=2	4	4	0	0
Q8TCJ2	Dolichyl-diphosphooligosaccharide--protein glycosyltransferase subunit STT3B OS=Homo sapiens OX=9606 GN=STT3B PE=1 SV=1	4	3	0	0
Q969X5	Endoplasmic reticulum-Golgi intermediate compartment protein 1 OS=Homo sapiens OX=9606 GN=ERGIC1 PE=1 SV=1	4	3	2	2
Q86X52	Chondroitin sulfate synthase 1 OS=Homo sapiens OX=9606 GN=CHSY1 PE=1 SV=3	4	4	2	2
Q9Y5X1	Sorting nexin-9 OS=Homo sapiens OX=9606 GN=SNX9 PE=1 SV=1	4	3	0	0
Q9BTY2	Plasma alpha-L-fucosidase OS=Homo sapiens OX=9606 GN=FUCA2 PE=1 SV=2	4	4	0	0
P05412	Transcription factor AP-1 OS=Homo sapiens OX=9606 GN=JUN PE=1 SV=2	4	3	0	0
Q9P289	Serine/threonine-protein kinase 26 OS=Homo sapiens OX=9606 GN=STK26 PE=1 SV=2	4	1	0	0

Q96PU8	Protein quaking OS=Homo sapiens OX=9606 GN=QKI PE=1 SV=1	4	4	0	0
P00505	Aspartate aminotransferase, mitochondrial OS=Homo sapiens OX=9606 GN=GOT2 PE=1 SV=3	4	4	0	0
Q9Y5M8	Signal recognition particle receptor subunit beta OS=Homo sapiens OX=9606 GN=SRPRB PE=1 SV=3	4	3	2	2
Q15629	Translocating chain-associated membrane protein 1 OS=Homo sapiens OX=9606 GN=TRAM1 PE=1 SV=3	4	4	2	1
Q9Y6C9	Mitochondrial carrier homolog 2 OS=Homo sapiens OX=9606 GN=MTCH2 PE=1 SV=1	4	3	0	0
A3KMH1	von Willebrand factor A domain-containing protein 8 OS=Homo sapiens OX=9606 GN=VWA8 PE=1 SV=2	4	4	1	1
Q96N66	Lysophospholipid acyltransferase 7 OS=Homo sapiens OX=9606 GN=MBOAT7 PE=1 SV=2	4	4	1	1
P58557	Endoribonuclease YbeY OS=Homo sapiens OX=9606 GN=YBEY PE=1 SV=2	4	1	1	1
Q13247	Serine/arginine-rich splicing factor 6 OS=Homo sapiens OX=9606 GN=SRSF6 PE=1 SV=2	4	3	1	1
Q86YV9	Hermansky-Pudlak syndrome 6 protein OS=Homo sapiens OX=9606 GN=HPS6 PE=1 SV=1	4	4	0	0
Q9Y4C2	TRPM8 channel-associated factor 1 OS=Homo sapiens OX=9606 GN=TCAF1 PE=1 SV=3	4	3	0	0
Q53R41	FAST kinase domain-containing protein 1, mitochondrial OS=Homo sapiens OX=9606 GN=FASTKD1 PE=1 SV=1	4	4	0	0
Q01804	OTU domain-containing protein 4 OS=Homo sapiens OX=9606 GN=OTUD4 PE=1 SV=4	4	4	1	1
O75390	Citrate synthase, mitochondrial OS=Homo sapiens OX=9606 GN=CS PE=1 SV=2	4	4	0	0
Q9Y3F4	Serine-threonine kinase receptor-associated protein OS=Homo sapiens OX=9606 GN=STRAP PE=1 SV=1	4	4	0	0
A8MV65	Transcription cofactor vestigial-like protein 3 OS=Homo sapiens OX=9606 GN=VGLL3 PE=1 SV=1	4	2	1	1
Q8NBZ7	UDP-glucuronic acid decarboxylase 1 OS=Homo sapiens OX=9606 GN=UXS1 PE=1 SV=1	4	4	1	1
Q93062	RNA-binding protein with multiple splicing OS=Homo sapiens OX=9606 GN=RBPMS PE=1 SV=1	4	2	0	0
Q9NRK6	ATP-binding cassette sub-family B member 10, mitochondrial OS=Homo sapiens OX=9606 GN=ABCB10 PE=1 SV=2	4	3	2	2
Q5PRF9	Protein Smaug homolog 2 OS=Homo sapiens OX=9606 GN=SAMD4B PE=1 SV=1	4	3	0	0
Q13363	C-terminal-binding protein 1 OS=Homo sapiens OX=9606 GN=CTBP1 PE=1 SV=2	4	4	0	0
Q86TW2	Uncharacterized aarF domain-containing protein kinase 1 OS=Homo sapiens OX=9606 GN=ADCK1 PE=2 SV=2	4	4	0	0
Q5JVF3	PCI domain-containing protein 2 OS=Homo sapiens OX=9606 GN=PCID2 PE=1 SV=2	4	3	0	0
P17481	Homeobox protein Hox-B8 OS=Homo sapiens OX=9606 GN=HOXB8 PE=2 SV=2	4	1	0	0
P56589	Peroxisomal biogenesis factor 3 OS=Homo sapiens OX=9606 GN=PEX3 PE=1 SV=1	4	4	1	1
P62258	14-3-3 protein epsilon OS=Homo sapiens OX=9606 GN=YWHAE PE=1 SV=1	4	2	2	2
Q9H6H4	Receptor expression-enhancing protein 4 OS=Homo sapiens OX=9606 GN=REEP4 PE=1 SV=1	4	1	2	2
Q9NUQ6	SPATS2-like protein OS=Homo sapiens OX=9606 GN=SPATS2L PE=1 SV=2	4	4	1	1
Q16637	Survival motor neuron protein OS=Homo sapiens OX=9606 GN=SMN1 PE=1 SV=1	4	4	0	0
P49585	Choline-phosphate cytidylyltransferase A OS=Homo sapiens OX=9606 GN=PCYT1A PE=1 SV=2	4	4	0	0
Q86SK9	Stearoyl-CoA desaturase 5 OS=Homo sapiens OX=9606 GN=SCD5 PE=1 SV=2	4	2	1	1
P27348	14-3-3 protein theta OS=Homo sapiens OX=9606 GN=YWHAQ PE=1 SV=1	4	2	1	1
Q8IUH5	Palmitoyltransferase ZDHHC17 OS=Homo sapiens OX=9606 GN=ZDHHC17 PE=1 SV=2	4	4	0	0
Q96BQ5	Coiled-coil domain-containing protein 127 OS=Homo sapiens OX=9606 GN=CCDC127 PE=1 SV=1	4	3	1	1
Q13751	Laminin subunit beta-3 OS=Homo sapiens OX=9606 GN=LAMB3 PE=1 SV=1	4	4	1	1
Q8NF37	Lysophosphatidylcholine acyltransferase 1 OS=Homo sapiens OX=9606 GN=LPCAT1 PE=1 SV=2	4	4	1	1
Q9UI12	V-type proton ATPase subunit H OS=Homo sapiens OX=9606 GN=ATP6V1H PE=1 SV=1	4	4	0	0
P35249	Replication factor C subunit 4 OS=Homo sapiens OX=9606 GN=RFC4 PE=1 SV=2	4	2	2	2
Q96KP1	Exocyst complex component 2 OS=Homo sapiens OX=9606 GN=EXOC2 PE=1 SV=1	4	4	0	0
P50213	Isocitrate dehydrogenase [NAD] subunit alpha, mitochondrial OS=Homo sapiens OX=9606 GN=IDH3A PE=1 SV=1	4	4	0	0
O43427	Acidic fibroblast growth factor intracellular-binding protein OS=Homo sapiens OX=9606 GN=FIBP PE=1 SV=3	4	2	0	0
Q9BQ67	Glutamate-rich WD repeat-containing protein 1 OS=Homo sapiens OX=9606 GN=GRWD1 PE=1 SV=1	4	3	0	0
P53985	Monocarboxylate transporter 1 OS=Homo sapiens OX=9606 GN=SLC16A1 PE=1 SV=3	4	3	2	2
P35251	Replication factor C subunit 1 OS=Homo sapiens OX=9606 GN=RFC1 PE=1 SV=4	4	4	1	1
Q92973	Transportin-1 OS=Homo sapiens OX=9606 GN=TNPO1 PE=1 SV=2	4	4	2	2
Q9Y277	Voltage-dependent anion-selective channel protein 3 OS=Homo sapiens OX=9606 GN=VDAC3 PE=1 SV=1	4	2	0	0
Q70CQ3	Ubiquitin carboxyl-terminal hydrolase 30 OS=Homo sapiens OX=9606 GN=USP30 PE=1 SV=1	4	4	1	1
O43837	Isocitrate dehydrogenase [NAD] subunit beta, mitochondrial OS=Homo sapiens OX=9606 GN=IDH3B PE=1 SV=2	4	4	2	2
P31483	Nucleolysin TIA-1 isoform p40 OS=Homo sapiens OX=9606 GN=TIA1 PE=1 SV=3	4	1	0	0
P33981	Dual specificity protein kinase TTK OS=Homo sapiens OX=9606 GN=TTK PE=1 SV=2	4	3	2	2
O60830	Mitochondrial import inner membrane translocase subunit Tim17-B OS=Homo sapiens OX=9606 GN=TIMM17B PE=1 SV=1	4	2	1	1
Q9BZX2	Uridine-cytidine kinase 2 OS=Homo sapiens OX=9606 GN=UCK2 PE=1 SV=1	4	4	0	0
O14548	Cytochrome c oxidase subunit 7A-related protein, mitochondrial OS=Homo sapiens OX=9606 GN=COX7A2L PE=1 SV=2	3	3	1	1
Q9Y5S9	RNA-binding protein 8A OS=Homo sapiens OX=9606 GN=RBM8A PE=1 SV=1	3	2	0	0
Q2NL68	Proline and serine-rich protein 3 OS=Homo sapiens OX=9606 GN=PROSER3 PE=2 SV=1	3	2	0	0
P78395	Melanoma antigen preferentially expressed in tumors OS=Homo sapiens OX=9606 GN=PRAME PE=1 SV=1	3	3	1	1

O43808	Peroxisomal membrane protein PMP34 OS=Homo sapiens OX=9606 GN=SLC25A17 PE=1 SV=1	3	3	0	0
Q8N1N4	Keratin, type II cytoskeletal 78 OS=Homo sapiens OX=9606 GN=KRT78 PE=1 SV=2	3	2	0	0
P30530	Tyrosine-protein kinase receptor UFO OS=Homo sapiens OX=9606 GN=AXL PE=1 SV=4	3	3	0	0
Q8N766	ER membrane protein complex subunit 1 OS=Homo sapiens OX=9606 GN=EMC1 PE=1 SV=1	3	3	1	1
Q8NEB9	Phosphatidylinositol 3-kinase catalytic subunit type 3 OS=Homo sapiens OX=9606 GN=PIK3C3 PE=1 SV=1	3	3	0	0
P01111	GTPase NRas OS=Homo sapiens OX=9606 GN=NRAS PE=1 SV=1	3	1	0	0
Q9HC52	Chromobox protein homolog 8 OS=Homo sapiens OX=9606 GN=CBX8 PE=1 SV=3	3	3	1	1
Q13595	Transformer-2 protein homolog alpha OS=Homo sapiens OX=9606 GN=TRA2A PE=1 SV=1	3	1	0	0
Q92791	Endoplasmic reticulum protein SC65 OS=Homo sapiens OX=9606 GN=P3H4 PE=1 SV=1	3	3	0	0
P17275	Transcription factor jun-B OS=Homo sapiens OX=9606 GN=JUNB PE=1 SV=1	3	3	0	0
O75175	CCR4-NOT transcription complex subunit 3 OS=Homo sapiens OX=9606 GN=CNOT3 PE=1 SV=1	3	3	0	0
Q9BW60	Elongation of very long chain fatty acids protein 1 OS=Homo sapiens OX=9606 GN=ELOVL1 PE=1 SV=1	3	1	1	1
Q5BJD5	Transmembrane protein 41B OS=Homo sapiens OX=9606 GN=TMEM41B PE=1 SV=1	3	2	1	1
Q9NSE4	Isoleucine--tRNA ligase, mitochondrial OS=Homo sapiens OX=9606 GN=IARS2 PE=1 SV=2	3	3	0	0
O14735	CDP-diacylglycerol--inositol 3-phosphatidyltransferase OS=Homo sapiens OX=9606 GN=CDIPT PE=1 SV=1	3	2	1	1
Q86Y13	E3 ubiquitin-protein ligase DZIP3 OS=Homo sapiens OX=9606 GN=DZIP3 PE=1 SV=2	3	2	0	0
P41091	Eukaryotic translation initiation factor 2 subunit 3 OS=Homo sapiens OX=9606 GN=EIF2S3 PE=1 SV=3	3	3	1	1
Q03519	Antigen peptide transporter 2 OS=Homo sapiens OX=9606 GN=TAP2 PE=1 SV=1	3	2	0	0
P83881	60S ribosomal protein L36a OS=Homo sapiens OX=9606 GN=RPL36A PE=1 SV=2	3	1	0	0
Q96BY9	Store-operated calcium entry-associated regulatory factor OS=Homo sapiens OX=9606 GN=SARAF PE=1 SV=1	3	2	0	0
Q9UPT5	Exocyst complex component 7 OS=Homo sapiens OX=9606 GN=EXOC7 PE=1 SV=3	3	3	1	1
Q9UHI6	Probable ATP-dependent RNA helicase DDX20 OS=Homo sapiens OX=9606 GN=DDX20 PE=1 SV=2	3	3	1	1
P26196	Probable ATP-dependent RNA helicase DDX6 OS=Homo sapiens OX=9606 GN=DDX6 PE=1 SV=2	3	3	1	1
Q9BPX5	Actin-related protein 2/3 complex subunit 5-like protein OS=Homo sapiens OX=9606 GN=ARPC5L PE=1 SV=1	3	2	1	1
Q13243	Serine/arginine-rich splicing factor 5 OS=Homo sapiens OX=9606 GN=SRSF5 PE=1 SV=1	3	2	0	0
P0CG40	Transcription factor Sp9 OS=Homo sapiens OX=9606 GN=SP9 PE=3 SV=1	3	2	0	0
Q9NPI8	Fanconi anemia group F protein OS=Homo sapiens OX=9606 GN=FANCF PE=1 SV=1	3	3	0	0
P51648	Fatty aldehyde dehydrogenase OS=Homo sapiens OX=9606 GN=ALDH3A2 PE=1 SV=1	3	2	1	1
P04004	Vitronectin OS=Homo sapiens OX=9606 GN=VTN PE=1 SV=1	3	3	1	1
Q9H7E2	Tudor domain-containing protein 3 OS=Homo sapiens OX=9606 GN=TDRD3 PE=1 SV=1	3	3	0	0
B7ZAQ6	Golgi pH regulator A OS=Homo sapiens OX=9606 GN=GPR89A PE=1 SV=2	3	3	1	1
P09234	U1 small nuclear ribonucleoprotein C OS=Homo sapiens OX=9606 GN=SNRPC PE=1 SV=1	3	1	0	0
O75874	Isocitrate dehydrogenase [NADP] cytoplasmic OS=Homo sapiens OX=9606 GN=IDH1 PE=1 SV=2	3	3	0	0
Q96AG3	Solute carrier family 25 member 46 OS=Homo sapiens OX=9606 GN=SLC25A46 PE=1 SV=1	3	3	0	0
P47895	Aldehyde dehydrogenase family 1 member A3 OS=Homo sapiens OX=9606 GN=ALDH1A3 PE=1 SV=2	3	1	0	0
Q9BT40	Inositol polyphosphate 5-phosphatase K OS=Homo sapiens OX=9606 GN=INPP5K PE=1 SV=3	3	3	0	0
Q96P70	Importin-9 OS=Homo sapiens OX=9606 GN=IPO9 PE=1 SV=3	3	3	0	0
Q6ZMG9	Ceramide synthase 6 OS=Homo sapiens OX=9606 GN=CERS6 PE=1 SV=1	3	2	0	0
Q9BQ48	39S ribosomal protein L34, mitochondrial OS=Homo sapiens OX=9606 GN=MRPL34 PE=1 SV=1	3	2	1	1
Q96A72	Protein mago nashi homolog 2 OS=Homo sapiens OX=9606 GN=MAGOHB PE=1 SV=1	3	1	0	0
O95816	BAG family molecular chaperone regulator 2 OS=Homo sapiens OX=9606 GN=BAG2 PE=1 SV=1	3	3	1	1
Q8IXI2	Mitochondrial Rho GTPase 1 OS=Homo sapiens OX=9606 GN=RHOT1 PE=1 SV=2	3	2	0	0
P34897	Serine hydroxymethyltransferase, mitochondrial OS=Homo sapiens OX=9606 GN=SHMT2 PE=1 SV=3	3	3	0	0
Q9UBD5	Origin recognition complex subunit 3 OS=Homo sapiens OX=9606 GN=ORC3 PE=1 SV=1	3	2	0	0
Q9UDR5	Alpha-aminoadipic semialdehyde synthase, mitochondrial OS=Homo sapiens OX=9606 GN=AASS PE=1 SV=1	3	3	0	0
O75494	Serine/arginine-rich splicing factor 10 OS=Homo sapiens OX=9606 GN=SRSF10 PE=1 SV=1	3	3	1	1
O75821	Eukaryotic translation initiation factor 3 subunit G OS=Homo sapiens OX=9606 GN=EIF3G PE=1 SV=2	3	3	0	0
Q13608	Peroxisome assembly factor 2 OS=Homo sapiens OX=9606 GN=PEX6 PE=1 SV=2	3	3	0	0
Q75QN2	Integrator complex subunit 8 OS=Homo sapiens OX=9606 GN=INTS8 PE=1 SV=1	3	2	1	1
O60568	Procollagen-lysine,2-oxoglutarate 5-dioxygenase 3 OS=Homo sapiens OX=9606 GN=PLOD3 PE=1 SV=1	3	3	0	0
Q9BZQ6	ER degradation-enhancing alpha-mannosidase-like protein 3 OS=Homo sapiens OX=9606 GN=EDEM3 PE=1 SV=2	3	3	0	0
O60506	Heterogeneous nuclear ribonucleoprotein Q OS=Homo sapiens OX=9606 GN=SYNCRIP PE=1 SV=2	3	1	0	0
P10588	Nuclear receptor subfamily 2 group F member 6 OS=Homo sapiens OX=9606 GN=NR2F6 PE=1 SV=2	3	3	0	0
O43747	AP-1 complex subunit gamma-1 OS=Homo sapiens OX=9606 GN=AP1G1 PE=1 SV=5	3	3	1	1
P18031	Tyrosine-protein phosphatase non-receptor type 1 OS=Homo sapiens OX=9606 GN=PTPN1 PE=1 SV=1	3	3	0	0
O14613	Cdc42 effector protein 2 OS=Homo sapiens OX=9606 GN=CDC42EP2 PE=1 SV=1	3	3	0	0

Q9H0B3	IQ domain-containing protein N OS=Homo sapiens OX=9606 GN=IQCN PE=1 SV=1	3	1	0	0
O00148	ATP-dependent RNA helicase DDX39A OS=Homo sapiens OX=9606 GN=DDX39A PE=1 SV=2	3	3	0	0
Q9NWR8	Calcium uniporter regulatory subunit MCUb, mitochondrial OS=Homo sapiens OX=9606 GN=MCUB PE=1 SV=2	3	3	0	0
Q9Y3E5	Peptidyl-tRNA hydrolase 2, mitochondrial OS=Homo sapiens OX=9606 GN=PTRH2 PE=1 SV=1	3	2	0	0
Q9H0S4	Probable ATP-dependent RNA helicase DDX47 OS=Homo sapiens OX=9606 GN=DDX47 PE=1 SV=1	3	3	1	1
Q92643	GPI-anchor transamidase OS=Homo sapiens OX=9606 GN=PIGK PE=1 SV=2	3	3	0	0
P45954	Short/branched chain specific acyl-CoA dehydrogenase, mitochondrial OS=Homo sapiens OX=9606 GN=ACADSB PE=1 SV=1	3	3	0	0
Q96T37	RNA-binding protein 15 OS=Homo sapiens OX=9606 GN=RBM15 PE=1 SV=2	3	2	0	0
Q99873	Protein arginine N-methyltransferase 1 OS=Homo sapiens OX=9606 GN=PRMT1 PE=1 SV=3	3	3	0	0
P37198	Nuclear pore glycoprotein p62 OS=Homo sapiens OX=9606 GN=NUP62 PE=1 SV=3	3	2	0	0
Q9H8W3	Protein FAM204A OS=Homo sapiens OX=9606 GN=FAM204A PE=1 SV=1	3	3	0	0
Q96ST3	Paired amphipathic helix protein Sin3a OS=Homo sapiens OX=9606 GN=SIN3A PE=1 SV=2	3	3	0	0
Q8NFH3	Nucleoporin Nup43 OS=Homo sapiens OX=9606 GN=NUP43 PE=1 SV=1	3	2	0	0
Q8TDX7	Serine/threonine-protein kinase Nek7 OS=Homo sapiens OX=9606 GN=NEK7 PE=1 SV=1	3	3	0	0
Q96KR1	Zinc finger RNA-binding protein OS=Homo sapiens OX=9606 GN=ZFR PE=1 SV=2	3	3	0	0
Q15293	Reticulocalbin-1 OS=Homo sapiens OX=9606 GN=RCN1 PE=1 SV=1	3	3	0	0
Q53GS7	Nucleoporin GLE1 OS=Homo sapiens OX=9606 GN=GLE1 PE=1 SV=2	3	3	0	0
Q8NFW8	N-acylneuraminate cytidylyltransferase OS=Homo sapiens OX=9606 GN=CMAS PE=1 SV=2	3	2	0	0
P29692	Elongation factor 1-delta OS=Homo sapiens OX=9606 GN=EEF1D PE=1 SV=5	3	3	1	1
Q9UPR3	Protein SMG5 OS=Homo sapiens OX=9606 GN=SMG5 PE=1 SV=3	3	3	0	0
O43292	Glycosylphosphatidylinositol anchor attachment 1 protein OS=Homo sapiens OX=9606 GN=GPAA1 PE=1 SV=3	3	3	0	0
P83436	Conserved oligomeric Golgi complex subunit 7 OS=Homo sapiens OX=9606 GN=COG7 PE=1 SV=1	2	2	0	0
O75165	DnaJ homolog subfamily C member 13 OS=Homo sapiens OX=9606 GN=DNAJC13 PE=1 SV=5	2	2	0	0
Q8WTW3	Conserved oligomeric Golgi complex subunit 1 OS=Homo sapiens OX=9606 GN=COG1 PE=1 SV=1	2	2	0	0
Q9UQ53	Alpha-1,3-mannosyl-glycoprotein 4-beta-N-acetylglucosaminyltransferase B OS=Homo sapiens OX=9606 GN=MGAT4B PE=1 SV=1	2	2	0	0
O14966	Ras-related protein Rab-7L1 OS=Homo sapiens OX=9606 GN=RAB29 PE=1 SV=1	2	1	0	0
Q96D15	Reticulocalbin-3 OS=Homo sapiens OX=9606 GN=RCN3 PE=1 SV=1	2	2	0	0
Q9BSR8	Protein YIPF4 OS=Homo sapiens OX=9606 GN=YIPF4 PE=1 SV=1	2	2	0	0
Q969P6	DNA topoisomerase I, mitochondrial OS=Homo sapiens OX=9606 GN=TOP1MT PE=1 SV=1	2	1	0	0
P26640	Valine--tRNA ligase OS=Homo sapiens OX=9606 GN=VARS PE=1 SV=4	2	2	1	1
Q86TG7	Retrotransposon-derived protein PEG10 OS=Homo sapiens OX=9606 GN=PEG10 PE=1 SV=2	2	2	0	0
Q8TAM2	Tetratricopeptide repeat protein 8 OS=Homo sapiens OX=9606 GN=TTC8 PE=1 SV=2	2	2	0	0
Q15006	ER membrane protein complex subunit 2 OS=Homo sapiens OX=9606 GN=EMC2 PE=1 SV=1	2	2	0	0
Q9P2K5	Myelin expression factor 2 OS=Homo sapiens OX=9606 GN=MYEF2 PE=1 SV=3	2	2	1	1
O94955	Rho-related BTB domain-containing protein 3 OS=Homo sapiens OX=9606 GN=RHOBTB3 PE=1 SV=2	2	2	1	1
Q969M3	Protein YIPF5 OS=Homo sapiens OX=9606 GN=YIPF5 PE=1 SV=1	2	2	0	0
Q96MU7	YTH domain-containing protein 1 OS=Homo sapiens OX=9606 GN=YTHDC1 PE=1 SV=3	2	2	0	0
P21817	Ryanodine receptor 1 OS=Homo sapiens OX=9606 GN=RYR1 PE=1 SV=3	2	2	0	0
Q8N357	Solute carrier family 35 member F6 OS=Homo sapiens OX=9606 GN=SLC35F6 PE=1 SV=1	2	1	0	0
Q16394	Exostosin-1 OS=Homo sapiens OX=9606 GN=EXT1 PE=1 SV=2	2	2	1	1
Q3SXM5	Inactive hydroxysteroid dehydrogenase-like protein 1 OS=Homo sapiens OX=9606 GN=HSDL1 PE=1 SV=3	2	2	0	0
Q15084	Protein disulfide-isomerase A6 OS=Homo sapiens OX=9606 GN=PDIA6 PE=1 SV=1	2	2	1	1
Q96MW5	Conserved oligomeric Golgi complex subunit 8 OS=Homo sapiens OX=9606 GN=COG8 PE=1 SV=2	2	2	0	0
O43414	ERI1 exoribonuclease 3 OS=Homo sapiens OX=9606 GN=ERI3 PE=1 SV=2	2	2	1	1
Q6NUM9	All-trans-retinol 13,14-reductase OS=Homo sapiens OX=9606 GN=RETSAT PE=1 SV=2	2	2	0	0
P54886	Delta-1-pyrroline-5-carboxylate synthase OS=Homo sapiens OX=9606 GN=ALDH18A1 PE=1 SV=2	2	2	1	1
O75569	Interferon-inducible double-stranded RNA-dependent protein kinase activator A OS=Homo sapiens OX=9606 GN=PRKRA PE=1 SV=1	2	2	0	0
Q9GZP9	Derlin-2 OS=Homo sapiens OX=9606 GN=DERL2 PE=1 SV=1	2	1	0	0
Q15645	Pachytene checkpoint protein 2 homolog OS=Homo sapiens OX=9606 GN=TRIP13 PE=1 SV=2	2	2	0	0
Q6ZRP7	Sulfhydryl oxidase 2 OS=Homo sapiens OX=9606 GN=QSOX2 PE=1 SV=3	2	2	0	0
O14744	Protein arginine N-methyltransferase 5 OS=Homo sapiens OX=9606 GN=PRMT5 PE=1 SV=4	2	2	0	0
Q9NTJ3	Structural maintenance of chromosomes protein 4 OS=Homo sapiens OX=9606 GN=SMC4 PE=1 SV=2	2	2	1	1
Q9Y240	C-type lectin domain family 11 member A OS=Homo sapiens OX=9606 GN=CLEC11A PE=1 SV=1	2	2	0	0
Q9HCS7	Pre-mRNA-splicing factor SYF1 OS=Homo sapiens OX=9606 GN=XAB2 PE=1 SV=2	2	2	0	0
B1AK76	Putative SNURF-like protein OS=Homo sapiens OX=9606 GN=SNURFL PE=5 SV=2	2	1	0	0
Q14145	Kelch-like ECH-associated protein 1 OS=Homo sapiens OX=9606 GN=KEAP1 PE=1 SV=2	2	2	0	0

O75063	Glycosaminoglycan xylosylkinase OS=Homo sapiens OX=9606 GN=FAM20B PE=1 SV=1	2	2	0	0
Q16850	Lanosterol 14-alpha demethylase OS=Homo sapiens OX=9606 GN=CYP51A1 PE=1 SV=3	2	2	0	0
Q14154	Death ligand signal enhancer OS=Homo sapiens OX=9606 GN=KIAA0141 PE=1 SV=3	2	2	1	1
Q96CB8	Integrator complex subunit 12 OS=Homo sapiens OX=9606 GN=INTS12 PE=1 SV=1	2	2	0	0
Q16594	Transcription initiation factor TFIID subunit 9 OS=Homo sapiens OX=9606 GN=TAF9 PE=1 SV=1	2	2	1	1
Q9NX61	Transmembrane protein 161A OS=Homo sapiens OX=9606 GN=TMEM161A PE=1 SV=1	2	2	0	0
O75794	Cell division cycle protein 123 homolog OS=Homo sapiens OX=9606 GN=CDC123 PE=1 SV=1	2	2	0	0
Q96RQ1	Endoplasmic reticulum-Golgi intermediate compartment protein 2 OS=Homo sapiens OX=9606 GN=ERGIC2 PE=1 SV=2	2	2	0	0
Q71F23	Centromere protein U OS=Homo sapiens OX=9606 GN=CENPU PE=1 SV=1	2	2	1	1
O75787	Renin receptor OS=Homo sapiens OX=9606 GN=ATP6AP2 PE=1 SV=2	2	2	0	0
Q96EP5	DAZ-associated protein 1 OS=Homo sapiens OX=9606 GN=DAZAP1 PE=1 SV=1	2	1	1	1
Q15800	Methylsterol monooxygenase 1 OS=Homo sapiens OX=9606 GN=MSMO1 PE=1 SV=1	2	2	0	0
Q8NBL1	Protein O-glucosyltransferase 1 OS=Homo sapiens OX=9606 GN=POGLUT1 PE=1 SV=1	2	2	0	0
P42357	Histidine ammonia-lyase OS=Homo sapiens OX=9606 GN=HAL PE=1 SV=1	2	1	0	0
Q92520	Protein FAM3C OS=Homo sapiens OX=9606 GN=FAM3C PE=1 SV=1	2	2	1	1
P08134	Rho-related GTP-binding protein RhoC OS=Homo sapiens OX=9606 GN=RHOC PE=1 SV=1	2	2	0	0
O15397	Importin-8 OS=Homo sapiens OX=9606 GN=IPO8 PE=1 SV=2	2	2	0	0
Q9BQS8	FYVE and coiled-coil domain-containing protein 1 OS=Homo sapiens OX=9606 GN=FYCO1 PE=1 SV=3	2	1	0	0
Q99958	Forkhead box protein C2 OS=Homo sapiens OX=9606 GN=FOXC2 PE=1 SV=1	2	2	1	1
Q9Y5Z9	UbiA prenyltransferase domain-containing protein 1 OS=Homo sapiens OX=9606 GN=UBIAD1 PE=1 SV=1	2	2	1	1
Q9H0H0	Integrator complex subunit 2 OS=Homo sapiens OX=9606 GN=INTS2 PE=1 SV=2	2	2	0	0
P62888	60S ribosomal protein L30 OS=Homo sapiens OX=9606 GN=RPL30 PE=1 SV=2	2	2	0	0
Q9Y5L0	Transportin-3 OS=Homo sapiens OX=9606 GN=TNPO3 PE=1 SV=3	2	2	0	0
Q8IUX1	Complex I assembly factor TMEM126B, mitochondrial OS=Homo sapiens OX=9606 GN=TMEM126B PE=1 SV=2	2	2	0	0
Q9UKV5	E3 ubiquitin-protein ligase AMFR OS=Homo sapiens OX=9606 GN=AMFR PE=1 SV=2	2	2	0	0
Q9NZN8	CCR4-NOT transcription complex subunit 2 OS=Homo sapiens OX=9606 GN=CNOT2 PE=1 SV=1	2	2	1	1
Q9ULK4	Mediator of RNA polymerase II transcription subunit 23 OS=Homo sapiens OX=9606 GN=MED23 PE=1 SV=2	2	2	0	0
Q96QR8	Transcriptional activator protein Pur-beta OS=Homo sapiens OX=9606 GN=PURB PE=1 SV=3	2	1	0	0
Q5VZE5	N-alpha-acetyltransferase 35, NatC auxiliary subunit OS=Homo sapiens OX=9606 GN=NAA35 PE=1 SV=1	2	2	0	0
Q8NBJ5	Procollagen galactosyltransferase 1 OS=Homo sapiens OX=9606 GN=COLGALT1 PE=1 SV=1	2	2	1	1
Q9H649	tRNA (cytosine(34)-C(5))-methyltransferase, mitochondrial OS=Homo sapiens OX=9606 GN=NSUN3 PE=1 SV=1	2	2	0	0
Q99661	Kinesin-like protein KIF2C OS=Homo sapiens OX=9606 GN=KIF2C PE=1 SV=2	2	2	0	0
Q96HR8	H/ACA ribonucleoprotein complex non-core subunit NAF1 OS=Homo sapiens OX=9606 GN=NAF1 PE=1 SV=2	2	1	1	1
O00623	Peroxisome assembly protein 12 OS=Homo sapiens OX=9606 GN=PEX12 PE=1 SV=1	2	2	0	0
Q8TCG1	Protein CIP2A OS=Homo sapiens OX=9606 GN=CIP2A PE=1 SV=2	2	2	0	0
Q96BI1	Solute carrier family 22 member 18 OS=Homo sapiens OX=9606 GN=SLC22A18 PE=1 SV=3	2	2	0	0
Q8TC12	Retinol dehydrogenase 11 OS=Homo sapiens OX=9606 GN=RDH11 PE=1 SV=2	2	2	1	1
O60701	UDP-glucose 6-dehydrogenase OS=Homo sapiens OX=9606 GN=UGDH PE=1 SV=1	2	2	1	1
Q92908	Transcription factor GATA-6 OS=Homo sapiens OX=9606 GN=GATA6 PE=1 SV=2	2	1	1	1
Q9NWB6	Arginine and glutamate-rich protein 1 OS=Homo sapiens OX=9606 GN=ARGLU1 PE=1 SV=1	2	2	1	1
Q3MHD2	Protein LSM12 homolog OS=Homo sapiens OX=9606 GN=LSM12 PE=1 SV=2	2	2	0	0
Q6ZT21	Transmembrane protein with metallophosphoesterase domain OS=Homo sapiens OX=9606 GN=TMPPE PE=2 SV=2	2	2	0	0
Q14258	E3 ubiquitin/ISG15 ligase TRIM25 OS=Homo sapiens OX=9606 GN=TRIM25 PE=1 SV=2	2	2	0	0
Q12874	Splicing factor 3A subunit 3 OS=Homo sapiens OX=9606 GN=SF3A3 PE=1 SV=1	2	2	1	1
Q9UBB4	Ataxin-10 OS=Homo sapiens OX=9606 GN=ATXN10 PE=1 SV=1	2	2	0	0
Q15031	Probable leucine--tRNA ligase, mitochondrial OS=Homo sapiens OX=9606 GN=LARS2 PE=1 SV=2	2	2	0	0
Q14746	Conserved oligomeric Golgi complex subunit 2 OS=Homo sapiens OX=9606 GN=COG2 PE=1 SV=1	2	2	0	0
Q86WK9	Membrane progestin receptor alpha OS=Homo sapiens OX=9606 GN=PAQR7 PE=2 SV=1	2	2	0	0
Q8NFH5	Nucleoporin NUP35 OS=Homo sapiens OX=9606 GN=NUP35 PE=1 SV=1	2	2	0	0
O60645	Exocyst complex component 3 OS=Homo sapiens OX=9606 GN=EXOC3 PE=1 SV=2	2	2	0	0
Q8NEW0	Zinc transporter 7 OS=Homo sapiens OX=9606 GN=SLC30A7 PE=2 SV=1	2	2	0	0
P69905	Hemoglobin subunit alpha OS=Homo sapiens OX=9606 GN=HBA1 PE=1 SV=2	2	2	0	0
Q16718	NADH dehydrogenase [ubiquinone] 1 alpha subcomplex subunit 5 OS=Homo sapiens OX=9606 GN=NDUFA5 PE=1 SV=3	2	2	1	1
P53701	Cytochrome c-type heme lyase OS=Homo sapiens OX=9606 GN=HCCS PE=1 SV=1	2	2	0	0
Q6P3X3	Tetratricopeptide repeat protein 27 OS=Homo sapiens OX=9606 GN=TTC27 PE=1 SV=1	2	2	0	0
O15269	Serine palmitoyltransferase 1 OS=Homo sapiens OX=9606 GN=SPTLC1 PE=1 SV=1	2	2	1	1

Q7Z7L1	Schlafen family member 11 OS=Homo sapiens OX=9606 GN=SLFN11 PE=1 SV=2	2	1	0	0
Q99700	Ataxin-2 OS=Homo sapiens OX=9606 GN=ATXN2 PE=1 SV=2	2	2	0	0
Q8N2K0	Monoacylglycerol lipase ABHD12 OS=Homo sapiens OX=9606 GN=ABHD12 PE=1 SV=2	2	2	1	1
Q9HC07	Transmembrane protein 165 OS=Homo sapiens OX=9606 GN=TMEM165 PE=1 SV=1	2	2	0	0
P49761	Dual specificity protein kinase CLK3 OS=Homo sapiens OX=9606 GN=CLK3 PE=1 SV=3	2	2	1	1
Q96RF0	Sorting nexin-18 OS=Homo sapiens OX=9606 GN=SNX18 PE=1 SV=2	2	2	0	0
Q8IUX4	DNA dC->dU-editing enzyme APOBEC-3F OS=Homo sapiens OX=9606 GN=APOBEC3F PE=1 SV=3	2	2	0	0
P18859	ATP synthase-coupling factor 6, mitochondrial OS=Homo sapiens OX=9606 GN=ATP5J PE=1 SV=1	2	2	0	0
Q05932	Folylpolyglutamate synthase, mitochondrial OS=Homo sapiens OX=9606 GN=FPGS PE=1 SV=3	2	2	0	0
Q9BV68	E3 ubiquitin-protein ligase RNF126 OS=Homo sapiens OX=9606 GN=RNF126 PE=1 SV=1	2	2	0	0
Q86WX3	Active regulator of SIRT1 OS=Homo sapiens OX=9606 GN=RPS19BP1 PE=1 SV=1	2	2	0	0
Q13950	Runt-related transcription factor 2 OS=Homo sapiens OX=9606 GN=RUNX2 PE=1 SV=2	2	2	0	0
P39880	Homeobox protein cut-like 1 OS=Homo sapiens OX=9606 GN=CUX1 PE=1 SV=3	2	2	0	0
P24390	ER lumen protein-retaining receptor 1 OS=Homo sapiens OX=9606 GN=KDELR1 PE=1 SV=1	2	1	0	0
Q8IZV5	Retinol dehydrogenase 10 OS=Homo sapiens OX=9606 GN=RDH10 PE=1 SV=1	2	2	1	1
Q13049	E3 ubiquitin-protein ligase TRIM32 OS=Homo sapiens OX=9606 GN=TRIM32 PE=1 SV=2	2	2	0	0
Q96J01	THO complex subunit 3 OS=Homo sapiens OX=9606 GN=THOC3 PE=1 SV=1	2	1	0	0
P49756	RNA-binding protein 25 OS=Homo sapiens OX=9606 GN=RBM25 PE=1 SV=3	2	2	1	1
Q53G59	Kelch-like protein 12 OS=Homo sapiens OX=9606 GN=KLHL12 PE=1 SV=2	2	2	0	0
P05091	Aldehyde dehydrogenase, mitochondrial OS=Homo sapiens OX=9606 GN=ALDH2 PE=1 SV=2	2	1	0	0
Q9Y3D0	Mitotic spindle-associated MMXD complex subunit MIP18 OS=Homo sapiens OX=9606 GN=FAM96B PE=1 SV=1	2	2	0	0
Q9P2R7	Succinate--CoA ligase [ADP-forming] subunit beta, mitochondrial OS=Homo sapiens OX=9606 GN=SUCLA2 PE=1 SV=3	2	2	0	0
Q07352	mRNA decay activator protein ZFP36L1 OS=Homo sapiens OX=9606 GN=ZFP36L1 PE=1 SV=1	2	1	0	0
Q12849	G-rich sequence factor 1 OS=Homo sapiens OX=9606 GN=GRSF1 PE=1 SV=3	2	2	1	1
Q68E01	Integrator complex subunit 3 OS=Homo sapiens OX=9606 GN=INTS3 PE=1 SV=1	2	2	1	1
O75528	Transcriptional adapter 3 OS=Homo sapiens OX=9606 GN=TADA3 PE=1 SV=1	2	2	1	1
P30154	Serine/threonine-protein phosphatase 2A 65 kDa regulatory subunit A beta isoform OS=Homo sapiens OX=9606 GN=PPP2R1B PE=1 SV=3	2	1	0	0
Q8TED1	Probable glutathione peroxidase 8 OS=Homo sapiens OX=9606 GN=GPX8 PE=1 SV=2	2	2	1	1
Q9Y3L5	Ras-related protein Rap-2c OS=Homo sapiens OX=9606 GN=RAP2C PE=1 SV=1	2	1	0	0
Q8IXL6	Extracellular serine/threonine protein kinase FAM20C OS=Homo sapiens OX=9606 GN=FAM20C PE=1 SV=2	2	2	1	1
Q8N9T8	Protein KRI1 homolog OS=Homo sapiens OX=9606 GN=KRI1 PE=1 SV=3	2	2	1	1
Q96EY4	Translation machinery-associated protein 16 OS=Homo sapiens OX=9606 GN=TMA16 PE=1 SV=2	2	2	0	0
Q9UP83	Conserved oligomeric Golgi complex subunit 5 OS=Homo sapiens OX=9606 GN=COG5 PE=1 SV=3	2	2	0	0
Q9Y2V7	Conserved oligomeric Golgi complex subunit 6 OS=Homo sapiens OX=9606 GN=COG6 PE=1 SV=2	2	2	0	0
Q13637	Ras-related protein Rab-32 OS=Homo sapiens OX=9606 GN=RAB32 PE=1 SV=3	2	1	0	0
Q92600	CCR4-NOT transcription complex subunit 9 OS=Homo sapiens OX=9606 GN=CNOT9 PE=1 SV=1	2	2	0	0
Q9H3H9	Transcription elongation factor A protein-like 2 OS=Homo sapiens OX=9606 GN=TCEAL2 PE=2 SV=1	2	1	0	0
Q9H490	Phosphatidylinositol glycan anchor biosynthesis class U protein OS=Homo sapiens OX=9606 GN=PIGU PE=1 SV=3	2	2	1	1
Q9NPC8	Homeobox protein SIX2 OS=Homo sapiens OX=9606 GN=SIX2 PE=1 SV=1	2	2	0	0
P05089	Arginase-1 OS=Homo sapiens OX=9606 GN=ARG1 PE=1 SV=2	2	2	0	0
O75844	CAAX prenyl protease 1 homolog OS=Homo sapiens OX=9606 GN=ZMPSTE24 PE=1 SV=2	2	2	0	0
Q8N159	N-acetylglutamate synthase, mitochondrial OS=Homo sapiens OX=9606 GN=NAGS PE=1 SV=1	2	2	1	1
Q01543	Friend leukemia integration 1 transcription factor OS=Homo sapiens OX=9606 GN=FLI1 PE=1 SV=1	2	2	0	0
P00846	ATP synthase subunit a OS=Homo sapiens OX=9606 GN=MT-ATP6 PE=1 SV=1	2	1	0	0
Q99417	c-Myc-binding protein OS=Homo sapiens OX=9606 GN=MYCBP PE=1 SV=3	2	2	0	0
Q5T0B9	Zinc finger protein 362 OS=Homo sapiens OX=9606 GN=ZNF362 PE=1 SV=1	2	1	0	0
Q15436	Protein transport protein Sec23A OS=Homo sapiens OX=9606 GN=SEC23A PE=1 SV=2	2	1	0	0
Q5BJF2	Sigma intracellular receptor 2 OS=Homo sapiens OX=9606 GN=TMEM97 PE=1 SV=1	2	2	1	1
O00400	Acetyl-coenzyme A transporter 1 OS=Homo sapiens OX=9606 GN=SLC33A1 PE=1 SV=1	2	2	0	0
Q14156	Protein EFR3 homolog A OS=Homo sapiens OX=9606 GN=EFR3A PE=1 SV=2	2	2	0	0
Q9C0E2	Exportin-4 OS=Homo sapiens OX=9606 GN=XPO4 PE=1 SV=2	2	2	0	0
O14880	Microsomal glutathione S-transferase 3 OS=Homo sapiens OX=9606 GN=MGST3 PE=1 SV=1	2	2	1	1
P52789	Hexokinase-2 OS=Homo sapiens OX=9606 GN=HK2 PE=1 SV=2	2	2	0	0
P43686	26S proteasome regulatory subunit 6B OS=Homo sapiens OX=9606 GN=PSMC4 PE=1 SV=2	2	2	0	0
Q3KQZ1	Solute carrier family 25 member 35 OS=Homo sapiens OX=9606 GN=SLC25A35 PE=2 SV=1	2	2	0	0
Q96A65	Exocyst complex component 4 OS=Homo sapiens OX=9606 GN=EXOC4 PE=1 SV=1	2	2	1	1

Q9NX05	Constitutive coactivator of PPAR-gamma-like protein 2 OS=Homo sapiens OX=9606 GN=FAM120C PE=1 SV=3	2	2	0	0
P61421	V-type proton ATPase subunit d 1 OS=Homo sapiens OX=9606 GN=ATP6V0D1 PE=1 SV=1	2	2	0	0
Q9NUQ2	1-acyl-sn-glycerol-3-phosphate acyltransferase epsilon OS=Homo sapiens OX=9606 GN=AGPAT5 PE=1 SV=3	2	2	0	0
P08236	Beta-glucuronidase OS=Homo sapiens OX=9606 GN=GUSB PE=1 SV=2	2	2	0	0
Q8IXZ3	Transcription factor Sp8 OS=Homo sapiens OX=9606 GN=SP8 PE=1 SV=3	2	1	0	0
Q53F19	Nuclear cap-binding protein subunit 3 OS=Homo sapiens OX=9606 GN=NCBP3 PE=1 SV=2	2	2	1	1
O15042	U2 snRNP-associated SURP motif-containing protein OS=Homo sapiens OX=9606 GN=U2SURP PE=1 SV=2	2	2	1	1
O14981	TATA-binding protein-associated factor 172 OS=Homo sapiens OX=9606 GN=BTAF1 PE=1 SV=2	2	2	1	1
Q9H553	Alpha-1,3/1,6-mannosyltransferase ALG2 OS=Homo sapiens OX=9606 GN=ALG2 PE=1 SV=1	2	2	0	0
P10589	COUP transcription factor 1 OS=Homo sapiens OX=9606 GN=NR2F1 PE=1 SV=1	2	2	0	0
Q9BZJ4	Solute carrier family 25 member 39 OS=Homo sapiens OX=9606 GN=SLC25A39 PE=2 SV=2	2	2	0	0
O00712	Nuclear factor 1 B-type OS=Homo sapiens OX=9606 GN=NFIB PE=1 SV=2	1	1	0	0
Q9NQT8	Kinesin-like protein KIF13B OS=Homo sapiens OX=9606 GN=KIF13B PE=1 SV=2	1	1	0	0
Q7Z4Q2	HEAT repeat-containing protein 3 OS=Homo sapiens OX=9606 GN=HEATR3 PE=1 SV=2	1	1	0	0
Q9H0E3	Histone deacetylase complex subunit SAP130 OS=Homo sapiens OX=9606 GN=SAP130 PE=1 SV=1	1	1	0	0
P56378	6.8 kDa mitochondrial proteolipid OS=Homo sapiens OX=9606 GN=MP68 PE=1 SV=1	1	1	0	0
Q14938	Nuclear factor 1 X-type OS=Homo sapiens OX=9606 GN=NFIX PE=1 SV=2	1	1	0	0
Q9BRZ2	E3 ubiquitin-protein ligase TRIM56 OS=Homo sapiens OX=9606 GN=TRIM56 PE=1 SV=3	1	1	0	0
Q6ZNC4	Zinc finger protein 704 OS=Homo sapiens OX=9606 GN=ZNF704 PE=1 SV=1	1	1	0	0
Q9UKM7	Endoplasmic reticulum mannosyl-oligosaccharide 1,2-alpha-mannosidase OS=Homo sapiens OX=9606 GN=MAN1B1 PE=1 SV=2	1	1	0	0
Q6PIW4	Fidgetin-like protein 1 OS=Homo sapiens OX=9606 GN=FIGNL1 PE=1 SV=2	1	1	0	0
Q96TC7	Regulator of microtubule dynamics protein 3 OS=Homo sapiens OX=9606 GN=RMDN3 PE=1 SV=2	1	1	0	0
Q9Y2Z9	Ubiquinone biosynthesis monooxygenase COQ6, mitochondrial OS=Homo sapiens OX=9606 GN=COQ6 PE=1 SV=2	1	1	0	0
O15528	25-hydroxyvitamin D-1 alpha hydroxylase, mitochondrial OS=Homo sapiens OX=9606 GN=CYP27B1 PE=1 SV=1	1	1	0	0
P21964	Catechol O-methyltransferase OS=Homo sapiens OX=9606 GN=COMT PE=1 SV=2	1	1	0	0
Q8N5B7	Ceramide synthase 5 OS=Homo sapiens OX=9606 GN=CERS5 PE=2 SV=1	1	1	0	0
P02788	Lactotransferrin OS=Homo sapiens OX=9606 GN=LTF PE=1 SV=6	1	1	0	0
P09661	U2 small nuclear ribonucleoprotein A' OS=Homo sapiens OX=9606 GN=SNRPA1 PE=1 SV=2	1	1	0	0
O94766	Galactosylgalactosylxylosylprotein 3-beta-glucuronosyltransferase 3 OS=Homo sapiens OX=9606 GN=B3GAT3 PE=1 SV=2	1	1	0	0
O14684	Prostaglandin E synthase OS=Homo sapiens OX=9606 GN=PTGES PE=1 SV=2	1	1	0	0
P18084	Integrin beta-5 OS=Homo sapiens OX=9606 GN=ITGB5 PE=1 SV=1	1	1	0	0
Q15165	Serum paraoxonase/arylesterase 2 OS=Homo sapiens OX=9606 GN=PON2 PE=1 SV=4	1	1	0	0
Q9H1C4	Protein unc-93 homolog B1 OS=Homo sapiens OX=9606 GN=UNC93B1 PE=1 SV=2	1	1	0	0
Q96NZ1	Forkhead box protein N4 OS=Homo sapiens OX=9606 GN=FOXN4 PE=1 SV=2	1	1	0	0
Q8TBE7	Solute carrier family 35 member G2 OS=Homo sapiens OX=9606 GN=SLC35G2 PE=1 SV=3	1	1	0	0
Q9UL54	Serine/threonine-protein kinase TAO2 OS=Homo sapiens OX=9606 GN=TAOK2 PE=1 SV=2	1	1	0	0
P50897	Palmitoyl-protein thioesterase 1 OS=Homo sapiens OX=9606 GN=PPT1 PE=1 SV=1	1	1	0	0
Q8TEY7	Ubiquitin carboxyl-terminal hydrolase 33 OS=Homo sapiens OX=9606 GN=USP33 PE=1 SV=2	1	1	0	0
Q13510	Acid ceramidase OS=Homo sapiens OX=9606 GN=ASAH1 PE=1 SV=5	1	1	0	0
Q6P4E1	Protein CASC4 OS=Homo sapiens OX=9606 GN=CASC4 PE=1 SV=1	1	1	0	0
O60232	Sjoegren syndrome/scleroderma autoantigen 1 OS=Homo sapiens OX=9606 GN=SSSCA1 PE=1 SV=1	1	1	0	0
Q96SB4	SRSF protein kinase 1 OS=Homo sapiens OX=9606 GN=SRPK1 PE=1 SV=2	1	1	0	0
Q6PGP7	Tetratricopeptide repeat protein 37 OS=Homo sapiens OX=9606 GN=TTC37 PE=1 SV=1	1	1	0	0
Q13868	Exosome complex component RRP4 OS=Homo sapiens OX=9606 GN=EXOSC2 PE=1 SV=2	1	1	0	0
Q9NQT4	Exosome complex component RRP46 OS=Homo sapiens OX=9606 GN=EXOSC5 PE=1 SV=1	1	1	0	0
Q9UGQ3	Solute carrier family 2, facilitated glucose transporter member 6 OS=Homo sapiens OX=9606 GN=SLC2A6 PE=1 SV=2	1	1	0	0
Q9Y5S2	Serine/threonine-protein kinase MRCK beta OS=Homo sapiens OX=9606 GN=CDC42BPB PE=1 SV=2	1	1	0	0
Q8TED0	U3 small nucleolar RNA-associated protein 15 homolog OS=Homo sapiens OX=9606 GN=UTP15 PE=1 SV=3	1	1	0	0
O95772	STARD3 N-terminal-like protein OS=Homo sapiens OX=9606 GN=STARD3NL PE=1 SV=1	1	1	0	0
Q9P0T7	Transmembrane protein 9 OS=Homo sapiens OX=9606 GN=TMEM9 PE=1 SV=1	1	1	0	0
P49642	DNA primase small subunit OS=Homo sapiens OX=9606 GN=PRIM1 PE=1 SV=1	1	1	0	0
Q6NZY4	Zinc finger CCHC domain-containing protein 8 OS=Homo sapiens OX=9606 GN=ZCCHC8 PE=1 SV=2	1	1	0	0
Q5H8A4	GPI ethanolamine phosphate transferase 2 OS=Homo sapiens OX=9606 GN=PIGG PE=1 SV=1	1	1	0	0
Q86XK2	F-box only protein 11 OS=Homo sapiens OX=9606 GN=FBXO11 PE=1 SV=3	1	1	0	0
Q9UJP4	Kelch-like protein 21 OS=Homo sapiens OX=9606 GN=KLHL21 PE=1 SV=4	1	1	0	0
Q9UKE5	TRAF2 and NCK-interacting protein kinase OS=Homo sapiens OX=9606 GN=TNIK PE=1 SV=1	1	1	0	0

Q9Y5Q8	General transcription factor 3C polypeptide 5 OS=Homo sapiens OX=9606 GN=GTF3C5 PE=1 SV=2	1	1	0	0
Q8N5G2	Macoilin OS=Homo sapiens OX=9606 GN=MACO1 PE=1 SV=1	1	1	0	0
P23945	Follicle-stimulating hormone receptor OS=Homo sapiens OX=9606 GN=FSHR PE=1 SV=3	1	1	0	0
Q9BYC9	39S ribosomal protein L20, mitochondrial OS=Homo sapiens OX=9606 GN=MRPL20 PE=1 SV=1	1	1	0	0
Q7Z3V4	Ubiquitin-protein ligase E3B OS=Homo sapiens OX=9606 GN=UBE3B PE=1 SV=3	1	1	0	0
Q9BVC6	Transmembrane protein 109 OS=Homo sapiens OX=9606 GN=TMEM109 PE=1 SV=1	1	1	0	0
P19404	NADH dehydrogenase [ubiquinone] flavoprotein 2, mitochondrial OS=Homo sapiens OX=9606 GN=NDUFV2 PE=1 SV=2	1	1	0	0
A1L3X0	Elongation of very long chain fatty acids protein 7 OS=Homo sapiens OX=9606 GN=ELOVL7 PE=1 SV=1	1	1	0	0
P10644	cAMP-dependent protein kinase type I-alpha regulatory subunit OS=Homo sapiens OX=9606 GN=PRKAR1A PE=1 SV=1	1	1	0	0
Q8IXQ5	Kelch-like protein 7 OS=Homo sapiens OX=9606 GN=KLHL7 PE=1 SV=2	1	1	0	0
Q8WVM0	Dimethyladenosine transferase 1, mitochondrial OS=Homo sapiens OX=9606 GN=TFB1M PE=1 SV=1	1	1	0	0
O95429	BAG family molecular chaperone regulator 4 OS=Homo sapiens OX=9606 GN=BAG4 PE=1 SV=1	1	1	0	0
P12109	Collagen alpha-1(VI) chain OS=Homo sapiens OX=9606 GN=COL6A1 PE=1 SV=3	1	1	0	0
Q9BQ75	Protein CMSS1 OS=Homo sapiens OX=9606 GN=CMSS1 PE=1 SV=2	1	1	0	0
P31944	Caspase-14 OS=Homo sapiens OX=9606 GN=CASP14 PE=1 SV=2	1	1	0	0
Q9BU23	Lipase maturation factor 2 OS=Homo sapiens OX=9606 GN=LMF2 PE=1 SV=2	1	1	0	0
Q9BPZ3	Polyadenylate-binding protein-interacting protein 2 OS=Homo sapiens OX=9606 GN=PAIP2 PE=1 SV=1	1	1	0	0
Q15125	3-beta-hydroxysteroid-Delta(8),Delta(7)-isomerase OS=Homo sapiens OX=9606 GN=EBP PE=1 SV=3	1	1	0	0
Q8WWQ0	PH-interacting protein OS=Homo sapiens OX=9606 GN=PHIP PE=1 SV=2	1	1	0	0
Q9NV64	Transmembrane protein 39A OS=Homo sapiens OX=9606 GN=TMEM39A PE=2 SV=1	1	1	0	0
Q12891	Hyaluronidase-2 OS=Homo sapiens OX=9606 GN=HYAL2 PE=1 SV=4	1	1	0	0
O43933	Peroxisome biogenesis factor 1 OS=Homo sapiens OX=9606 GN=PEX1 PE=1 SV=1	1	1	0	0
Q7L014	Probable ATP-dependent RNA helicase DDX46 OS=Homo sapiens OX=9606 GN=DDX46 PE=1 SV=2	1	1	0	0
Q9NYU2	UDP-glucose:glycoprotein glucosyltransferase 1 OS=Homo sapiens OX=9606 GN=UGGT1 PE=1 SV=3	1	1	0	0
Q96EL3	39S ribosomal protein L53, mitochondrial OS=Homo sapiens OX=9606 GN=MRPL53 PE=1 SV=1	1	1	0	0
P51798	H(+)/Cl(-) exchange transporter 7 OS=Homo sapiens OX=9606 GN=CLCN7 PE=1 SV=2	1	1	0	0
O15228	Dihydroxyacetone phosphate acyltransferase OS=Homo sapiens OX=9606 GN=GNPAT PE=1 SV=1	1	1	0	0
Q96JN8	Neuralized-like protein 4 OS=Homo sapiens OX=9606 GN=NEURL4 PE=1 SV=2	1	1	0	0
Q9BRX2	Protein pelota homolog OS=Homo sapiens OX=9606 GN=PELO PE=1 SV=2	1	1	0	0
Q2TAY7	WD40 repeat-containing protein SMU1 OS=Homo sapiens OX=9606 GN=SMU1 PE=1 SV=2	1	1	0	0
Q9P266	Junctional protein associated with coronary artery disease OS=Homo sapiens OX=9606 GN=JCAD PE=1 SV=3	1	1	0	0
Q9NXV6	CDKN2A-interacting protein OS=Homo sapiens OX=9606 GN=CDKN2AIP PE=1 SV=3	1	1	0	0
Q495W5	Alpha-(1,3)-fucosyltransferase 11 OS=Homo sapiens OX=9606 GN=FUT11 PE=1 SV=1	1	1	0	0
Q8N8Q8	Cytochrome c oxidase assembly protein COX18, mitochondrial OS=Homo sapiens OX=9606 GN=COX18 PE=1 SV=1	1	1	0	0
Q9H5Z1	Probable ATP-dependent RNA helicase DHX35 OS=Homo sapiens OX=9606 GN=DHX35 PE=1 SV=2	1	1	0	0
O43676	NADH dehydrogenase [ubiquinone] 1 beta subcomplex subunit 3 OS=Homo sapiens OX=9606 GN=NDUFB3 PE=1 SV=3	1	1	0	0
Q99081	Transcription factor 12 OS=Homo sapiens OX=9606 GN=TCF12 PE=1 SV=1	1	1	0	0
Q01826	DNA-binding protein SATB1 OS=Homo sapiens OX=9606 GN=SATB1 PE=1 SV=1	1	1	0	0
O60888	Protein CutA OS=Homo sapiens OX=9606 GN=CUTA PE=1 SV=2	1	1	0	0
P38571	Lysosomal acid lipase/cholesteryl ester hydrolase OS=Homo sapiens OX=9606 GN=LIPA PE=1 SV=2	1	1	0	0
Q9NWW7	Uncharacterized protein C2orf42 OS=Homo sapiens OX=9606 GN=C2orf42 PE=1 SV=1	1	1	0	0
Q6NXT4	Zinc transporter 6 OS=Homo sapiens OX=9606 GN=SLC30A6 PE=1 SV=2	1	1	0	0
Q3SY17	Solute carrier family 25 member 52 OS=Homo sapiens OX=9606 GN=SLC25A52 PE=2 SV=2	1	1	0	0
Q13445	Transmembrane emp24 domain-containing protein 1 OS=Homo sapiens OX=9606 GN=TMED1 PE=1 SV=1	1	1	0	0
Q9NQT5	Exosome complex component RRP40 OS=Homo sapiens OX=9606 GN=EXOSC3 PE=1 SV=3	1	1	0	0
Q96JB5	CDK5 regulatory subunit-associated protein 3 OS=Homo sapiens OX=9606 GN=CDK5RAP3 PE=1 SV=2	1	1	0	0
P15531	Nucleoside diphosphate kinase A OS=Homo sapiens OX=9606 GN=NME1 PE=1 SV=1	1	1	0	0
Q8WVV9	Heterogeneous nuclear ribonucleoprotein L-like OS=Homo sapiens OX=9606 GN=HNRNPLL PE=1 SV=1	1	1	0	0
Q5TGZ0	MICOS complex subunit MIC10 OS=Homo sapiens OX=9606 GN=MINOS1 PE=1 SV=1	1	1	0	0
Q9Y333	U6 snRNA-associated Sm-like protein LSm2 OS=Homo sapiens OX=9606 GN=LSM2 PE=1 SV=1	1	1	0	0
Q6PCB7	Long-chain fatty acid transport protein 1 OS=Homo sapiens OX=9606 GN=SLC27A1 PE=1 SV=1	1	1	0	0
Q5SQH8	Uncharacterized protein C6orf136 OS=Homo sapiens OX=9606 GN=C6orf136 PE=2 SV=1	1	1	0	0
Q9UGH3	Solute carrier family 23 member 2 OS=Homo sapiens OX=9606 GN=SLC23A2 PE=1 SV=1	1	1	0	0
Q3MIX3	Uncharacterized aarF domain-containing protein kinase 5 OS=Homo sapiens OX=9606 GN=ADCK5 PE=1 SV=2	1	1	0	0
Q14331	Protein FRG1 OS=Homo sapiens OX=9606 GN=FRG1 PE=1 SV=1	1	1	0	0
P27816	Microtubule-associated protein 4 OS=Homo sapiens OX=9606 GN=MAP4 PE=1 SV=3	1	1	0	0

Q9BRY0	Zinc transporter ZIP3 OS=Homo sapiens OX=9606 GN=SLC39A3 PE=1 SV=2	1	1	0	0
Q13572	Inositol-tetrakisphosphate 1-kinase OS=Homo sapiens OX=9606 GN=ITPK1 PE=1 SV=2	1	1	0	0
Q9UKJ3	G patch domain-containing protein 8 OS=Homo sapiens OX=9606 GN=GPATCH8 PE=1 SV=2	1	1	0	0
Q9C0E8	Endoplasmic reticulum junction formation protein lunapark OS=Homo sapiens OX=9606 GN=LNPK PE=1 SV=2	1	1	0	0
Q9Y2R5	28S ribosomal protein S17, mitochondrial OS=Homo sapiens OX=9606 GN=MRPS17 PE=1 SV=1	1	1	0	0
Q86US8	Telomerase-binding protein EST1A OS=Homo sapiens OX=9606 GN=SMG6 PE=1 SV=2	1	1	0	0
P47974	mRNA decay activator protein ZFP36L2 OS=Homo sapiens OX=9606 GN=ZFP36L2 PE=1 SV=3	1	1	0	0
Q8IZ69	tRNA (uracil-5-)-methyltransferase homolog A OS=Homo sapiens OX=9606 GN=TRMT2A PE=1 SV=2	1	1	0	0
P25311	Zinc-alpha-2-glycoprotein OS=Homo sapiens OX=9606 GN=AZGP1 PE=1 SV=2	1	1	0	0
Q4G176	Acyl-CoA synthetase family member 3, mitochondrial OS=Homo sapiens OX=9606 GN=ACSF3 PE=1 SV=3	1	1	0	0
P61927	60S ribosomal protein L37 OS=Homo sapiens OX=9606 GN=RPL37 PE=1 SV=2	1	1	0	0
Q8NI37	Protein phosphatase PTC7 homolog OS=Homo sapiens OX=9606 GN=PPTC7 PE=2 SV=1	1	1	0	0
Q7L0Y3	tRNA methyltransferase 10 homolog C OS=Homo sapiens OX=9606 GN=TRMT10C PE=1 SV=2	1	1	0	0
Q8IXH7	Negative elongation factor C/D OS=Homo sapiens OX=9606 GN=NELFCD PE=1 SV=2	1	1	0	0
Q8NFQ6	BPI fold-containing family C protein OS=Homo sapiens OX=9606 GN=BPIFC PE=2 SV=1	1	1	0	0
Q9UNS2	COP9 signalosome complex subunit 3 OS=Homo sapiens OX=9606 GN=COPS3 PE=1 SV=3	1	1	0	0
P01023	Alpha-2-macroglobulin OS=Homo sapiens OX=9606 GN=A2M PE=1 SV=3	1	1	0	0
Q6ZNC8	Lysophospholipid acyltransferase 1 OS=Homo sapiens OX=9606 GN=MBOAT1 PE=1 SV=1	1	1	0	0
Q13951	Core-binding factor subunit beta OS=Homo sapiens OX=9606 GN=CBFB PE=1 SV=2	1	1	0	0
A6NNS2	Dehydrogenase/reductase SDR family member 7C OS=Homo sapiens OX=9606 GN=DHRS7C PE=2 SV=3	1	1	0	0
Q2QL34	Mpv17-like protein OS=Homo sapiens OX=9606 GN=MPV17L PE=1 SV=1	1	1	0	0
Q9BVK8	Transmembrane protein 147 OS=Homo sapiens OX=9606 GN=TMEM147 PE=1 SV=1	1	1	0	0
Q8NHQ9	ATP-dependent RNA helicase DDX55 OS=Homo sapiens OX=9606 GN=DDX55 PE=1 SV=3	1	1	0	0
Q5T653	39S ribosomal protein L2, mitochondrial OS=Homo sapiens OX=9606 GN=MRPL2 PE=1 SV=2	1	1	0	0
Q03518	Antigen peptide transporter 1 OS=Homo sapiens OX=9606 GN=TAP1 PE=1 SV=2	1	1	0	0
Q99720	Sigma non-opioid intracellular receptor 1 OS=Homo sapiens OX=9606 GN=SIGMAR1 PE=1 SV=1	1	1	0	0
Q9NWS1	PCNA-interacting partner OS=Homo sapiens OX=9606 GN=PARPBP PE=1 SV=3	1	1	0	0
Q9Y4Z0	U6 snRNA-associated Sm-like protein LSm4 OS=Homo sapiens OX=9606 GN=LSM4 PE=1 SV=1	1	1	0	0
Q9Y4L5	E3 ubiquitin-protein ligase RNF115 OS=Homo sapiens OX=9606 GN=RNF115 PE=1 SV=2	1	1	0	0
Q9Y4D8	Probable E3 ubiquitin-protein ligase HECTD4 OS=Homo sapiens OX=9606 GN=HECTD4 PE=1 SV=5	1	1	0	0
P49721	Proteasome subunit beta type-2 OS=Homo sapiens OX=9606 GN=PSMB2 PE=1 SV=1	1	1	0	0
Q8N5C6	S1 RNA-binding domain-containing protein 1 OS=Homo sapiens OX=9606 GN=SRBD1 PE=1 SV=2	1	1	0	0
P43007	Neutral amino acid transporter A OS=Homo sapiens OX=9606 GN=SLC1A4 PE=1 SV=1	1	1	0	0
Q86VD7	Mitochondrial coenzyme A transporter SLC25A42 OS=Homo sapiens OX=9606 GN=SLC25A42 PE=2 SV=2	1	1	0	0
Q8N0U8	Vitamin K epoxide reductase complex subunit 1-like protein 1 OS=Homo sapiens OX=9606 GN=VKORC1L1 PE=1 SV=2	1	1	0	0
O75665	Oral-facial-digital syndrome 1 protein OS=Homo sapiens OX=9606 GN=OFD1 PE=1 SV=1	1	1	0	0
Q7L592	Protein arginine methyltransferase NDUFAF7, mitochondrial OS=Homo sapiens OX=9606 GN=NDUFAF7 PE=1 SV=1	1	1	0	0
Q96DB5	Regulator of microtubule dynamics protein 1 OS=Homo sapiens OX=9606 GN=RMDN1 PE=1 SV=1	1	1	0	0
Q96J84	Kin of IRRE-like protein 1 OS=Homo sapiens OX=9606 GN=KIRREL1 PE=1 SV=2	1	1	0	0
Q8NG06	E3 ubiquitin-protein ligase TRIM58 OS=Homo sapiens OX=9606 GN=TRIM58 PE=2 SV=2	1	1	0	0
O95639	Cleavage and polyadenylation specificity factor subunit 4 OS=Homo sapiens OX=9606 GN=CPSF4 PE=1 SV=1	1	1	0	0
Q92968	Peroxisomal membrane protein PEX13 OS=Homo sapiens OX=9606 GN=PEX13 PE=1 SV=2	1	1	0	0
Q8NFQ8	Torsin-1A-interacting protein 2 OS=Homo sapiens OX=9606 GN=TOR1AIP2 PE=1 SV=1	1	1	0	0
Q9Y6G3	39S ribosomal protein L42, mitochondrial OS=Homo sapiens OX=9606 GN=MRPL42 PE=1 SV=1	1	1	0	0
Q96JQ2	Calmin OS=Homo sapiens OX=9606 GN=CLMN PE=1 SV=1	1	1	0	0
P48449	Lanosterol synthase OS=Homo sapiens OX=9606 GN=LSS PE=1 SV=1	1	1	0	0
Q9BUL9	Ribonuclease P protein subunit p25 OS=Homo sapiens OX=9606 GN=RPP25 PE=1 SV=1	1	1	0	0
Q9UHK0	Nuclear fragile X mental retardation-interacting protein 1 OS=Homo sapiens OX=9606 GN=NUFIP1 PE=1 SV=2	1	1	0	0
Q06265	Exosome complex component RRP45 OS=Homo sapiens OX=9606 GN=EXOSC9 PE=1 SV=3	1	1	0	0
P14859	POU domain, class 2, transcription factor 1 OS=Homo sapiens OX=9606 GN=POU2F1 PE=1 SV=2	1	1	0	0
O15090	Zinc finger protein 536 OS=Homo sapiens OX=9606 GN=ZNF536 PE=1 SV=3	1	1	0	0
Q15834	Coiled-coil domain-containing protein 85B OS=Homo sapiens OX=9606 GN=CCDC85B PE=1 SV=2	1	1	0	0
Q96GC9	Vacuole membrane protein 1 OS=Homo sapiens OX=9606 GN=VMP1 PE=1 SV=1	1	1	0	0
O60507	Protein-tyrosine sulfotransferase 1 OS=Homo sapiens OX=9606 GN=TPST1 PE=1 SV=1	1	1	0	0
P51149	Ras-related protein Rab-7a OS=Homo sapiens OX=9606 GN=RAB7A PE=1 SV=1	1	1	0	0
O75352	Mannose-P-dolichol utilization defect 1 protein OS=Homo sapiens OX=9606 GN=MPDU1 PE=1 SV=2	1	1	0	0

O00178	GTP-binding protein 1 OS=Homo sapiens OX=9606 GN=GTPBP1 PE=1 SV=3	1	1	0	0
Q96DW6	Mitochondrial glycine transporter OS=Homo sapiens OX=9606 GN=SLC25A38 PE=1 SV=1	1	1	0	0
P51151	Ras-related protein Rab-9A OS=Homo sapiens OX=9606 GN=RAB9A PE=1 SV=1	1	1	0	0
Q92626	Peroxidasin homolog OS=Homo sapiens OX=9606 GN=PXDN PE=1 SV=2	1	1	0	0
Q7RTS9	Dymeclin OS=Homo sapiens OX=9606 GN=DYM PE=1 SV=1	1	1	0	0
Q86YT6	E3 ubiquitin-protein ligase MIB1 OS=Homo sapiens OX=9606 GN=MIB1 PE=1 SV=1	1	1	0	0
Q9H9E3	Conserved oligomeric Golgi complex subunit 4 OS=Homo sapiens OX=9606 GN=COG4 PE=1 SV=3	1	1	0	0
Q9HCH3	Copine-5 OS=Homo sapiens OX=9606 GN=CPNE5 PE=1 SV=2	1	1	0	0
Q9UL40	Zinc finger protein 346 OS=Homo sapiens OX=9606 GN=ZNF346 PE=1 SV=1	1	1	0	0
O96005	Cleft lip and palate transmembrane protein 1 OS=Homo sapiens OX=9606 GN=CLPTM1 PE=1 SV=1	1	1	0	0
Q8IUX7	Adipocyte enhancer-binding protein 1 OS=Homo sapiens OX=9606 GN=AEBP1 PE=1 SV=1	1	1	0	0
Q8WUD6	Cholinephosphotransferase 1 OS=Homo sapiens OX=9606 GN=CHPT1 PE=1 SV=1	1	1	0	0
Q9BZI7	Regulator of nonsense transcripts 3B OS=Homo sapiens OX=9606 GN=UPF3B PE=1 SV=1	1	1	0	0
Q8N6T3	ADP-ribosylation factor GTPase-activating protein 1 OS=Homo sapiens OX=9606 GN=ARFGAP1 PE=1 SV=2	1	1	0	0
Q9Y6M4	Casein kinase I isoform gamma-3 OS=Homo sapiens OX=9606 GN=CSNK1G3 PE=1 SV=2	1	1	0	0
O60306	RNA helicase aquarius OS=Homo sapiens OX=9606 GN=AQR PE=1 SV=4	1	1	0	0
Q13618	Cullin-3 OS=Homo sapiens OX=9606 GN=CUL3 PE=1 SV=2	1	1	0	0
O14512	Suppressor of cytokine signaling 7 OS=Homo sapiens OX=9606 GN=SOCS7 PE=1 SV=2	1	1	0	0
Q8TD43	Transient receptor potential cation channel subfamily M member 4 OS=Homo sapiens OX=9606 GN=TRPM4 PE=1 SV=1	1	1	0	0
Q6NUQ1	RAD50-interacting protein 1 OS=Homo sapiens OX=9606 GN=RINT1 PE=1 SV=1	1	1	0	0
P08651	Nuclear factor 1 C-type OS=Homo sapiens OX=9606 GN=NFIC PE=1 SV=2	1	1	0	0
Q5H9L4	Transcription initiation factor TFIID subunit 7-like OS=Homo sapiens OX=9606 GN=TAF7L PE=2 SV=1	1	1	0	0
Q9UKI2	Cdc42 effector protein 3 OS=Homo sapiens OX=9606 GN=CDC42EP3 PE=1 SV=1	1	1	0	0
O75608	Acyl-protein thioesterase 1 OS=Homo sapiens OX=9606 GN=LYPLA1 PE=1 SV=1	1	1	0	0
Q15173	Serine/threonine-protein phosphatase 2A 56 kDa regulatory subunit beta isoform OS=Homo sapiens OX=9606 GN=PPP2R5B PE=1 SV=1	1	1	0	0
Q8WU76	Sec1 family domain-containing protein 2 OS=Homo sapiens OX=9606 GN=SCFD2 PE=1 SV=2	1	1	0	0
Q70JA7	Chondroitin sulfate synthase 3 OS=Homo sapiens OX=9606 GN=CHSY3 PE=2 SV=3	1	1	0	0
P20339	Ras-related protein Rab-5A OS=Homo sapiens OX=9606 GN=RAB5A PE=1 SV=2	1	1	0	0
Q9UKU6	Thyrotropin-releasing hormone-degrading ectoenzyme OS=Homo sapiens OX=9606 GN=TRHDE PE=2 SV=1	1	1	0	0
Q14679	Tubulin polyglutamylase TTLL4 OS=Homo sapiens OX=9606 GN=TTLL4 PE=1 SV=2	1	1	0	0
P51398	28S ribosomal protein S29, mitochondrial OS=Homo sapiens OX=9606 GN=DAP3 PE=1 SV=1	1	1	0	0
Q53HV7	Single-strand selective monofunctional uracil DNA glycosylase OS=Homo sapiens OX=9606 GN=SMUG1 PE=1 SV=2	1	1	0	0
Q99956	Dual specificity protein phosphatase 9 OS=Homo sapiens OX=9606 GN=DUSP9 PE=1 SV=1	1	1	0	0
Q9BRR6	ADP-dependent glucokinase OS=Homo sapiens OX=9606 GN=ADPGK PE=1 SV=1	1	1	0	0
Q9P270	SLAIN motif-containing protein 2 OS=Homo sapiens OX=9606 GN=SLAIN2 PE=1 SV=2	1	1	0	0
Q8IYS2	Uncharacterized protein KIAA2013 OS=Homo sapiens OX=9606 GN=KIAA2013 PE=1 SV=1	1	1	0	0
Q8WZ42	Titin OS=Homo sapiens OX=9606 GN=TTN PE=1 SV=4	1	1	0	0
Q96JB2	Conserved oligomeric Golgi complex subunit 3 OS=Homo sapiens OX=9606 GN=COG3 PE=1 SV=3	1	1	0	0
Q86VR2	Reticulophagy regulator 3 OS=Homo sapiens OX=9606 GN=RETREG3 PE=1 SV=1	1	1	0	0
Q14677	Clathrin interactor 1 OS=Homo sapiens OX=9606 GN=CLINT1 PE=1 SV=1	1	1	0	0
Q8WX92	Negative elongation factor B OS=Homo sapiens OX=9606 GN=NELFB PE=1 SV=1	1	1	0	0
O14929	Histone acetyltransferase type B catalytic subunit OS=Homo sapiens OX=9606 GN=HAT1 PE=1 SV=1	1	1	0	0
Q06587	E3 ubiquitin-protein ligase RING1 OS=Homo sapiens OX=9606 GN=RING1 PE=1 SV=2	1	1	0	0
Q8TEW8	Partitioning defective 3 homolog B OS=Homo sapiens OX=9606 GN=PARD3B PE=1 SV=2	1	1	0	0
Q6ZVL6	UPF0606 protein KIAA1549L OS=Homo sapiens OX=9606 GN=KIAA1549L PE=2 SV=2	1	1	0	0
P56182	Ribosomal RNA processing protein 1 homolog A OS=Homo sapiens OX=9606 GN=RRP1 PE=1 SV=1	1	1	0	0
Q9UII4	E3 ISG15--protein ligase HERC5 OS=Homo sapiens OX=9606 GN=HERC5 PE=1 SV=2	1	1	0	0
Q6P2H3	Centrosomal protein of 85 kDa OS=Homo sapiens OX=9606 GN=CEP85 PE=1 SV=1	1	1	0	0
Q8N2U9	PQ-loop repeat-containing protein 1 OS=Homo sapiens OX=9606 GN=PQLC1 PE=1 SV=1	1	1	0	0
Q9NWZ8	Gem-associated protein 8 OS=Homo sapiens OX=9606 GN=GEMIN8 PE=1 SV=1	1	1	0	0
Q6P2C8	Mediator of RNA polymerase II transcription subunit 27 OS=Homo sapiens OX=9606 GN=MED27 PE=1 SV=1	1	1	0	0
P10301	Ras-related protein R-Ras OS=Homo sapiens OX=9606 GN=RRAS PE=1 SV=1	1	1	0	0
P57678	Gem-associated protein 4 OS=Homo sapiens OX=9606 GN=GEMIN4 PE=1 SV=2	1	1	0	0
Q99612	Krueppel-like factor 6 OS=Homo sapiens OX=9606 GN=KLF6 PE=1 SV=3	1	1	0	0
P20674	Cytochrome c oxidase subunit 5A, mitochondrial OS=Homo sapiens OX=9606 GN=COX5A PE=1 SV=2	1	1	0	0
Q96GM8	Target of EGR1 protein 1 OS=Homo sapiens OX=9606 GN=TOE1 PE=1 SV=1	1	1	0	0

Q9GZM5	Protein YIPF3 OS=Homo sapiens OX=9606 GN=YIPF3 PE=1 SV=1	1	1	0	0
P12273	Prolactin-inducible protein OS=Homo sapiens OX=9606 GN=PIP PE=1 SV=1	1	1	0	0
Q9Y3E0	Vesicle transport protein GOT1B OS=Homo sapiens OX=9606 GN=GOLT1B PE=1 SV=1	1	1	0	0
Q58A45	PAN2-PAN3 deadenylation complex subunit PAN3 OS=Homo sapiens OX=9606 GN=PAN3 PE=1 SV=3	1	1	0	0
P28360	Homeobox protein MSX-1 OS=Homo sapiens OX=9606 GN=MSX1 PE=1 SV=3	1	1	0	0
P29317	Ephrin type-A receptor 2 OS=Homo sapiens OX=9606 GN=EPHA2 PE=1 SV=2	1	1	0	0
Q53HI1	Protein unc-50 homolog OS=Homo sapiens OX=9606 GN=UNC50 PE=1 SV=2	1	1	0	0
P16219	Short-chain specific acyl-CoA dehydrogenase, mitochondrial OS=Homo sapiens OX=9606 GN=ACADS PE=1 SV=1	1	1	0	0
Q8N6M3	Fat storage-inducing transmembrane protein 2 OS=Homo sapiens OX=9606 GN=FITM2 PE=2 SV=1	1	1	0	0
P32322	Pyrroline-5-carboxylate reductase 1, mitochondrial OS=Homo sapiens OX=9606 GN=PYCR1 PE=1 SV=2	1	1	0	0
P18405	3-oxo-5-alpha-steroid 4-dehydrogenase 1 OS=Homo sapiens OX=9606 GN=SRD5A1 PE=1 SV=1	1	1	0	0
Q6Y1H2	Very-long-chain (3R)-3-hydroxyacyl-CoA dehydratase 2 OS=Homo sapiens OX=9606 GN=HACD2 PE=1 SV=1	1	1	0	0
O94887	FERM, ARHGEF and pleckstrin domain-containing protein 2 OS=Homo sapiens OX=9606 GN=FARP2 PE=1 SV=3	1	1	0	0
P13489	Ribonuclease inhibitor OS=Homo sapiens OX=9606 GN=RNH1 PE=1 SV=2	1	1	0	0
Q8WVD3	E3 ubiquitin-protein ligase RNF138 OS=Homo sapiens OX=9606 GN=RNF138 PE=1 SV=1	1	1	0	0
A6NJ78	Probable methyltransferase-like protein 15 OS=Homo sapiens OX=9606 GN=METTL15 PE=1 SV=1	1	1	0	0
Q8IWR0	Zinc finger CCCH domain-containing protein 7A OS=Homo sapiens OX=9606 GN=ZC3H7A PE=1 SV=1	1	1	0	0
O60262	Guanine nucleotide-binding protein G(I)/G(S)/G(O) subunit gamma-7 OS=Homo sapiens OX=9606 GN=GNG7 PE=1 SV=1	1	1	0	0
Q9UFG5	UPF0449 protein C19orf25 OS=Homo sapiens OX=9606 GN=C19orf25 PE=1 SV=2	1	1	0	0
Q14493	Histone RNA hairpin-binding protein OS=Homo sapiens OX=9606 GN=SLBP PE=1 SV=1	1	1	0	0
P52701	DNA mismatch repair protein Msh6 OS=Homo sapiens OX=9606 GN=MSH6 PE=1 SV=2	1	1	0	0
P08572	Collagen alpha-2(IV) chain OS=Homo sapiens OX=9606 GN=COL4A2 PE=1 SV=4	1	1	0	0
Q96SY0	Integrator complex subunit 14 OS=Homo sapiens OX=9606 GN=INTS14 PE=1 SV=2	1	1	0	0
O14529	Homeobox protein cut-like 2 OS=Homo sapiens OX=9606 GN=CUX2 PE=1 SV=4	1	1	0	0
Q9NPQ8	Synembryn-A OS=Homo sapiens OX=9606 GN=RIC8A PE=1 SV=3	1	1	0	0
O15427	Monocarboxylate transporter 4 OS=Homo sapiens OX=9606 GN=SLC16A3 PE=1 SV=1	1	1	0	0
Q5TAT6	Collagen alpha-1(XIII) chain OS=Homo sapiens OX=9606 GN=COL13A1 PE=1 SV=1	1	1	0	0
O00487	26S proteasome non-ATPase regulatory subunit 14 OS=Homo sapiens OX=9606 GN=PSMD14 PE=1 SV=1	1	1	0	0
Q5VUJ6	Leucine-rich repeat and calponin homology domain-containing protein 2 OS=Homo sapiens OX=9606 GN=LRCH2 PE=2 SV=2	1	1	0	0
Q02040	A-kinase anchor protein 17A OS=Homo sapiens OX=9606 GN=AKAP17A PE=1 SV=2	1	1	0	0
O43731	ER lumen protein-retaining receptor 3 OS=Homo sapiens OX=9606 GN=KDELR3 PE=2 SV=1	1	1	0	0
Q6DJT9	Zinc finger protein PLAG1 OS=Homo sapiens OX=9606 GN=PLAG1 PE=1 SV=1	1	1	0	0
P32242	Homeobox protein OTX1 OS=Homo sapiens OX=9606 GN=OTX1 PE=1 SV=1	1	1	0	0
Q3SXY8	ADP-ribosylation factor-like protein 13B OS=Homo sapiens OX=9606 GN=ARL13B PE=1 SV=1	1	1	0	0
Q12893	Transmembrane protein 115 OS=Homo sapiens OX=9606 GN=TMEM115 PE=1 SV=1	1	1	0	0
O15527	N-glycosylase/DNA lyase OS=Homo sapiens OX=9606 GN=OGG1 PE=1 SV=2	1	1	0	0
P29508	Serpin B3 OS=Homo sapiens OX=9606 GN=SERPINB3 PE=1 SV=2	1	1	0	0
Q02809	Procollagen-lysine,2-oxoglutarate 5-dioxygenase 1 OS=Homo sapiens OX=9606 GN=PLOD1 PE=1 SV=2	1	1	0	0
Q96IU4	Protein ABHD14B OS=Homo sapiens OX=9606 GN=ABHD14B PE=1 SV=1	1	1	0	0
Q08629	Testican-1 OS=Homo sapiens OX=9606 GN=SPOCK1 PE=1 SV=1	1	1	0	0
Q9H4W6	Transcription factor COE3 OS=Homo sapiens OX=9606 GN=EBF3 PE=1 SV=2	1	1	0	0
Q99698	Lysosomal-trafficking regulator OS=Homo sapiens OX=9606 GN=LYST PE=1 SV=3	1	1	0	0
Q9H974	Queuine tRNA-ribosyltransferase accessory subunit 2 OS=Homo sapiens OX=9606 GN=QTRT2 PE=1 SV=1	1	1	0	0
Q8NHM5	Lysine-specific demethylase 2B OS=Homo sapiens OX=9606 GN=KDM2B PE=1 SV=1	1	1	0	0
P22033	Methylmalonyl-CoA mutase, mitochondrial OS=Homo sapiens OX=9606 GN=MUT PE=1 SV=4	1	1	0	0
O15234	Protein CASC3 OS=Homo sapiens OX=9606 GN=CASC3 PE=1 SV=2	1	1	0	0
P41212	Transcription factor ETV6 OS=Homo sapiens OX=9606 GN=ETV6 PE=1 SV=1	1	1	0	0
Q4KMZ1	IQ domain-containing protein C OS=Homo sapiens OX=9606 GN=IQCC PE=1 SV=2	1	1	0	0
Q9P0Z9	Peroxisomal sarcosine oxidase OS=Homo sapiens OX=9606 GN=PIPOX PE=1 SV=2	1	1	0	0
Q9BX95	Sphingosine-1-phosphate phosphatase 1 OS=Homo sapiens OX=9606 GN=SGPP1 PE=1 SV=2	1	1	0	0
Q6P2E9	Enhancer of mRNA-decapping protein 4 OS=Homo sapiens OX=9606 GN=EDC4 PE=1 SV=1	1	1	0	0
Q9NR50	Translation initiation factor eIF-2B subunit gamma OS=Homo sapiens OX=9606 GN=EIF2B3 PE=1 SV=1	1	1	0	0
Q5H9R7	Serine/threonine-protein phosphatase 6 regulatory subunit 3 OS=Homo sapiens OX=9606 GN=PPP6R3 PE=1 SV=2	1	1	0	0
Q2TB10	Zinc finger protein 800 OS=Homo sapiens OX=9606 GN=ZNF800 PE=1 SV=1	1	1	0	0
Q86SF2	N-acetylgalactosaminyltransferase 7 OS=Homo sapiens OX=9606 GN=GALNT7 PE=1 SV=1	1	1	0	0
Q8N1F8	Serine/threonine-protein kinase 11-interacting protein OS=Homo sapiens OX=9606 GN=STK11IP PE=1 SV=4	1	1	0	0

Q7L0J3	Synaptic vesicle glycoprotein 2A OS=Homo sapiens OX=9606 GN=SV2A PE=1 SV=1	1	1	0	0
Q92685	Dol-P-Man:Man(5)GlcNAc(2)-PP-Dol alpha-1,3-mannosyltransferase OS=Homo sapiens OX=9606 GN=ALG3 PE=1 SV=1	1	1	0	0
O95466	Formin-like protein 1 OS=Homo sapiens OX=9606 GN=FMNL1 PE=1 SV=3	1	1	0	0
P82675	28S ribosomal protein S5, mitochondrial OS=Homo sapiens OX=9606 GN=MRPS5 PE=1 SV=2	1	1	0	0
Q9BZK7	F-box-like/WD repeat-containing protein TBL1XR1 OS=Homo sapiens OX=9606 GN=TBL1XR1 PE=1 SV=1	1	1	0	0
Q9Y3Z3	Deoxynucleoside triphosphate triphosphohydrolase SAMHD1 OS=Homo sapiens OX=9606 GN=SAMHD1 PE=1 SV=2	1	1	0	0
Q9UPV9	Trafficking kinesin-binding protein 1 OS=Homo sapiens OX=9606 GN=TRAK1 PE=1 SV=1	1	1	0	0
Q9H1I8	Activating signal cointegrator 1 complex subunit 2 OS=Homo sapiens OX=9606 GN=ASCC2 PE=1 SV=3	1	1	0	0
P21953	2-oxoisovalerate dehydrogenase subunit beta, mitochondrial OS=Homo sapiens OX=9606 GN=BCKDHB PE=1 SV=2	1	1	0	0
Q7Z4G4	tRNA (guanine(10)-N2)-methyltransferase homolog OS=Homo sapiens OX=9606 GN=TRMT11 PE=1 SV=1	1	1	0	0
Q2TB90	Putative hexokinase HKDC1 OS=Homo sapiens OX=9606 GN=HKDC1 PE=1 SV=3	1	1	0	0
Q6PIU2	Neutral cholesterol ester hydrolase 1 OS=Homo sapiens OX=9606 GN=NCEH1 PE=1 SV=3	1	1	0	0
Q92990	Glomulin OS=Homo sapiens OX=9606 GN=GLMN PE=1 SV=2	1	1	0	0
Q9UHF7	Zinc finger transcription factor Trps1 OS=Homo sapiens OX=9606 GN=TRPS1 PE=1 SV=2	1	1	0	0
Q05BV3	Echinoderm microtubule-associated protein-like 5 OS=Homo sapiens OX=9606 GN=EML5 PE=2 SV=3	1	1	0	0
P19320	Vascular cell adhesion protein 1 OS=Homo sapiens OX=9606 GN=VCAM1 PE=1 SV=1	1	1	0	0
P36404	ADP-ribosylation factor-like protein 2 OS=Homo sapiens OX=9606 GN=ARL2 PE=1 SV=4	1	1	0	0
Q629K1	Triple QxxK/R motif-containing protein OS=Homo sapiens OX=9606 GN=TRIQK PE=3 SV=1	1	1	0	0
Q9UIV1	CCR4-NOT transcription complex subunit 7 OS=Homo sapiens OX=9606 GN=CNOT7 PE=1 SV=3	1	1	0	0
Q96AA3	Protein RFT1 homolog OS=Homo sapiens OX=9606 GN=RFT1 PE=1 SV=1	1	1	0	0
P11277	Spectrin beta chain, erythrocytic OS=Homo sapiens OX=9606 GN=SPTB PE=1 SV=5	1	1	0	0
O75817	Ribonuclease P protein subunit p20 OS=Homo sapiens OX=9606 GN=POP7 PE=1 SV=2	1	1	0	0
Q9GZR5	Elongation of very long chain fatty acids protein 4 OS=Homo sapiens OX=9606 GN=ELOVL4 PE=1 SV=1	1	1	0	0
O14777	Kinetochore protein NDC80 homolog OS=Homo sapiens OX=9606 GN=NDC80 PE=1 SV=1	1	1	0	0
O14734	Acyl-coenzyme A thioesterase 8 OS=Homo sapiens OX=9606 GN=ACOT8 PE=1 SV=1	1	1	0	0
P08559	Pyruvate dehydrogenase E1 component subunit alpha, somatic form, mitochondrial OS=Homo sapiens OX=9606 GN=PDHA1 PE=1 SV=3	1	1	0	0
Q96AK3	DNA dC->dU-editing enzyme APOBEC-3D OS=Homo sapiens OX=9606 GN=APOBEC3D PE=1 SV=1	1	1	0	0
O95067	G2/mitotic-specific cyclin-B2 OS=Homo sapiens OX=9606 GN=CCNB2 PE=1 SV=1	1	1	0	0
Q6IAA8	Ragulator complex protein LAMTOR1 OS=Homo sapiens OX=9606 GN=LAMTOR1 PE=1 SV=2	1	1	0	0
P18615	Negative elongation factor E OS=Homo sapiens OX=9606 GN=NELFE PE=1 SV=3	1	1	0	0
Q9HCL2	Glycerol-3-phosphate acyltransferase 1, mitochondrial OS=Homo sapiens OX=9606 GN=GPAM PE=1 SV=3	1	1	0	0
O15503	Insulin-induced gene 1 protein OS=Homo sapiens OX=9606 GN=INSIG1 PE=1 SV=3	1	1	0	0
Q969E2	Secretory carrier-associated membrane protein 4 OS=Homo sapiens OX=9606 GN=SCAMP4 PE=1 SV=1	1	1	0	0
Q9Y2Q9	28S ribosomal protein S28, mitochondrial OS=Homo sapiens OX=9606 GN=MRPS28 PE=1 SV=1	1	1	0	0
Q5T4B2	Inactive glycosyltransferase 25 family member 3 OS=Homo sapiens OX=9606 GN=CERCAM PE=1 SV=1	1	1	0	0
O00264	Membrane-associated progesterone receptor component 1 OS=Homo sapiens OX=9606 GN=PGRMC1 PE=1 SV=3	1	1	0	0
Q99735	Microsomal glutathione S-transferase 2 OS=Homo sapiens OX=9606 GN=MGST2 PE=1 SV=1	1	1	0	0
Q15573	TATA box-binding protein-associated factor RNA polymerase I subunit A OS=Homo sapiens OX=9606 GN=TAF1A PE=1 SV=1	1	1	0	0
P07858	Cathepsin B OS=Homo sapiens OX=9606 GN=CTSB PE=1 SV=3	1	1	0	0
O15120	1-acyl-sn-glycerol-3-phosphate acyltransferase beta OS=Homo sapiens OX=9606 GN=AGPAT2 PE=1 SV=1	1	1	0	0
Q9Y289	Sodium-dependent multivitamin transporter OS=Homo sapiens OX=9606 GN=SLC5A6 PE=2 SV=2	1	1	0	0
Q9P244	Leucine-rich repeat and fibronectin type III domain-containing protein 1 OS=Homo sapiens OX=9606 GN=LRFN1 PE=1 SV=2	1	1	0	0
Q86UT6	NLR family member X1 OS=Homo sapiens OX=9606 GN=NLRX1 PE=1 SV=1	1	1	0	0
P33908	Mannosyl-oligosaccharide 1,2-alpha-mannosidase IA OS=Homo sapiens OX=9606 GN=MAN1A1 PE=1 SV=3	1	1	0	0
Q14764	Major vault protein OS=Homo sapiens OX=9606 GN=MVP PE=1 SV=4	1	1	0	0
P15291	Beta-1,4-galactosyltransferase 1 OS=Homo sapiens OX=9606 GN=B4GALT1 PE=1 SV=5	1	1	0	0
Q96I99	Succinate--CoA ligase [GDP-forming] subunit beta, mitochondrial OS=Homo sapiens OX=9606 GN=SUCLG2 PE=1 SV=2	1	1	0	0
Q16678	Cytochrome P450 1B1 OS=Homo sapiens OX=9606 GN=CYP1B1 PE=1 SV=2	1	1	0	0
Q8NBJ7	Inactive C-alpha-formylglycine-generating enzyme 2 OS=Homo sapiens OX=9606 GN=SUMF2 PE=1 SV=2	1	1	0	0
P06858	Lipoprotein lipase OS=Homo sapiens OX=9606 GN=LPL PE=1 SV=1	1	1	0	0
Q16772	Glutathione S-transferase A3 OS=Homo sapiens OX=9606 GN=GSTA3 PE=1 SV=3	1	1	0	0
Q9NP92	39S ribosomal protein S30, mitochondrial OS=Homo sapiens OX=9606 GN=MRPS30 PE=1 SV=2	1	1	0	0
P00338	L-lactate dehydrogenase A chain OS=Homo sapiens OX=9606 GN=LDHA PE=1 SV=2	1	1	0	0
P08123	Collagen alpha-2(I) chain OS=Homo sapiens OX=9606 GN=COL1A2 PE=1 SV=7	1	1	0	0
P13861	cAMP-dependent protein kinase type II-alpha regulatory subunit OS=Homo sapiens OX=9606 GN=PRKAR2A PE=1 SV=2	1	1	0	0
Q9UHR5	SAP30-binding protein OS=Homo sapiens OX=9606 GN=SAP30BP PE=1 SV=1	1	1	0	0

Q8IXU6	Solute carrier family 35 member F2 OS=Homo sapiens OX=9606 GN=SLC35F2 PE=1 SV=1	1	1	0	0
Q03154	Aminoacylase-1 OS=Homo sapiens OX=9606 GN=ACY1 PE=1 SV=1	1	1	0	0
Q9Y5R4	HemK methyltransferase family member 1 OS=Homo sapiens OX=9606 GN=HEMK1 PE=1 SV=1	1	1	0	0
Q9HCU5	Prolactin regulatory element-binding protein OS=Homo sapiens OX=9606 GN=PREB PE=1 SV=2	1	1	0	0
Q99828	Calcium and integrin-binding protein 1 OS=Homo sapiens OX=9606 GN=CIB1 PE=1 SV=4	1	1	0	0
O95363	Phenylalanine--tRNA ligase, mitochondrial OS=Homo sapiens OX=9606 GN=FARS2 PE=1 SV=1	1	1	0	0
P16402	Histone H1.3 OS=Homo sapiens OX=9606 GN=HIST1H1D PE=1 SV=2	1	1	0	0
O75529	TAF5-like RNA polymerase II p300/CBP-associated factor-associated factor 65 kDa subunit 5L OS=Homo sapiens OX=9606 GN=TAF5L PE=1 SV=1	1	1	0	0
Q9NYK5	39S ribosomal protein L39, mitochondrial OS=Homo sapiens OX=9606 GN=MRPL39 PE=1 SV=3	1	1	0	0
P01040	Cystatin-A OS=Homo sapiens OX=9606 GN=CSTA PE=1 SV=1	1	1	0	0
Q9H4I3	TraB domain-containing protein OS=Homo sapiens OX=9606 GN=TRABD PE=1 SV=1	1	1	0	0
Q96FL9	Polypeptide N-acetylgalactosaminyltransferase 14 OS=Homo sapiens OX=9606 GN=GALNT14 PE=1 SV=1	1	1	0	0
P51649	Succinate-semialdehyde dehydrogenase, mitochondrial OS=Homo sapiens OX=9606 GN=ALDH5A1 PE=1 SV=2	1	1	0	0
Q8N4H5	Mitochondrial import receptor subunit TOM5 homolog OS=Homo sapiens OX=9606 GN=TOMM5 PE=1 SV=1	1	1	0	0
O95433	Activator of 90 kDa heat shock protein ATPase homolog 1 OS=Homo sapiens OX=9606 GN=AHSA1 PE=1 SV=1	1	1	0	0
Q13395	Probable methyltransferase TARBP1 OS=Homo sapiens OX=9606 GN=TARBP1 PE=1 SV=1	1	1	0	0
Q9NV70	Exocyst complex component 1 OS=Homo sapiens OX=9606 GN=EXOC1 PE=1 SV=4	1	1	0	0
Q9Y651	Transcription factor SOX-21 OS=Homo sapiens OX=9606 GN=SOX21 PE=2 SV=1	1	1	0	0
Q9UKR5	Probable ergosterol biosynthetic protein 28 OS=Homo sapiens OX=9606 GN=ERG28 PE=1 SV=1	1	1	0	0
A6NDU8	UPF0600 protein C5orf51 OS=Homo sapiens OX=9606 GN=C5orf51 PE=1 SV=1	1	1	0	0
A2IDD5	Coiled-coil domain-containing protein 78 OS=Homo sapiens OX=9606 GN=CCDC78 PE=2 SV=1	1	1	0	0
P32189	Glycerol kinase OS=Homo sapiens OX=9606 GN=GK PE=1 SV=3	1	1	0	0
