# Supplementary material for: 1-Deoxynojirimycin promotes cardiac function and rescues mitochondrial cristae in mitochondrial hypertrophic cardiomyopathy
Source: J Clin Invest. 2023 Jul 17;133(14):e164660. doi: 10.1172/JCI164660 (PMC10348775; doi:10.1172/JCI164660)
Supplement: Supplemental data [file jci-133-164660-s048.pdf]

Supplementary Materials for

1-Deoxynojirimycin promotes cardiac function and rescues mitochondrial cristae in mitochondrial hypertrophic cardiomyopathy

Qianqian Zhuang[1a], Fengfeng Guo[2,3a], Lei Fu[1a], Yufei Dong[1], Shaofang Xie[4], Xue Ding[1], Shuangyi Hu[1], Xuanhao D. Zhou [1], Yangwei Jiang[1], Hui Zhou[1], Yue Qiu[1], Zhaoying Lei[1], Mengyao Li[1], Huajian Cai[1], Mingjie Fan[1,2], Lingjie Sang[1], Yong Fu[5], Dong Zhang[1], Aifu Lin[1], Xu Li[4], Tilo Kunath[6], Ruhong Zhou[1*], Ping Liang[2,3*], Zhong Liu[2*], Qingfeng Yan[1,2,7,b*]

[a]These authors contributed equally to this work.

[b]Lead contact

*Corresponding author:

E-mail: rhzhou@zju.edu.cn, pingliang@zju.edu.cn, liuzhong_68@hotmail.com, qfyan@zju.edu.cn

This file includes:

Supplemental figure legends and tables

Supplemental Figure 1. Characterization of HCM patient-specific cybrids, iPSCs and iPSC-CMs.

Supplemental Figure 2. Electrophysiological Properties of HCM iPSC-CMs treated with DNJ treatment.

Supplemental Figure 3. Electrophysiological Properties of HCM iPSC-CMs treated with astragalus polyphenols and verbenalin.

Supplemental Figure 4. The parameters of calcium handling in HCM iPSC-CMs treated with DNJ.

Supplemental Figure 5. DNJ reverses pathological phenotype in HCM cybrids and iPSC-CMs.

Supplemental Figure 6. DNJ has a mild effect on mitochondrial function in control cybrids and iPSC-CMs.

Supplemental Figure 7. Electrophysiological properties of control iPSC-CMs treated with DNJ.

Supplemental Figure 8. The parameters of calcium handling in control iPSC-CMs treated with DNJ.

Supplemental Figure 9. Mass spectrum-proteomic analysis and candidate verification.

Supplemental Figure 10. DNJ rescues the mitochondrial dysfunction in HCM iPSC-CMs.

Supplemental Figure 11. The effect of DNJ on mitochondrial function in HCM cybrids with OPA1 or GAA knockdown.

Supplemental Figure 12. DNJ restores mitochondrial function in GAA-knockdown HCM iPSC-CMs.

Supplemental Figure 13. The parameters of calcium handling in HCM iPSC-CMs treated with DNJ with OPA1 or GAA knockdown.

Supplemental Figure 14. Relative mRNA levels of hypertrophy marker genes.

Supplemental Figure 15. The effect of DNJ in mice.

Supplemental Figure 16. The representative H&E staining of tissue slices from different organs of mice treated with DNJ.

Supplemental Table 1. Mitochondrial chemical molecules.

Supplemental Table 2. Effect of DNJ on action potentials recorded from HCM iPSC-CMs.

Supplemental Table 3. Effect of Astra and Verb on action potentials recorded from HCM iPSC-CMs.

Supplemental Table 4. Effect of DNJ on action potentials recorded from control iPSC-CMs.

Supplemental Table 5. Protein identification results for DNJ pulldown assay.

Supplemental Table 6. Baseline echo parameters of sham, AngII and DNJ groups.

Supplemental Table 7. Echo parameters of sham, AngII and DNJ groups after 4-week treatment.

Supplemental Table 8. Baseline echo parameters of sham and DNJ groups.

Supplemental Table 9. Echo parameters of sham and DNJ groups after 4-week treatment.

Supplemental Figures

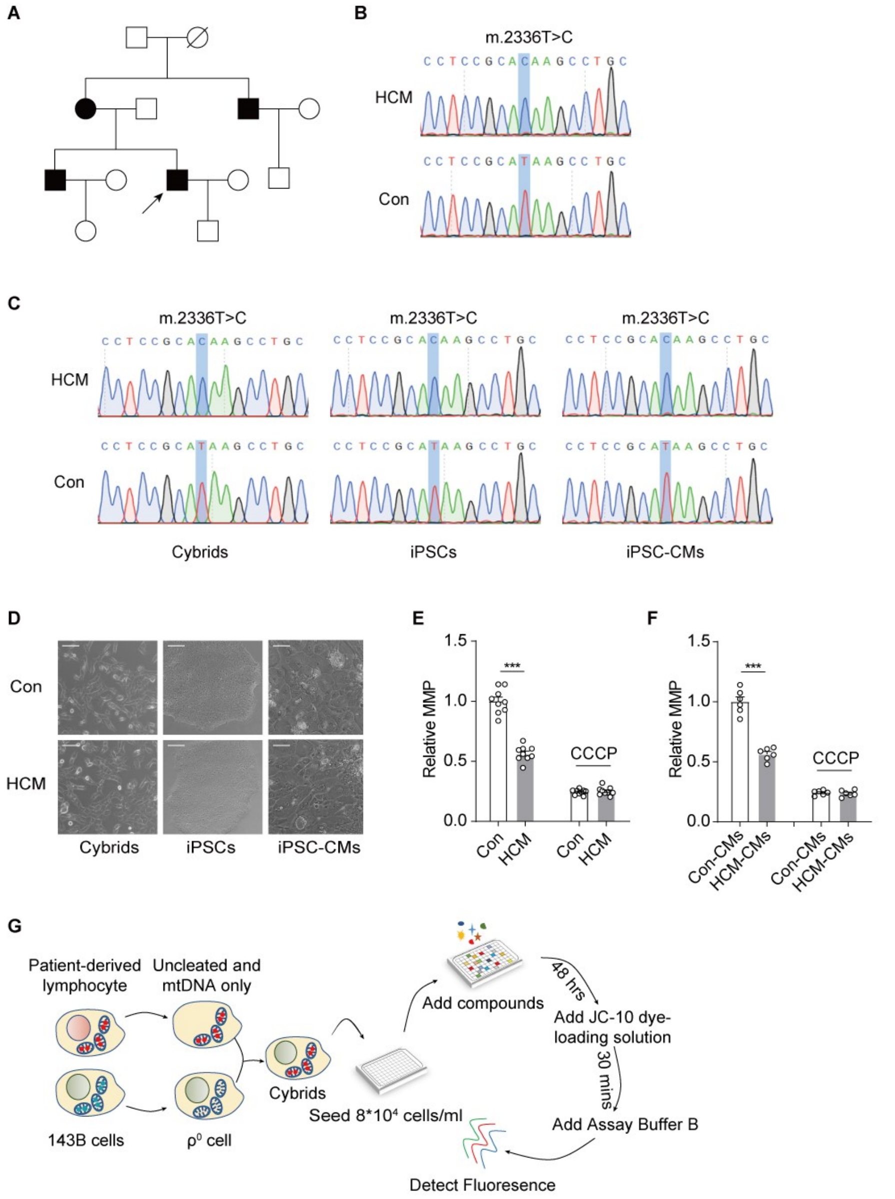

Supplemental Figure 1. Characterization of HCM patient-specific cybrids, iPSCs and iPSC-CMs. (A) The Chinese pedigree with hypertrophic cardiomyopathy (1). Affected individuals are indicated with filled symbols. The arrowhead indicates the proband. **(B)** Identification of the m.2336T>C mutation in 16S rRNA gene from the proband (1). **(C)** Confirming the existence of m.2336T>C mutation in HCM cell lines but not in controls. **(D)** Typical morphology of cybrids, iPSCs and iPSC-CMs. Scale bar, 13 µm. **(E and F)** The relative ratios of JC-10 fluorescence intensity at FL590/FL525 were calculated in the absence or presence of CCCP to reflect the mitochondrial membrane potential in HCM cybrids **(E)** or iPSC-CMs **(F)**, and with a normal group as control (2, 3) n = 3 biologically independent experiments in three cybrids lines and two iPSC-CMs cell lines. Values represent the mean ± SEM. Student's unpaired, two-tailed *t* test. ***P < 0.001. **(G)** Schematic of high-throughput chemical screen for HCM cybrids based on MMP.

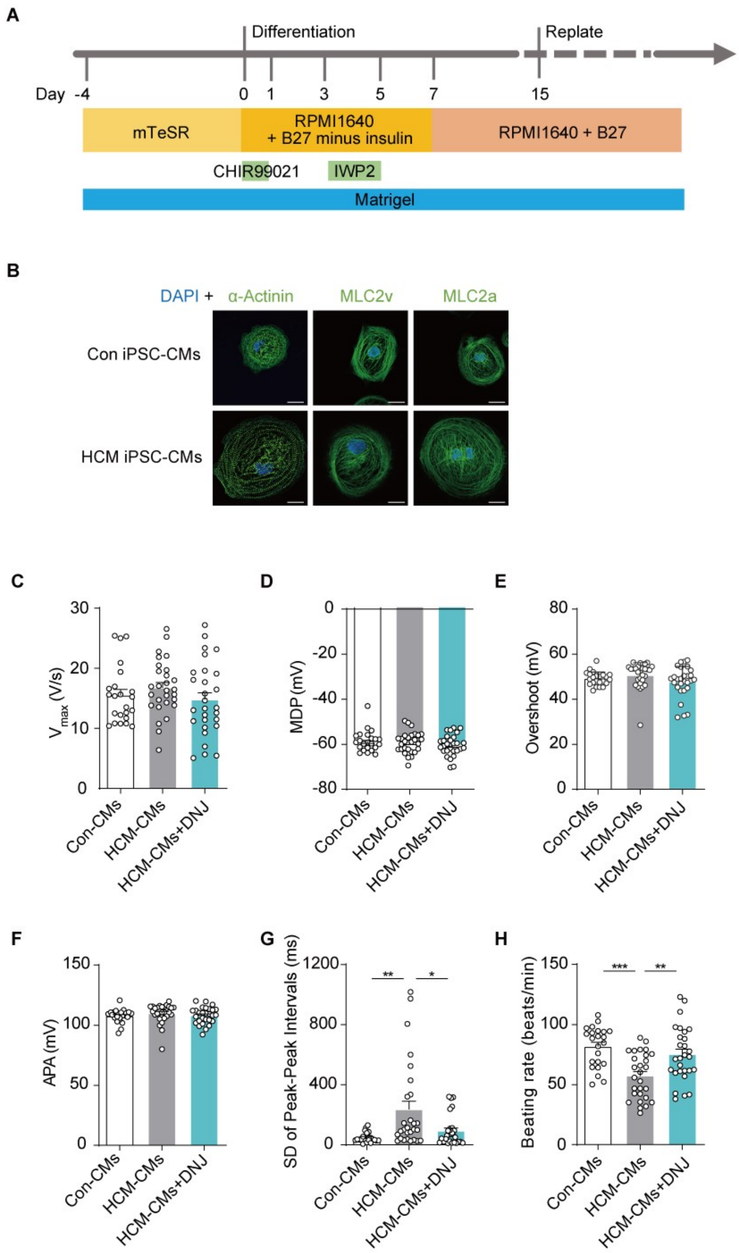

Supplemental Figure 2. Electrophysiological properties of HCM iPSC-CMs with DNJ treatment. (A) Schematic of the cardiomyocyte differentiation process. **(B)** Immunofluorescent staining of control and HCM iPSC-CMs using cardiac specific marker α-actinin, MLC2v and MLC2a. Scale bar, 40 μm. **(C-H)** Quantification of the V_{max}, MDP, overshoot, AP amplitude, SD of Peak-Peak Intervals and beating rate among three groups (Con: n = 23 in 2 lines; HCM: n = 28 in 2 lines; HCM+DNJ: n = 28 in 2 lines). Values represent the mean ± SEM. One-way ANOVA followed by Tukey's test. *$P < 0.05$, **$P < 0.01$, ***$P < 0.001$. For each group, data were collected from two different iPSC lines and at least three batches of differentiation.

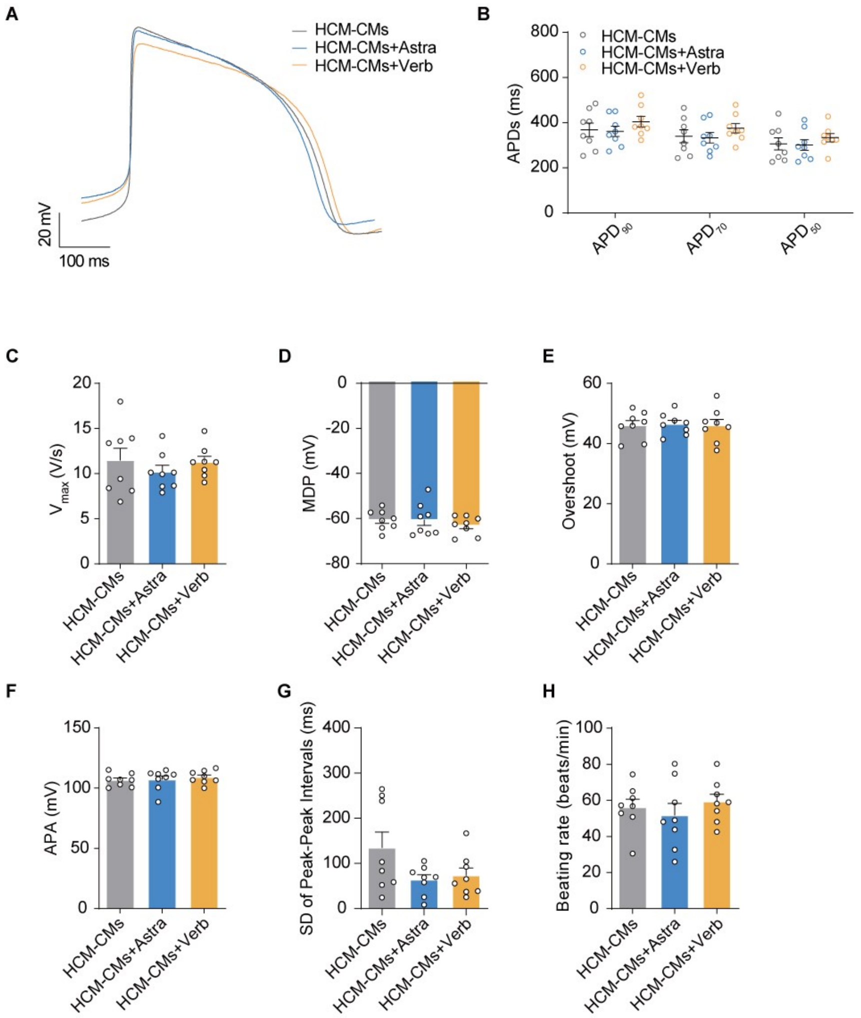

Supplemental Figure 3. Electrophysiological properties of HCM iPSC-CMs treated with astragalus polyphenols and verbenalin. (A) Representative action potential tracings of ventricular-like myocytes derived from HCM iPSC-CMs, HCM iPSC-CMs + Astra and HCM iPSC-CMs + Verb, respectively. **(B)** Quantification of action potential duration (APD) at 50% (APD_{50}), 70% (APD_{70}), and 90% (APD_{90}) repolarization in HCM iPSC-CMs treated with astragalus polyphenols or verbenalin (HCM: n = 8, HCM + Astra: n = 8, HCM + Verb: n = 8). Values represent the mean ± SEM. **(C-H)** Quantification of V_{max}, MDP, overshoot, AP amplitude, SD of Peak-Peak Intervals and beating rate among HCM iPSC-CMs, HCM + Astra and HCM + Verb (HCM: n = 8; HCM + Astra: n = 8; HCM + Verb: n = 8). Values represent the mean ± SEM.

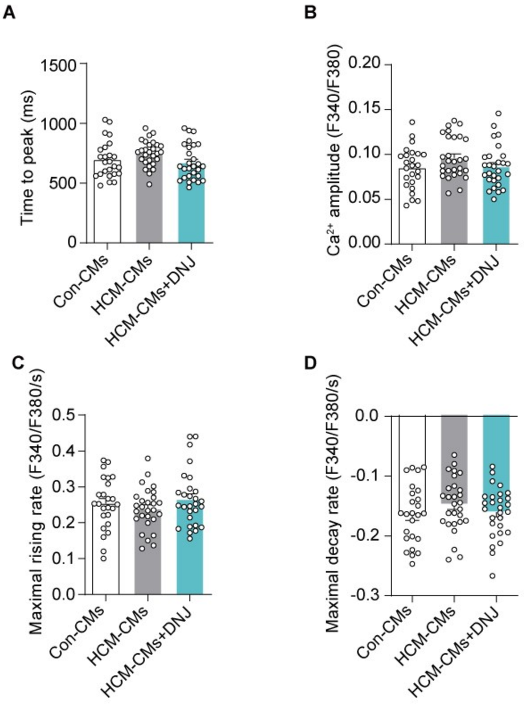

Supplemental Figure 4. The parameters of calcium handling in HCM iPSC-CMs treated with DNJ. (A-D) Quantification of time to peak, Ca^{2+} amplitude, maximal rising rate and maximal decay rate among Con iPSC-CMs, HCM iPSC-CMs and HCM iPSC-CMs + DNJ (Con: n = 25 in 2 lines; HCM: n = 28 in 2 lines; HCM + DNJ: n = 28 in 2 lines). Values represent the mean ± SEM. For each group, data were collected from two different iPSC lines and at least three batches of differentiation.

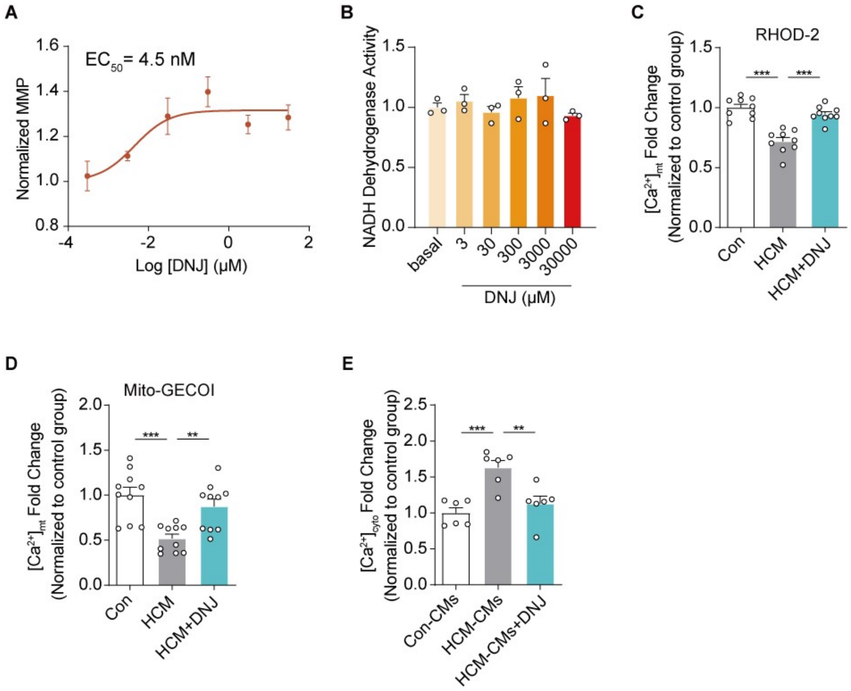

Supplemental Figure 5. DNJ reverses pathological phenotype in HCM cybrids and HCM iPSC-CMs. (A) Representative DNJ concentration-response curves are shown with MMP as an indicator in HCM cybrids. n = 3 biologically independent experiments. Values represent the mean ± SEM. Data are representative of 3 independent experiments. **(B)** Evaluation of the relationship with DNJ concentrations and cell activity in HCM cybrids. n = 3 biologically independent experiments. **(C)** Mitochondrial calcium in cybrids was detected using RHOD-2. n = 3 biologically independent experiments in three cell lines. Values represent the mean ± SEM. One-way ANOVA followed by Tukey's test. ***$P < 0.001$. **(D)** Mitochondrial calcium in cybrids were detected by mito-GECO1. n = 10 biologically independent pictures. Values represent the mean ± SEM. One-way ANOVA followed by Tukey's test. **$P < 0.01$, ***$P < 0.001$. **(E)** Analysis of intracellular calcium by the Fluo-4 indicators. n = 3 biologically independent experiments in two lines. Values represent the mean ± SEM. One-way ANOVA followed by Tukey's test. **$P < 0.01$, ***$P < 0.001$.

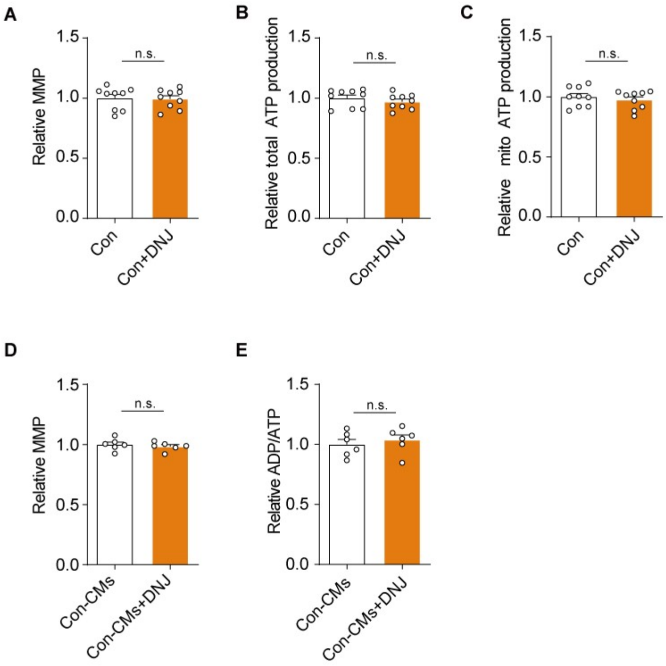

Supplemental Figure 6. DNJ has a mild effect on mitochondrial function in control cybrids and iPSC-CMs. (A-C) Mitochondrial membrane potential, total ATP and mitochondrial ATP production was measured in Con cybrids and Con cybrids + DNJ. n = 3 biologically independent experiments in three lines. Values represent the mean ± SEM. Student's unpaired, two-tailed *t* test. **(D-E)** Mitochondrial membrane potential and ADP/ATP ratio were examined in Con iPSC-CMs and Con iPSC-CMs + DNJ. n = 3 biologically independent experiments in two lines. Values represent the mean ± SEM. Student's unpaired, two-tailed *t* test.

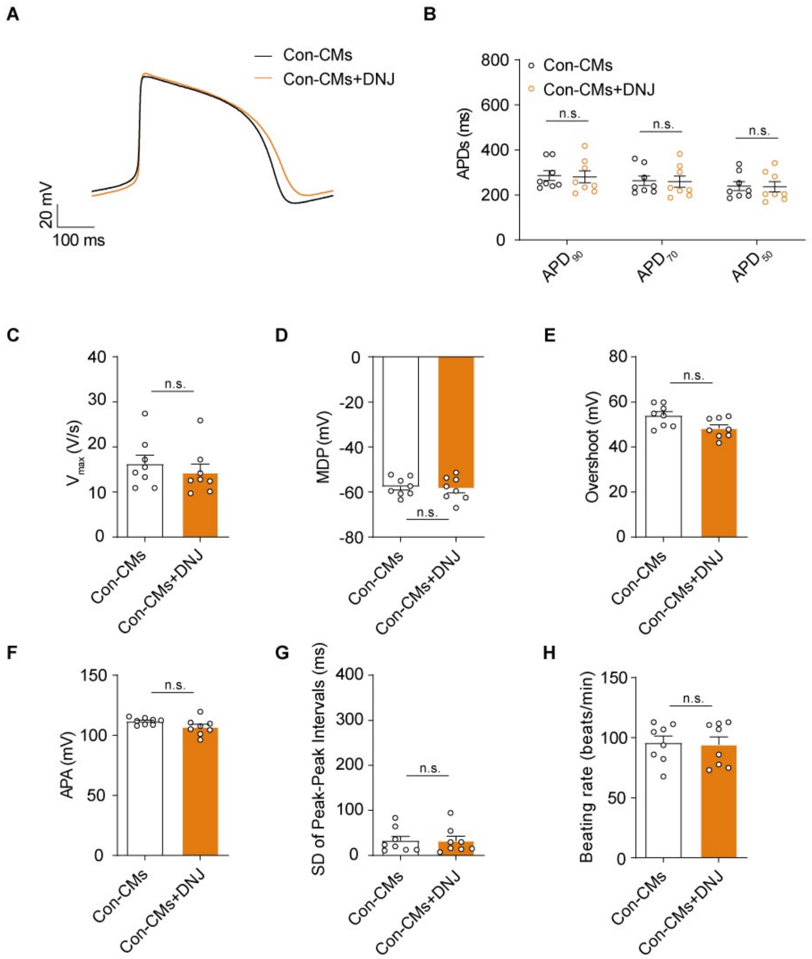

Supplemental Figure 7. Electrophysiological properties of control iPSC-CMs treated with DNJ. (A) Representative action potential tracings of ventricular-like myocytes derived from Con iPSC-CMs, Con iPSC-CMs + DNJ, respectively. **(B)** Quantification of APD_{50}, APD_{70}, and APD_{90} in Con iPSC-CMs treated with DNJ (Con: n = 8, Con + DNJ: n = 8). Values represent the mean ± SEM. Student's unpaired, two-tailed *t* test. **(C-H)** Quantification of V_{max}, MDP, overshoot, AP amplitude, SD of Peak-Peak Intervals and beating rate among Con iPSC-CMs, Con iPSC-CMs + DNJ (Con: n = 8; Con + DNJ: n = 8). Values represent the mean ± SEM. Student's unpaired, two-tailed *t* test.

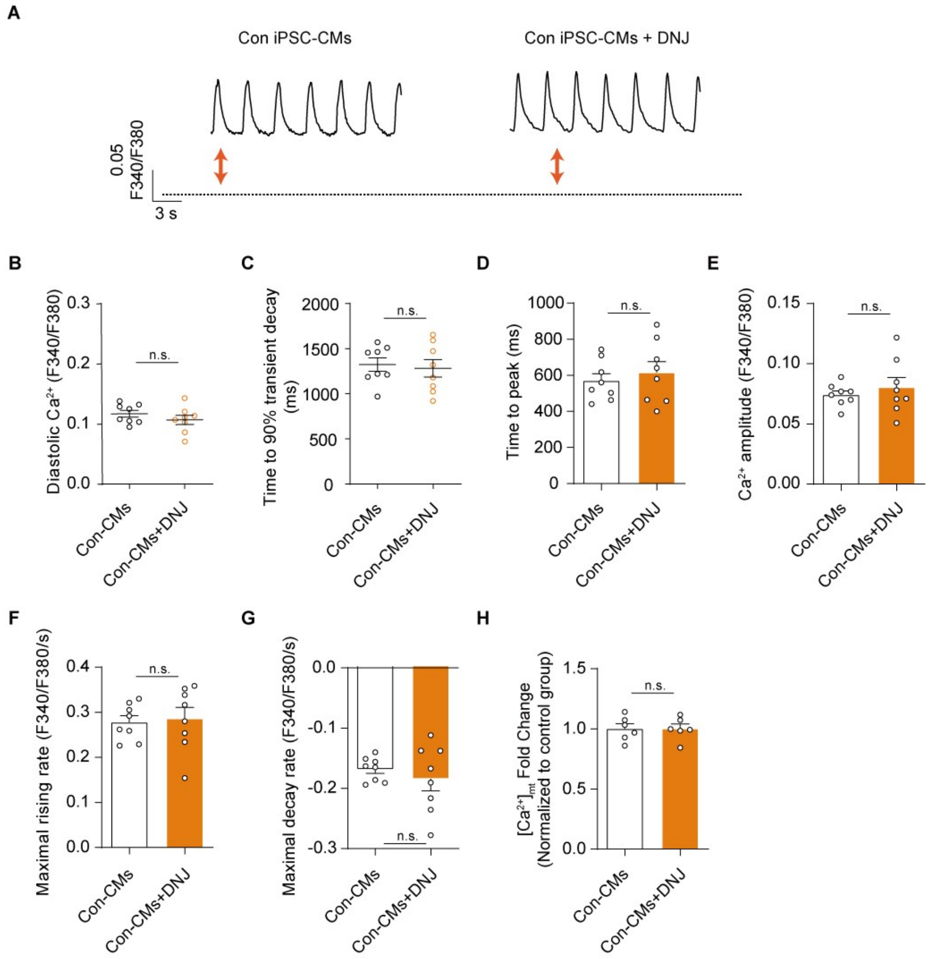

Supplemental Figure 8. The parameters of calcium handling in control iPSC-CMs treated with DNJ. **(A)** Representative raw traces of Fura-2 ratio-metric calcium signaling. **(B and C)** Scatter dot plot to compare diastolic Ca^{2+} **(B)** and decay times **(C)** in Con iPSC-CMs and Con iPSC-CMs + DNJ. n = 8. Values represent the mean ± SEM. Student's unpaired, two-tailed *t* test. **(D-G)** Quantification of time to peak, Ca^{2+} amplitude, maximal rising rate and maximal decay rate among Con iPSC-CMs, Con iPSC-CMs + DNJ. n = 8. Values represent the mean ± SEM. Student's unpaired, two-tailed *t* test. **(H)** Analysis of mitochondrial calcium by RHOD-2 indicators in two groups. n = 3 biologically independent experiments in two lines. Values represent the mean ± SEM. Student's unpaired, two-tailed *t* test.

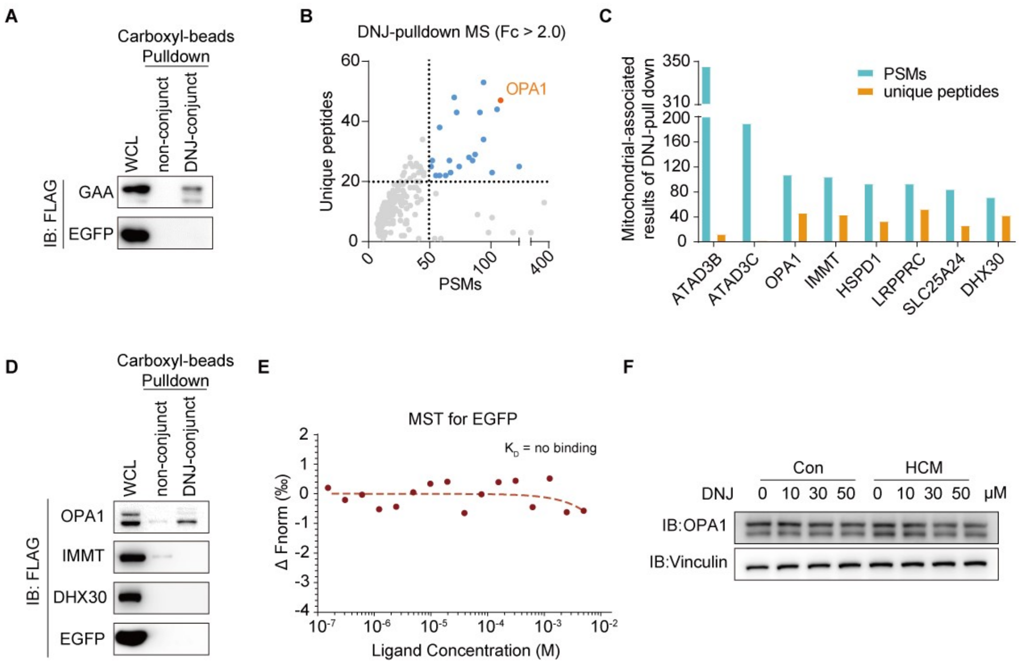

Supplemental Figure 9. Mass spectrum-proteomics analysis and candidate verification. (A) Immunoblot confirmation of the DNJ-binding protein with Flag-His-GAA, with Flag-His-EGFP as the negative control. **(B)** Scatterplot of the captured prey proteins (PSMs (DNJ) / PSMs (control) ≥ 2.0) from DNJ-pull down are plotted with PSMs (x axis) and unique peptide (y axis). Point highlighted in orange represents OPA1. **(C)** List of the potential DNJ targeted protein and appointing PSMs and mitochondrial-associated unique peptides as the indicator. **(D)** Immunoblot confirmation of the potential target for DNJ with Flag-His-OPA1, Flag-His-IMMT and Flag-His-DHX30, with EGFP as the negative control. **(E)** MST assay for the affinity between DNJ and purified EGFP protein. **(F)** Immunoblot confirmation of the relationship between DNJ concentration and OPA1 endogenous expression.

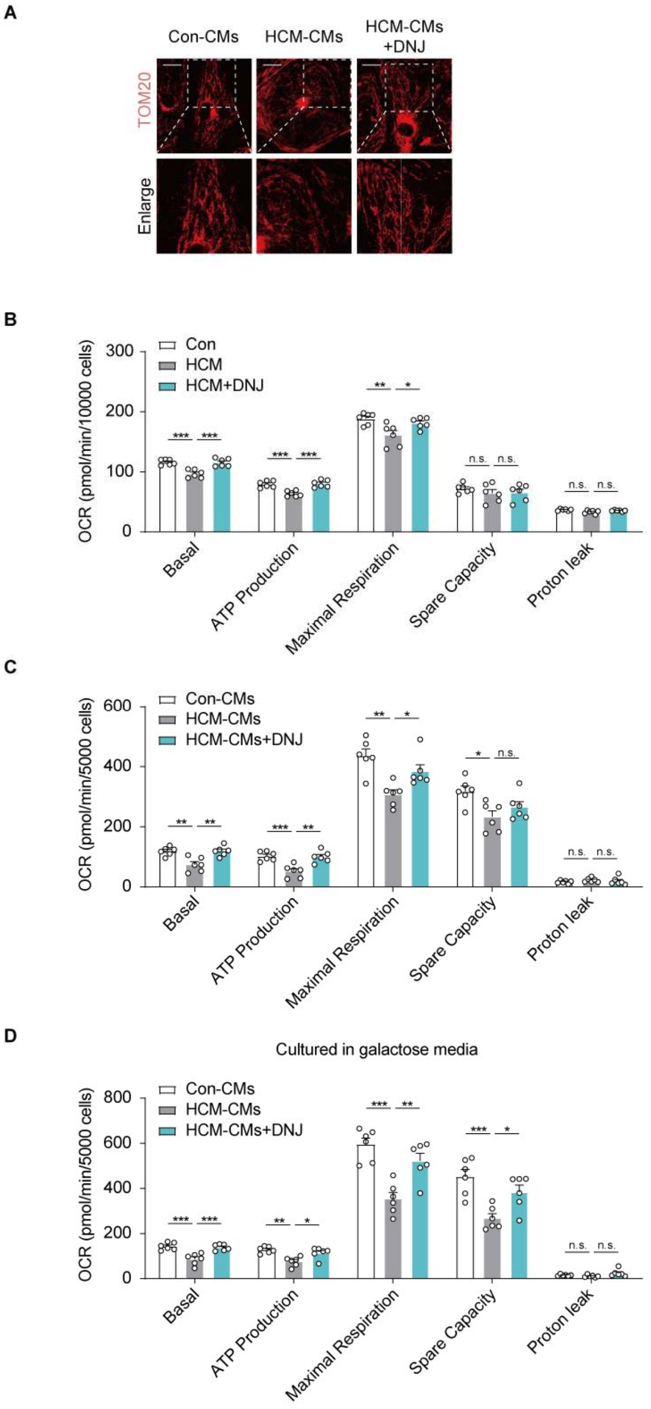

Supplemental Figure 10. DNJ rescues the mitochondrial dysfunction in HCM iPSC-CMs. (A) Mitochondrial networks of Con iPSC-CMs, HCM iPSC-CMs and HCM iPSC-CMs + DNJ. iPSC-CMs were immunolabeled for mitochondria marker TOM20. Scale bar, 10 μm. **(B-D)** Quantification of basal OCR, ATP-linked OCR, maximal OCR, spare capacity and proton leak in cybrids **(B)**, iPSC-CMs **(C)** and galactose cultured iPSC-CMs **(D)**. n = 3 biologically independent experiments. Data are representative of 3 independent experiments. Values represent the mean ± SEM. Two-way ANOVA analysis. *$P < 0.05$, **$P < 0.01$, ***$P < 0.001$.

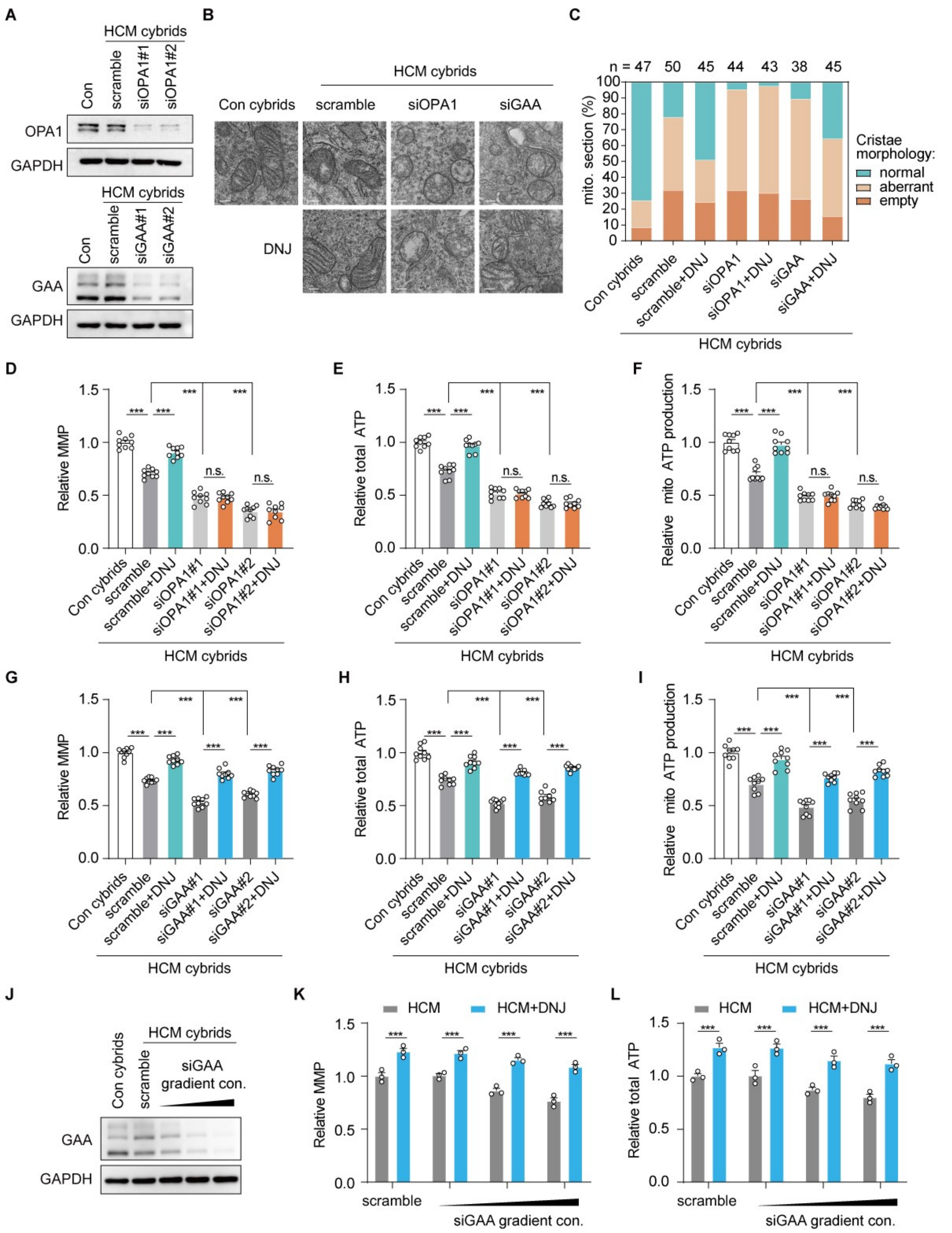

Supplemental Figure 11. The effect of DNJ on mitochondrial function in HCM cybrids with OPA1 or GAA knockdown. **(A)** Immunoblotting total protein expression of OPA1 and GAA in HCM cybrids treated with OPA1 or GAA siRNA. **(B)** Representative TEM recordings of Con, HCM scramble, HCM scramble + DNJ, HCM siOPA1, HCM siOPA1 + DNJ, HCM siGAA and HCM siGAA + DNJ. Scale bar, 200 nm. **(C)** Quantification of the overall cristae morphology on TEM recordings. **(D-F)** Mitochondrial membrane potential analysis, total ATP and mitochondrial ATP production were measured with OPA1 knockdown. n = 3 biologically independent experiments in three lines. Values represent the mean ± SEM. One-way ANOVA followed by Tukey's test. ***P < 0.001. **(G-I)** Mitochondrial membrane potential

analysis, total ATP and mitochondrial ATP production were measured with GAA knockdown. n = 3 biologically independent experiments in three lines. Values represent the mean ± SEM. One-way ANOVA followed by Tukey's test. ***P < 0.001. **(J)** Immunoblotting total protein expression of GAA with gradient knockdown assay. **(K and L)** Mitochondrial membrane potential analysis and total ATP production were measured with gradient knockdown assay. n = 3 biologically independent experiments. Values represent the mean ± SEM. Two-way ANOVA analysis. ***P < 0.001. Data are representative of 3 independent experiments. Con., concentration.

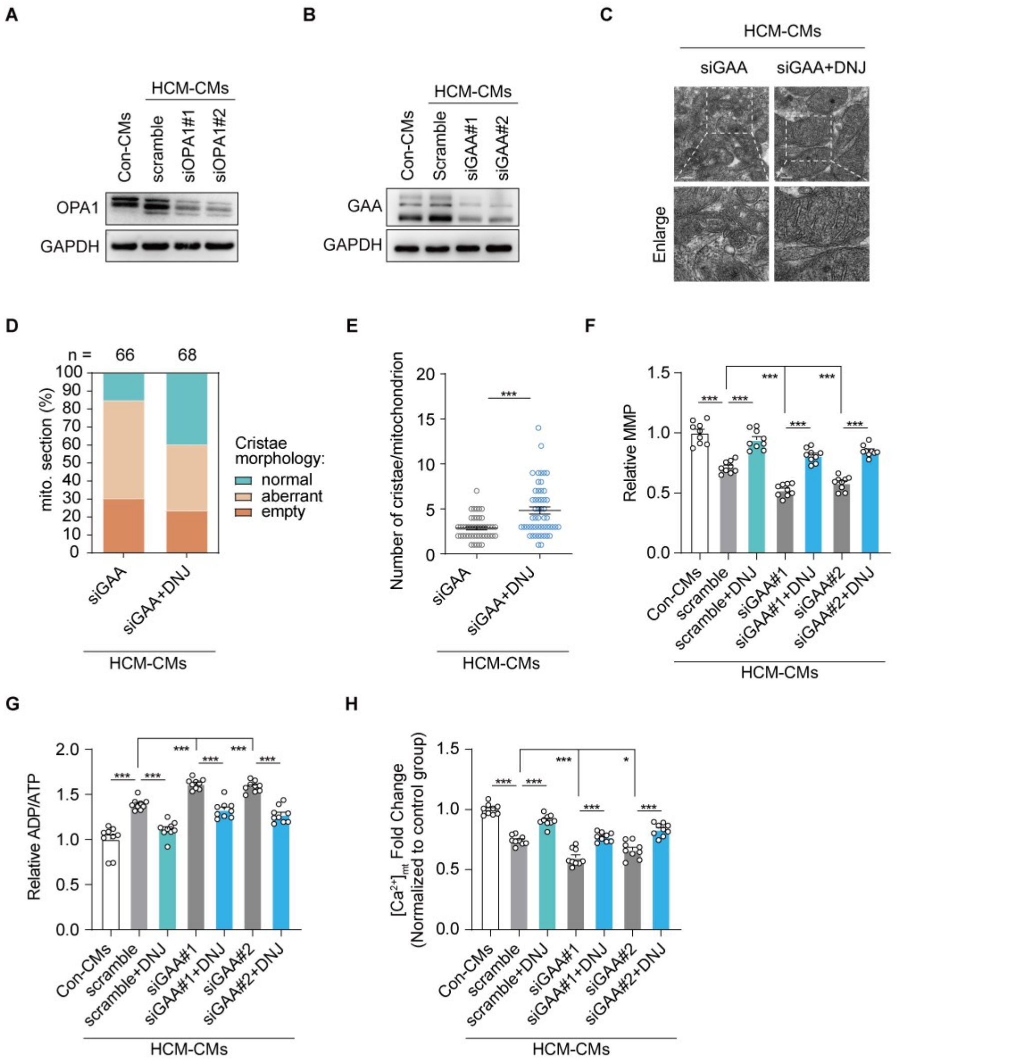

Supplemental Figure 12. DNJ restores mitochondrial function in GAA-knockdown HCM iPSC-CMs. (A) Immunoblotting total protein expression of OPA1 in HCM iPSC-CMs treated with OPA1 siRNA. **(B)** Immunoblotting total protein expression of GAA in HCM iPSC-CMs treated with GAA siRNA. **(C)** Representative TEM recordings of siGAA and siGAA + DNJ. Scale bar, 200 nm. **(D)** Quantification of the overall cristae morphology on TEM recordings. **(E)** Quantification of mitochondrial cristae number. HCM siGAA: n = 46, HCM siGAA + DNJ: n = 52 biologically independent mitochondria. Values represent the mean ± SEM. Student's unpaired, two-tailed *t* test. ***$P < 0.001$. **(F)** Mitochondrial membrane potential was measured. n = 3 biologically independent experiments in two lines. Values represent the mean ± SEM. One-way ANOVA followed by Tukey's test. ***$P < 0.001$. **(G)** ADP/ATP ratio was measured using a bioluminescent assay system. n = 3 biologically independent experiments in two lines. Values represent the mean ± SEM. One-way ANOVA followed by Tukey's test. ***$P < 0.001$. **(H)** Analysis of mitochondrial calcium by RHOD-2 indicators in seven groups. n = 3 biologically independent experiments. Values represent the mean ± SEM. One-way ANOVA followed by Tukey's test. *$P < 0.05$, ***$P < 0.001$.

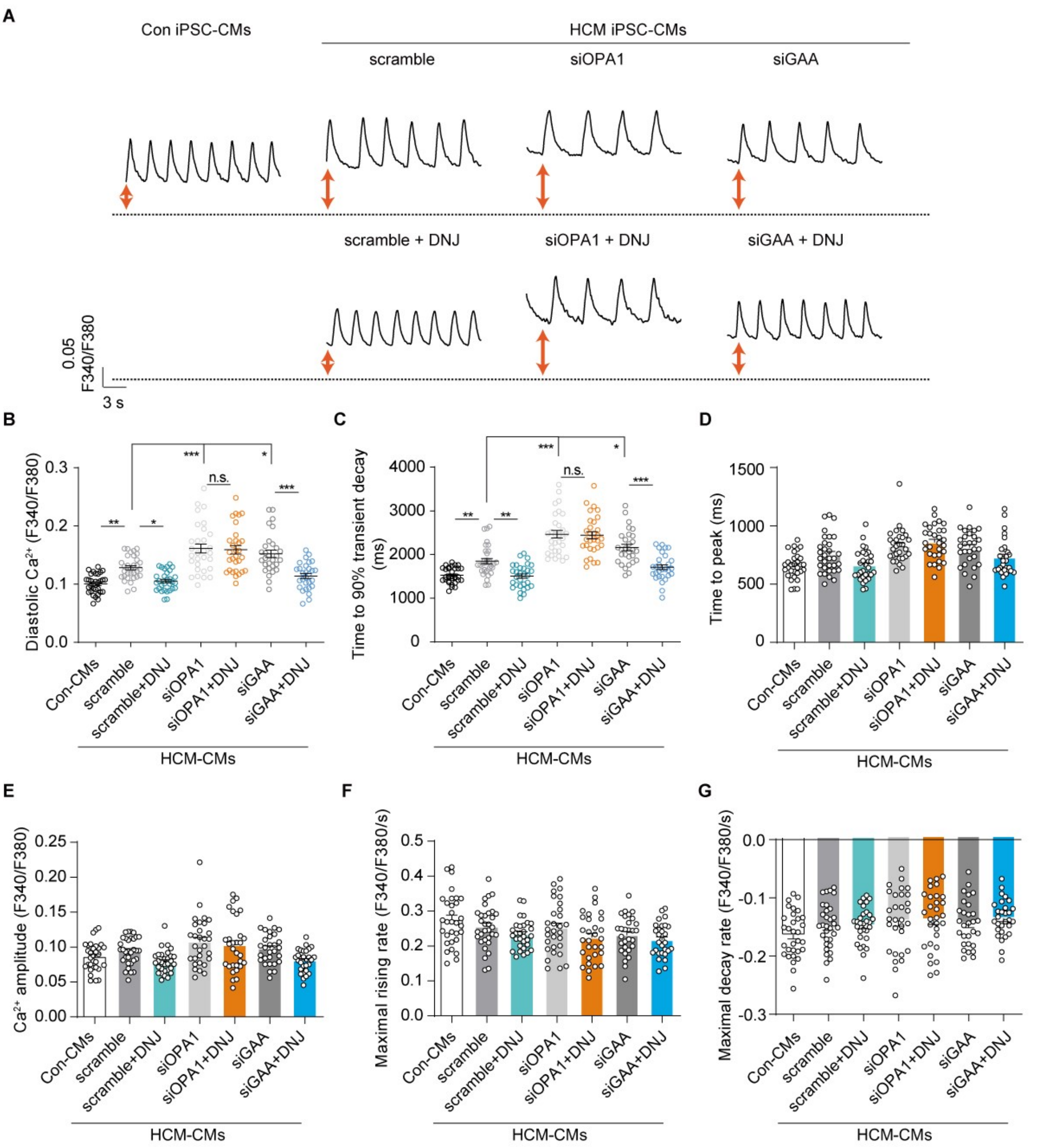

Supplemental Figure 13. The parameters of calcium handling in HCM iPSC-CMs treated with DNJ with OPA1 or GAA knockdown.

(A) Representative raw traces of Fura-2 ratio-metric calcium signaling. **(B and C)** Scatter dot plot to compare diastolic Ca^{2+} **(B)** and decay times **(C)** in Con-CMs, HCM-CMs scramble, scramble + DNJ, siOPA1, siOPA1 + DNJ, siGAA and siGAA + DNJ groups. Con: n = 31, HCM scramble: n = 32, HCM scramble + DNJ: n = 30, HCM siOPA1: n = 30, HCM siOPA1 + DNJ: n = 30, HCM siGAA: n = 29, HCM siGAA + DNJ: n = 29. Values represent the mean ± SEM. One-way ANOVA followed by Tukey's test. *$P < 0.05$, **$P < 0.01$, ***$P < 0.001$. **(D-G)** Quantification of time to peak, Ca^{2+} amplitude, maximal rising rate and maximal decay rate. Con: n = 31, HCM scramble: n = 32, HCM scramble + DNJ: n = 30, HCM siOPA1: n = 30, HCM siOPA1 + DNJ: n = 30, HCM siGAA: n = 29, HCM siGAA + DNJ: n = 29. Values represent the mean ± SEM. For each group, data were collected from two different iPSC lines and at least three batches of differentiation.

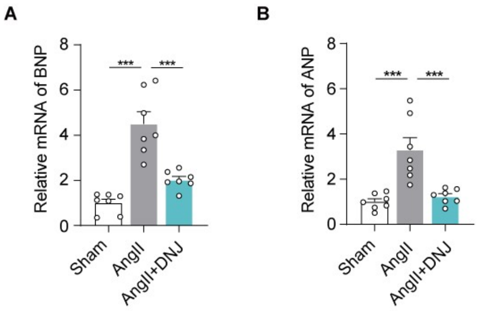

Supplemental Figure 14. Relative mRNA levels of hypertrophy marker genes. (A and B) Relative mRNA levels of hypertrophy marker genes including BNP and ANP in heart tissues. Values represent the mean ± SEM. n = 7 biologically independent samples of cardiac mice tissues. One-way ANOVA followed by Tukey's test. ***$P < 0.001$.

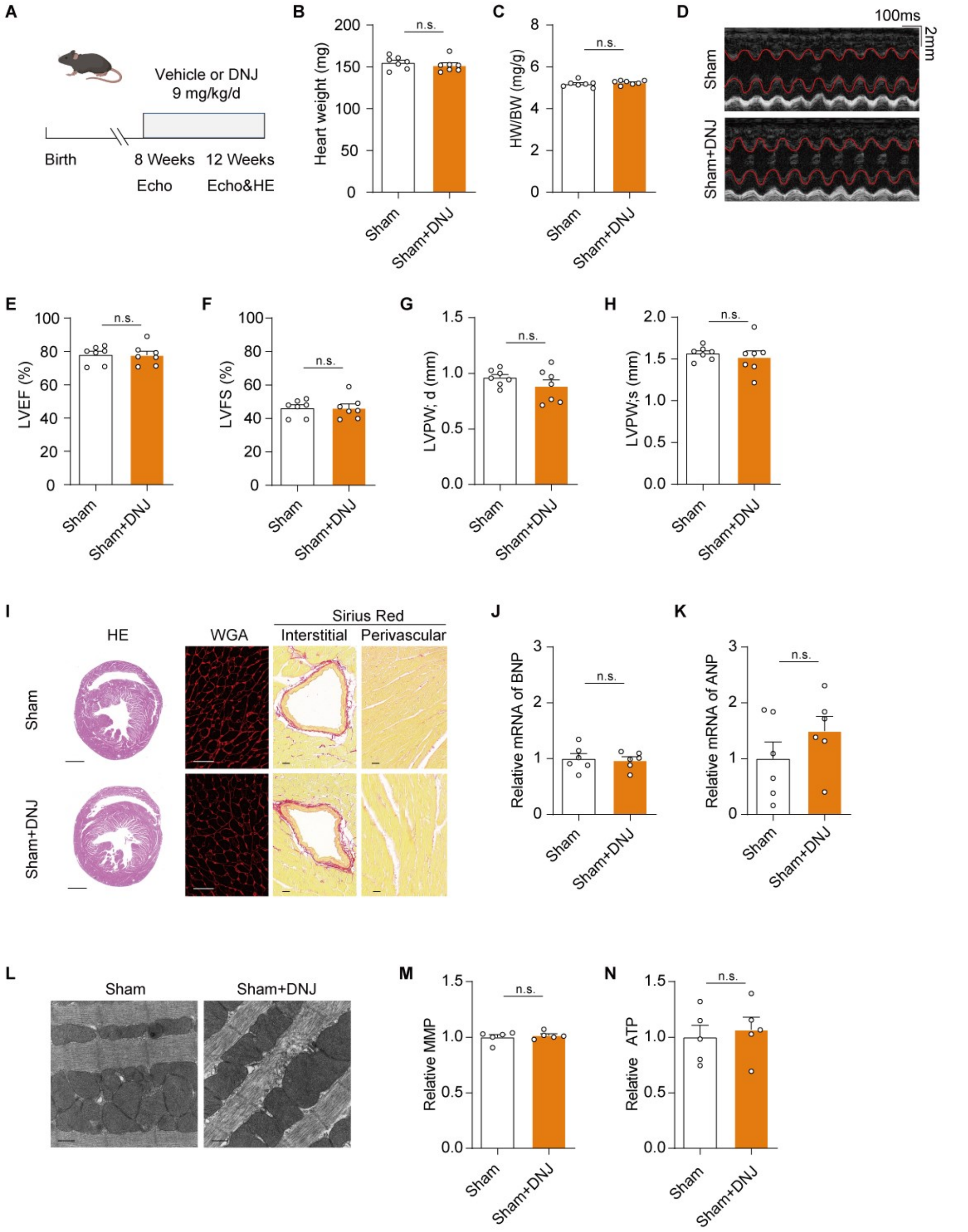

Supplemental Figure 15. The effect of DNJ in mice. (A) Schematic diagram depicting the experimental strategy for DNJ treatment. Echo: echocardiographic assessments, HE: hematoxylin-eosin staining. **(B)** Heart weight. n = 7 mice. Values represent the mean ± SEM. Student's unpaired, two-tailed *t* test. **(C)** Heart weight (HW) normalized to body weight (BW). n = 7 mice. Values represent the mean ± SEM. Student's unpaired, two-tailed *t* test. **(D)** Echocardiograms of sham and sham + DNJ group. **(E-H)** Echocardiography parameters (EF, FS and LVPW) of mice with DNJ- or vehicle-treatment. n = 7 mice. Values represent the mean ± SEM. Student's unpaired, two-tailed *t* test. **(I)** Representative images of hematoxylin-eosin (HE) staining, wheat germ agglutinin (WGA) staining (cardiac hypertrophy) and Picrosirius red staining (fibrosis) of Sham and Sham + DNJ. Scale bar, 1000 μm (HE); 30 μm

(WGA); 20 μm (Sirus Red). **(J and K)** Relative mRNA levels of hypertrophy marker genes including BNP and ANP in heart tissues. Values represent the mean ± SEM. n = 6 biologically independent samples of cardiac mice tissues. Student's unpaired, two-tailed *t* test. **(L)** Representative TEM recordings of Sham and Sham + DNJ. Scale bar, 500 μm. **(M and N)** Cardiac cells were isolated from the cardiac tissues of each mouse, separately. Their relative MMP and ATP levels were then measured. n = 5 biologically independent samples of cardiac mice tissues. Student's unpaired, two-tailed *t* test.

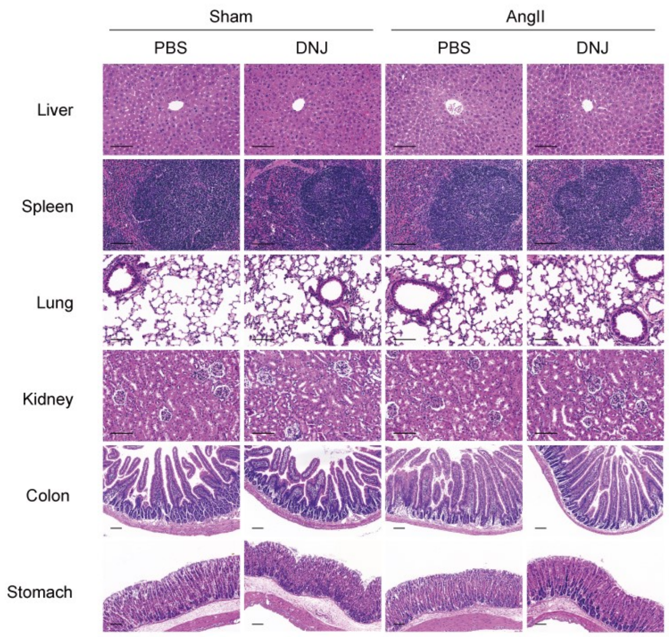

Supplemental Figure 16. The representative H&E staining of tissue slices from different organs of mice treated with DNJ. Scale bar, 100 μm.

Supplemental Tables

Supplemental Table 1. Mitochondrial chemical molecules

Name	Source	Identifier
Tempol	Targetmol	T6699
Leonurine hydrochloride	Targetmol	T4080
Saikosaponin D	Targetmol	T3913
VERBENALIN	Targetmol	T3890
1-Deoxynojirimycin (DNJ)	Targetmol	T3675
Hydroxysafflor yellow A	Targetmol	T3674
Galangin	Targetmol	T3668
RSL3	Targetmol	T3646
Amentoflavone	Targetmol	T3417
Secoisolariciresinoldiglucoside	Targetmol	T3415
Honokiol	Targetmol	T3001
(-)-Epigallocatechin Gallate	Targetmol	T2988
Astragalus polyphenols	Targetmol	T2964
Alphalipoic acid	Targetmol	T2930
Osthole	Targetmol	T2848
Puerarin (Kakonein)	Targetmol	T2815
Coenzyme Q10 (CoQ10)	Targetmol	T2796
Sanguinarine	Targetmol	T2781
(+)-Catechin Hydrate	Targetmol	T2778
Salvianolic acid B	Targetmol	T2727
Tetramethylpyrazine	Targetmol	T2722
Salidroside	Targetmol	T2717
Alda-1	Targetmol	T2662
O-Acetyl-L-carnitine hydrochloride	Targetmol	T2563
Paeoniflorin	Targetmol	T2230
Erastin	Targetmol	T1765
Trolox	Targetmol	T1710
7-Hydroxycoumarin	Targetmol	T1693
Melatonine	Targetmol	T1659

Vitamin E	Targetmol	T1648
Rebamipide	Targetmol	T1562
Resveratrol	Targetmol	T1558
Nicotinic acid	Targetmol	T0879
Idebenone	Targetmol	T0412
Edaravone	Targetmol	T0407
Sodium 4-aminosalicylate dihydrate	Targetmol	T0020
Oxymatrine	Targetmol	T2754
Asiatic acid	Targetmol	T2827
Cyclosporin A	Targetmol	T0945
Methylene Blue	Targetmol	T6888
Creatine phosphate disodium salt	Targetmol	T3262

Supplemental Table 2. Effect of DNJ on action potentials recorded from HCM iPSC-CMs

	Con iPSC-CMs	HCM iPSC-CMs	HCM iPSC-CMs +DNJ
Beating (beats/min)	82 ± 3	57 ± 4	75 ± 4
MDP (mV)	-58.6 ± 1.0	-59.2 ± 0.9	-60.4 ± 0.9
Overshoot (mV)	49.1 ± 0.6	50.6 ± 1.1	47.7 ± 1.3
APA (mV)	107.7 ± 1.2	109.8 ± 1.5	108.1 ± 1.3
APD_{50}	248.1 ± 13.1	334.3 ± 14.5	273.3 ± 13.8
APD_{70}	274.0 ± 13.5	377.2 ± 15.8	302.2 ± 14.6
APD_{90}	297.7 ± 13.7	413.9 ± 16.4	325.7 ± 14.6
V_{max} (V/s)	15.5 ± 1.0	16.7 ± 0.9	14.8 ± 1.2

Data shown as mean ± SEM. N: as indicated in related figure panel.

Supplemental Table 3. Effect of Astra and Verb on action potentials recorded from HCM iPSC-CMs

	HCM iPSC-CMs	HCM iPSC-CMs +Astra	HCM iPSC-CMs +Ver
Beating (beats/min)	56 ± 5	52 ± 7	59 ± 4
MDP (mV)	-60.6 ± 1.5	-60.6 ± 2.6	-63.0 ± 1.5
Overshoot (mV)	46.0 ± 1.6	46.4 ± 1.3	46.0 ± 2.0
APA (mV)	106.6 ± 1.9	107.0 ± 3.0	109.1 ± 1.9
APD_{50}	305.8 ± 27.0	301.6 ± 23.6	333.5 ± 18.4
APD_{70}	339.6 ± 28.5	333.2 ± 23.5	375.3 ± 21.0
APD_{90}	368.2 ± 30.2	361.6 ± 23.4	404.5 ± 23.4
V_{max} (V/s)	11.5 ± 1.4	10.2 ± 0.7	11.3 ± 0.6

Data shown as mean ± SEM. N: as indicated in related figure panel.

Supplemental Table 4. Effect of DNJ on action potentials recorded from control iPSC-CMs

	Con iPSC-CMs	Con iPSC-CMs +DNJ
Beating (beats/min)	85 ± 8	70 ± 9
MDP (mV)	-58.5 ± 1.2	-57.4 ± 1.4
Overshoot (mV)	49.8 ± 1.5	49.3 ± 1.7
APA (mV)	108.3 ± 1.2	106.7 ± 2.8
APD_{50}	239.3 ± 19.9	236.1 ± 22.5
APD_{70}	263.1 ± 21.1	259.0 ± 24.8
APD_{90}	286.0 ± 21.9	280.2 ± 26.4
V_{max} (V/s)	13.4 ± 0.8	13.9 ± 1.6

Data shown as mean ± SEM. N: as indicated in related figure panel.

Supplemental Table 5. Protein identification results for DNJ pulldown assay

This part of data is provided in the attachment.

Supplemental Table 6. Baseline echo parameters of sham, AngII and DNJ groups

	Sham	AngII	AngII+DNJ
Heart rate (beats/min)	542 ± 11	551 ± 9	534 ± 4
IVS; d (mm)	0.822 ± 0.017	0.863 ± 0.040	0.953 ± 0.041
LVID; d (mm)	3.308 ± 0.137	3.297 ± 0.140	3.266 ± 0.113
LVPW; d (mm)	0.900 ± 0.033	0.895 ± 0.038	0.886 ± 0.052
IVS; s (mm)	1.306 ± 0.036	1.332 ± 0.048	1.325 ± 0.040
LVID; s (mm)	1.847 ± 0.109	1.840 ± 0.133	1.847 ± 0.108
LVPW; s (mm)	1.281 ± 0.040	1.288 ± 0.067	1.288 ± 0.052
LVEF (%)	76.6 ± 1.6	76.3 ± 2.5	75.8 ± 1.9
LVFS (%)	44.4 ± 1.5	44.5 ± 2.4	43.7 ± 1.7
LVEDV (μL)	45.156 ± 4.379	44.822± 4.691	43.586 ± 3.841
LVESV (μL)	10.826 ± 1.442	10.919 ± 1.802	10.793 ± 1.632

Data shown as mean ± SEM. N: as indicated in related figure panel.

IVS;d = Interventricular septum thickness at diastole;

LVID; d = Left ventricular internal diameter at end-diastole;

LVPW; d = Left ventricular posterior wall thickness at diastole;

IVS; s = Interventricular septum thickness at systole;

LVID; s = Left ventricular internal diameter at end-systole;

LVPW; s = Left ventricular posterior wall thickness at systole;

LVEF = Left ventricular ejection fraction;

LVFS = Left ventricular fraction shortening;

LVEDV = Left ventricular end-diastolic volume;

LVESV = Left ventricular end-systole volume.

Supplemental Table 7. Echo parameters of sham, AngII and DNJ groups after 4-week treatment

	Sham	AngII	AngII+DNJ
Heart rate (beats/min)	539 ± 13	515 ± 13	528 ± 9
IVS; d (mm)	0.884 ± 0.040	1.267 ± 0.041	0.909 ± 0.074**
LVID; d (mm)	3.227 ± 0.050	2.484 ± 0.084	3.308 ± 0.090***
LVPW; d (mm)	0.910 ± 0.031	1.344 ± 0.035	0.932 ± 0.063***
IVS; s (mm)	1.312 ± 0.019	1.521 ± 0.034	1.348 ± 0.057*
LVID; s (mm)	1.822 ± 0.059	1.777 ± 0.064	2.019 ± 0.069*
LVPW; s (mm)	1.301 ± 0.030	1.542 ± 0.054	1.321 ± 0.050*
LVEF (%)	75.9 ± 1.3	57.0 ± 1.5	70.6 ± 1.1***
LVFS (%)	43.6 ± 1.2	28.5 ± 1.0	39.0 ± 0.8***
LVEDV (μL)	41.977 ± 1.609	22.270 ± 1.787	44.721 ± 2.859***
LVESV (μL)	10.178 ± 0.842	9.558 ± 0.843	13.241 ± 1.170*

*$P < 0.05$, **$P < 0.01$, ***$P < 0.001$ by 2-tailed unpaired student *t* test. AngII *vs.* AngII + DNJ. Data shown as mean ± SEM. N: as indicated in related figure panel.

Supplemental Table 8. Baseline echo parameters of sham and DNJ groups

	Sham	Sham + DNJ
Heart rate (beats/min)	518 ± 13	521 ± 7
IVS; d (mm)	0.796 ± 0.027	0.817 ± 0.031
LVID; d (mm)	3.693 ± 0.065	3.658 ± 0.069
LVPW; d (mm)	0.850 ± 0.065	0.845 ± 0.045
IVS; s (mm)	1.376 ± 0.060	1.419 ± 0.026
LVID; s (mm)	2.070 ± 0.087	2.024 ± 0.062
LVPW; s (mm)	1.457 ± 0.075	1.398 ± 0.032
LVEF (%)	75.8 ± 1.8	76.4 ± 1.7
LVFS (%)	44.0 ± 1.7	44.6 ± 1.7
LVEDV (μL)	58.081 ± 2.475	56.806 ± 2.591
LVESV (μL)	14.218 ± 1.400	13.294 ± 1.027

Data shown as mean ± SEM. N: as indicated in related figure panel.

Supplemental Table 9. Echo parameters of sham and DNJ groups after 4-week treatment

	Sham	Sham + DNJ
Heart rate (beats/min)	542 ± 11	524 ± 16
IVS; d (mm)	0.918 ± 0.023	0.873 ± 0.025
LVID; d (mm)	3.691 ± 0.062	3.690 ± 0.058
LVPW; d (mm)	0.963 ± 0.027	0.886 ± 0.056
IVS; s (mm)	1.582 ± 0.045	1.504 ± 0.070
LVID; s (mm)	1.982 ± 0.072	1.978 ± 0.076
LVPW; s (mm)	1.568 ± 0.030	1.521 ± 0.077
LVEF (%)	78.1 ± 2.0	77.8 ± 2.3
LVFS (%)	46.3 ± 1.9	46.2 ± 2.5
LVEDV (μL)	57.984 ± 2.349	57.960 ± 2.236
LVESV (μL)	12.684 ± 1.262	12.590 ± 1.144

Data shown as mean ± SEM. N: as indicated in related figure panel.

REFERENCES

1. Liu Z, Song Y, Li D, He X, Li S, Wu B, et al. The novel mitochondrial 16S rRNA 2336T>C mutation is associated with hypertrophic cardiomyopathy. *J Med Genet.* 2014;51(3):176-84.
2. Li D, Sun Y, Zhuang Q, Song Y, Wu B, Jia Z, et al. Mitochondrial dysfunction caused by m.2336T>C mutation with hypertrophic cardiomyopathy in cybrid cell lines. *Mitochondrion.* 2019;46:313-20.
3. Li S, Pan H, Tan C, Sun Y, Song Y, Zhang X, et al. Mitochondrial Dysfunctions Contribute to Hypertrophic Cardiomyopathy in Patient iPSC-Derived Cardiomyocytes with MT-RNR2 Mutation. *Stem Cell Reports.* 2018;10(3):808-21.
